# Macromolecular Crowding, Phase Separation, and Homeostasis in the Orchestration of Bacterial Cellular Functions

**DOI:** 10.1021/acs.chemrev.3c00622

**Published:** 2024-02-08

**Authors:** Begoña Monterroso, William Margolin, Arnold J. Boersma, Germán Rivas, Bert Poolman, Silvia Zorrilla

**Affiliations:** † Department of Structural and Chemical Biology, Centro de Investigaciones Biológicas Margarita Salas, Consejo Superior de Investigaciones Científicas (CSIC), 28040 Madrid, Spain; ‡ Department of Microbiology and Molecular Genetics, McGovern Medical School, UTHealth-Houston, Houston, Texas 77030, United States; § Cellular Protein Chemistry, Bijvoet Centre for Biomolecular Research, Faculty of Science, Utrecht University, Padualaan 8, 3584 CH Utrecht, The Netherlands; ∥ Department of Biochemistry, University of Groningen, Nijenborgh 4, 9747 AG Groningen, The Netherlands

## Abstract

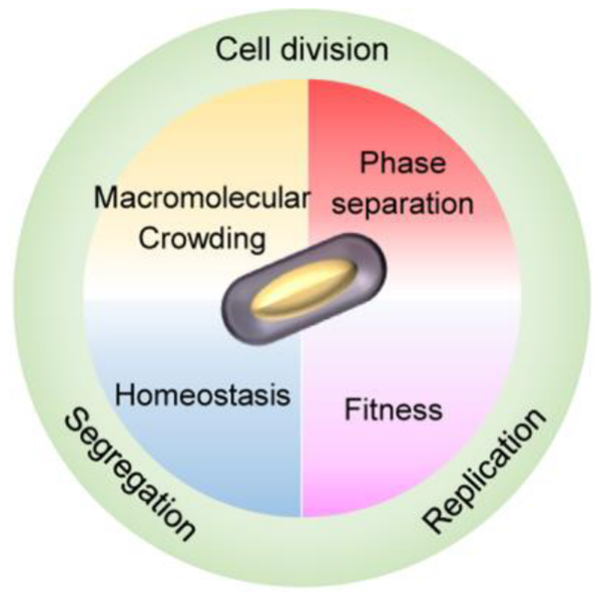

Macromolecular crowding affects the activity of proteins and functional macromolecular complexes in all cells, including bacteria. Crowding, together with physicochemical parameters such as pH, ionic strength, and the energy status, influences the structure of the cytoplasm and thereby indirectly macromolecular function. Notably, crowding also promotes the formation of biomolecular condensates by phase separation, initially identified in eukaryotic cells but more recently discovered to play key functions in bacteria. Bacterial cells require a variety of mechanisms to maintain physicochemical homeostasis, in particular in environments with fluctuating conditions, and the formation of biomolecular condensates is emerging as one such mechanism. In this work, we connect physicochemical homeostasis and macromolecular crowding with the formation and function of biomolecular condensates in the bacterial cell and compare the supramolecular structures found in bacteria with those of eukaryotic cells. We focus on the effects of crowding and phase separation on the control of bacterial chromosome replication, segregation, and cell division, and we discuss the contribution of biomolecular condensates to bacterial cell fitness and adaptation to environmental stress.

## 1 Introduction

In bacteria and archaea, as in eukaryotes, macromolecular and supramolecular assemblies are at the core of all biochemical processes enabling cells to carry out their activities. Many of these assemblies are dynamic structures whose functions depend upon the ability of their constituent macromolecules to reversibly dissociate and reassociate. These dynamics regulate the biochemical activity of the interacting networks and/or facilitate structural modifications linked to function. The bacterial cell cycle machinery is an excellent example of an organized structure in which molecular assemblies involved in the initiation of replication, chromosome segregation, and cell division coordinate with one another for bacterial survival and genomic integrity.^1^

Although the intact cell represents an attractive system for studying the structural and functional organization of subcellular machines, interpreting the results obtained from such studies must consider that these interacting systems function inside the cell in a heterogeneous and highly volume-occupied or crowded environment.^2−6^ These microenvironments may influence the reactivity and location of proteins and other biological macromolecules involved in essential processes, thus acting as nonspecific modulating factors of bacterial cellular functions. The purpose of this review is to emphasize that the mode of operation of critical bacterial cell cycle events depends not only on the specific molecular interactions between their components but also on nonspecific interactions with elements of their intracellular microenvironments.

The cell interior of a simple organism such as *Escherichia coli* is highly crowded, as approximately 20–30% of its volume is occupied by macromolecules,^7,8^ although no single macromolecule needs to be highly concentrated for it to function. Therefore, a given protein X in the cytoplasm will be primarily subjected to the influence of excluded volume effects due to crowding by soluble macromolecules, leading to preferential (size- and shape-dependent) exclusion from highly volume-occupied elements. This exclusion may significantly alter the extent and rate of macromolecular reactions mediated by X. High macromolecular crowding can also drive phase transitions, resulting in the formation of membrane-free biomolecular condensates. During its life cycle, X will be subjected to additional background interactions with elements of its immediate surroundings, including ribosomes [ribosomal RNAs (rRNAs) contain most of the nucleic acid in a bacterial cell], the nucleoid (within which X will encounter a high local concentration of DNA and nucleoid-associated proteins), and the cytoplasmic membrane (within which X will encounter a high local concentration of lipids and membrane proteins).

These background interactions (nonspecific interactions between macromolecular reactants and other constituents of the local environment) can lead to excluded-volume effects (and beyond) due to natural crowding. These interactions also can result in partitioning between immiscible phases and surface adsorption that collectively contribute to the total free energy of the system. These effects thereby substantially influence the energetics, dynamics, and spatiotemporal organization of macromolecular interactions and reactions. The relative contribution of these effects on macromolecular reactivity likely differs between each of the intracellular environments.^5,9^

### 1.1 Macromolecular Crowding

**Figure 1:**
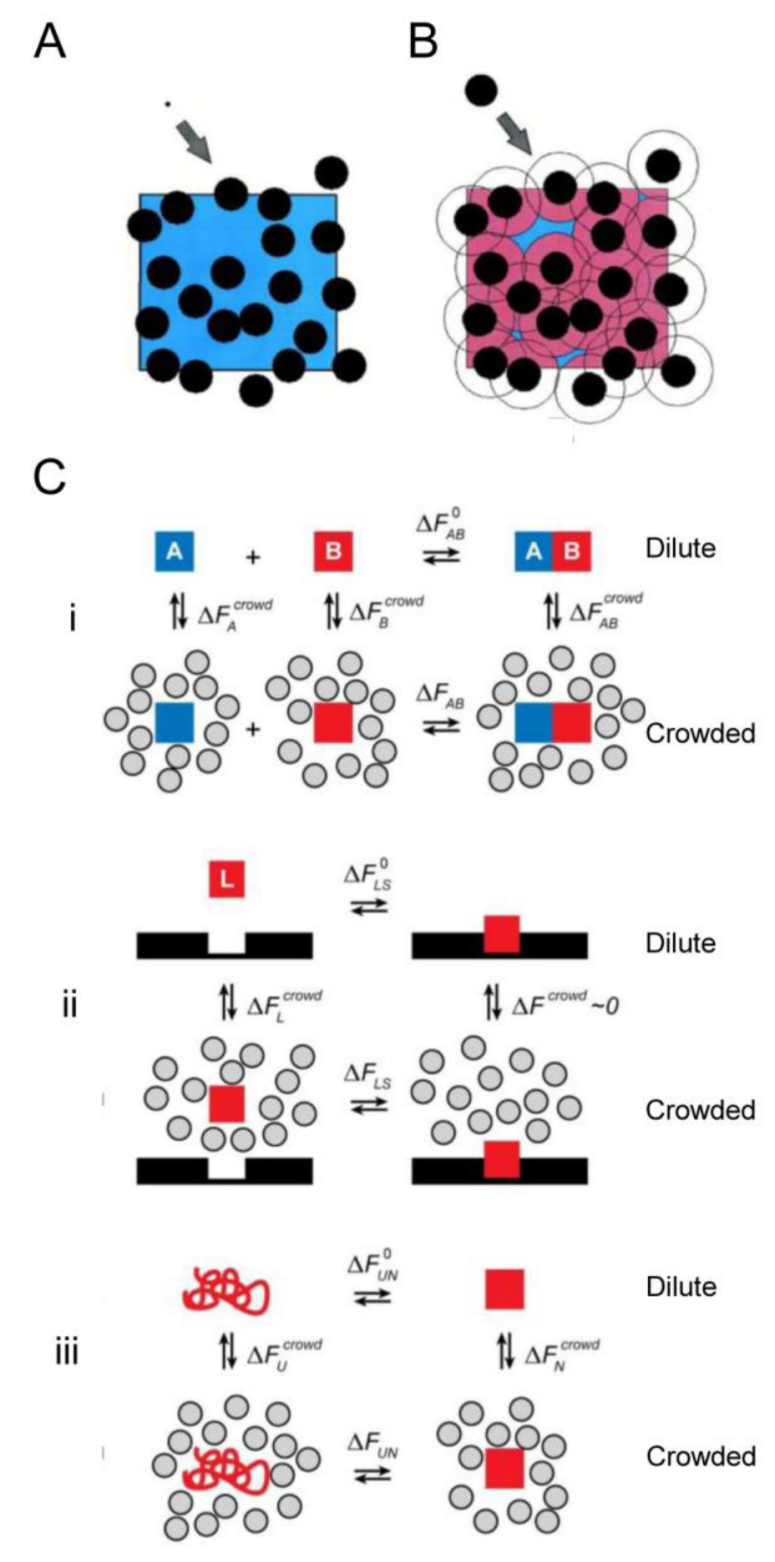
**Molecular effects of crowding.** (A and B) Crowding increases the chemical potential (activity) of a test protein (T) in solution in a size- and shape-dependent manner. The squares represent a volume element containing spherical macromolecules (in black) that occupy about 30% of the total volume, as is typical of bacterial cytoplasm. The available volume to the center of T is indicated by the blue-colored regions, and its complement (in red) is referred to as the excluded volume. If T is very small relative to the background macromolecules (A), the available volume is almost equal to the total unoccupied volume. But if the size of T is comparable to that of the other solutes (B), the available volume is considerably smaller and the contribution of steric repulsion to reduced entropy and increased free energy is correspondingly greater. Clearly, one of the ways in which the system can reduce its free energy is to maximize the available volume (or, alternatively, to minimize the excluded volume). Reproduced from ref (20). Copyright 2001 Elsevier Inc. under Creative Commons CC-BY license [https://creativecommons.org/licenses/by/4.0/]. (C) Thermodynamic cycles illustrating how dilute or crowded solutions determine free energy differences for (i) a binary heteroassociation between molecules A and B, (ii) a ligand L interacting with its binding site, and (iii) a two-state folding of a protein (red). Reproduced from ref (15). Copyright 2008 Annual Reviews.

The primary element of intracellular complexity is the presence of locally high concentrations of multiple macromolecular species. The importance of these background interactions arising from steric repulsion in volume-occupied native-like media lies in their generality; they are universally present, independently of the presence or absence of other types of interactions.^9−11^ Crowding refers to the amount of free energy required to transfer a macromolecule from a dilute solution to a crowded environment. This is equivalent to the amount of energy expended to create a cavity large enough to accommodate the introduced macromolecule (the entropic cost of changing the available volume around a macromolecule).^12,13^ Macromolecular aggregates exclude less volume to other macromolecules than isolated molecules, so it is less costly (in free energy) to add an *n*-mer to a crowded fluid than *n* monomers (Figure 1). Therefore, a fundamental chemical consequence of crowding is the nonspecific enhancement of reactions and processes leading to a reduction of total excluded volume. These reactions include the formation of macromolecular complexes in solution, binding of macromolecules to surface sites, formation of insoluble aggregates, and compaction or folding of proteins^5,9,11,14^ (Figure 1C). These predictions have been experimentally confirmed at physiologically significant regimes of volume occupancy (on the order of 10% or more), using a variety of macromolecules with different properties as crowders (for a detailed description of crowders and their use, we refer the readers to these comprehensive reviews^5,10,15,16^). Interestingly, the impact of the configurational entropic effects on the conformation of proteins has been used to design fluorescence-based crowding sensors.^17−19^ When the crowding (excluded volume) increases, the sensor takes on a more compact shape, which leads to increased Förster resonance energy transfer (FRET) from cerulean (CFP) to citrine (YFP).

Significantly, the expected magnitude of crowding effects increases rapidly as the size of the tracer (protein) species increases relative to the size of the crowding species.^9,12^ Therefore, the concerted formation of a large oligomer would be much more sensitive to excluded volume effects than the formation of a homo- or hetero-dimer, as observed experimentally (refs (5 and 9) and references therein). Along these lines, the most significant effects of crowding include decreasing the equilibrium solubility of macromolecules, with an increasing tendency to condense and enhance the formation of higher-order protein assemblies.^21−24^ This can also induce the spontaneous alignment and bundling of self-assembling fibers, particularly relevant for cytoskeletal organization.^23,25,26^

As the cell interior is far more complex than systems studied theoretically or experimentally *in vitro*, the potential implications of additional specific and nonspecific interactions, other than volume exclusion, on macromolecular reactivity in crowded environments has been contemplated since the early investigations on crowding.^27,28^ More recent studies have shown that additional attractive interactions between background molecules and the reactants studied could compensate (to a varying degree) for the repulsive steric interaction due to volume exclusion (refs (29−32) and references therein). While excluded-volume effects are ubiquitous, the impact of compensating attractive interactions is highly variable and system-dependent, as they vary with the chemical nature of the interacting species and the type of reactions studied. In this regard, analyses of the effect of crowding composition on protein solubility and fiber formation have revealed that when the aggregating protein is small relative to crowders, attractive protein–crowder interactions can eventually inhibit protein polymer formation (and, likewise, inhibit association of relatively small proteins). However, when the tracer protein is larger than the dominant crowding species, nonspecific attractive interactions between tracer and crowder are likely insufficient to overcome the magnitude of the excluded volume effect, thus promoting polymer formation and aggregation.^31^

Finally, crowding can affect macromolecular reaction rates by two distinct mechanisms (ref (15) and references therein). In the case of slow, transition-state limited reactions, crowding generally increases the association rate constant and has little effect on the dissociation rate constant. In the case of fast reactions, the limiting factor of the association rate is generally the rate of encounter of the reactants, usually dominated by translational diffusion, which decreases monotonically with increased crowding. The combination of these effects may result in a biphasic dependence regime in which the association rate initially increases with crowder concentration, toward reaching a maximum, and then subsequently decreases upon increasing crowding.^15,33^

### 1.2 Macromolecular Partitioning and Liquid–Liquid Phase Separation (LLPS)

A second element of intracellular complexity relates to the presence of multiple microenvironments, resulting in the partitioning of macromolecular species between immiscible phases with different concentrations of each macromolecule in each phase. A variety of membrane-less organelles found during the past decade within the cell interior that cluster specific biomolecules away from their surroundings represent examples of these local microenvironments.^34^ They have been tentatively identified as immiscible liquid phases, which most likely arise through LLPS, a physicochemical process well studied in polymer chemistry. The latter are also linked to the formation of biomolecular condensates, dynamic structures containing a wide range of proteins and nucleic acids. Such condensates are thought to provide special microenvironments in which the rates and equilibria of critical biochemical reactions may be modulated.^35,36^

These condensates have primarily been studied in eukaryotic cells.^34,35^ However, recent progress indicates that they are also assembled in prokaryotic cells where they play key roles.^37^ As bacteria typically lack membrane-bound organelles, phase separation provides a compelling novel mechanism for spatial and functional organization in this domain of life. Chromosome replication and segregation, and their tight coupling to cytokinesis, provide examples of LLPS with implications for bacterial fitness (*vide infra*).

**Figure 2:**
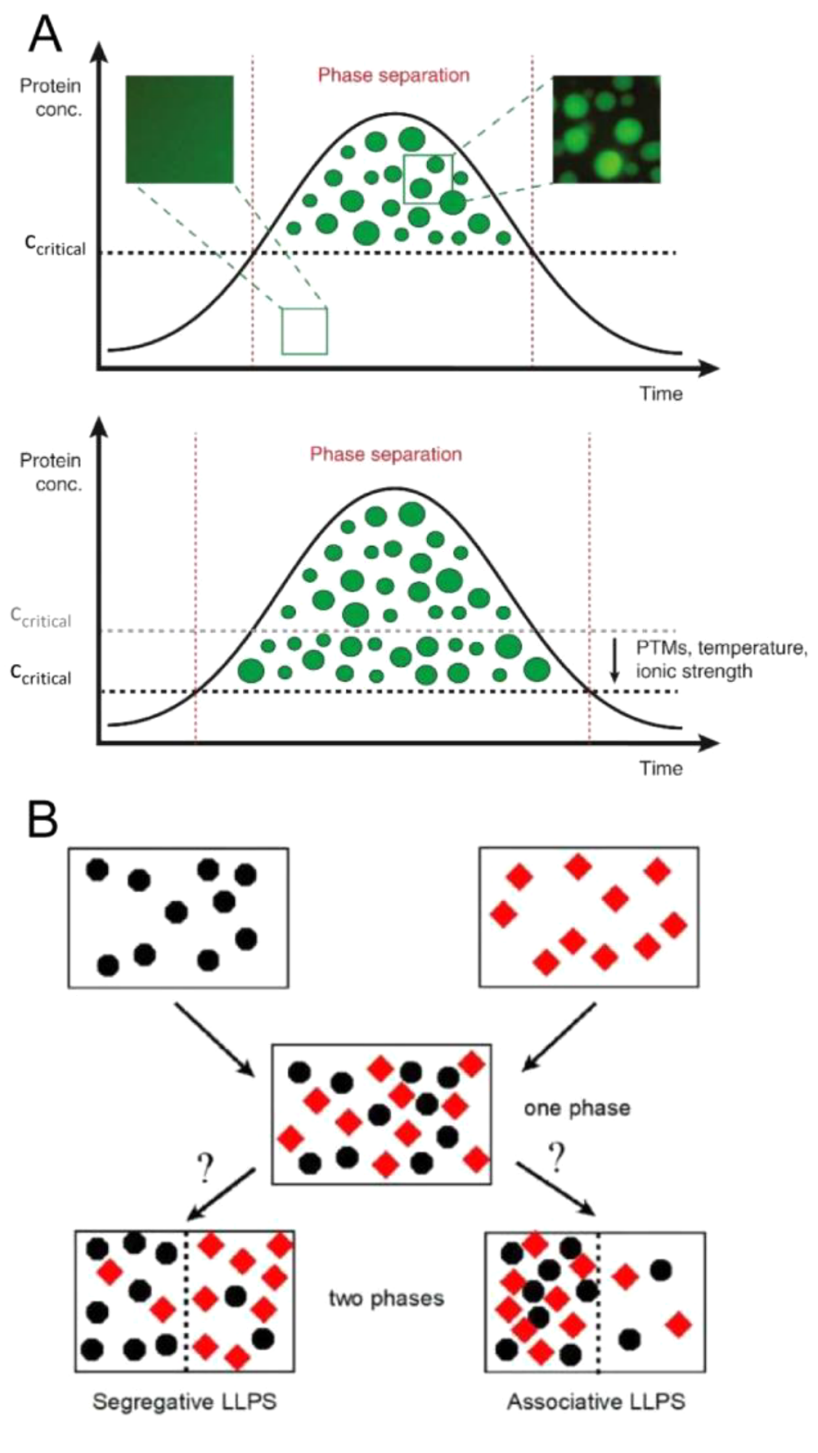
**Phase separation.** (A) Top: A scheme showing the time-dependent formation of liquid droplets of a protein above the critical concentration for phase separation. These protein microcompartments are dynamic and can exchange molecules with the surrounding phase. Below the critical concentration, they dislodge to form a one-phase state. The insets above show original data from a phase separation experiment with purified GFP-tagged FUS (a prion-like RNA-binding protein). Bottom: Post-translational modifications (PTMs) or changes in temperature or ionic strength can lower the critical threshold for phase separation and allow droplet formation at a much lower protein concentration. Reproduced with permission from ref (42). Copyright 2017 Elsevier Ltd. (B) Liquid–liquid phase separation in a solution containing two macromolecular solute species. Black circles denote species 1, and red diamonds denote species 2. Segregative phase transitions occur when the heterointeraction between molecules of species 1 and 2 is more repulsive than self-interactions between molecules of either species 1 or species 2. Associative phase transitions occur when heterointeractions between molecules of species 1 and species 2 are more attractive than self-interactions between molecules of either species 1 or species 2. Reproduced from ref (43). Copyright 2020 American Chemical Society under an ACS AuthorChoice license [https://pubs.acs.org/page/policy/authorchoice_termsofuse.html].

A protein can undergo phase separation and form dynamic droplet-like structures above a critical concentration threshold, which is a function of temperature, pH, ionic strength, and physiologically relevant ligands (e.g., nucleotides) and protein modifications (Figure 2A).^36^ These droplets form a microcompartment that allows diffusion of molecules within the container and promotes the dynamic exchange of molecules with the dilute surrounding phase. The protein-containing droplets are stable above the critical concentration, but the protein system reverts to a one-phase regime when the protein concentration decreases below the critical concentration. Proteins that contain multivalent domains, which are mostly involved in protein–protein and protein–nucleic acid complexes, and those having intrinsically disordered regions are prone to form these droplet-like dynamic structures.^38^ RNA can further promote this process by interacting through RNA-binding domains.^39,40^ Although intrinsically disordered regions in proteins have been traditionally considered the main drivers in the formation of condensates, there is growing evidence that in many instances they have a secondary role, acting as modulators of condensation events promoted by folded domains.^41^

Crowding can promote these phase transition processes (recently reviewed in ref (44)). These studies have revealed two major features.^5,38,43^ If the proteins prone to phase separate establish attractive and nonspecific interactions with each other and with molecular additives such as nucleic acids or crowders, these interactions will lead to the formation of an associative LLPS. This phenomenon is also termed complex coacervation,^45^ in which one phase is enriched in both proteins and molecular additives and the second phase is depleted of both macromolecular species. On the other hand, if the crowders enhance protein associations via volume exclusion, then this nonspecific interaction will lead to a segregative LLPS. In this case, one phase is enriched (relative to the total composition) in the protein and depleted (relative to the total composition) of the crowder, while the second phase is enriched in the crowder and depleted of the protein (Figure 2B).

Significantly, in some instances, such droplet-like structures evolve with time (“age”) to form more solid-like or hydrogel structures, and/or the concentrated molecules within them can form fibrils, etc.^40^ These transitions are mostly related but not restricted to disease states.^46^ These observations have focused on studying the final state of matter resulting from the phase separation process. However, it is compelling to consider LLPS as an active process that may be modulated nonspecifically by crowding and specifically by proteins (i.e., those regulating essential cellular processes), which eventually dynamically act on the membrane (see below). Disentangling these interactions is a challenging task, especially in cellular systems, as phase separation and solubility may cooperate in poorly controlled ways, partly due to the difficulties of measuring precisely the composition dependence of phase diagrams in complex cell-like reconstituted systems and cellular environments.^36,47,48^ These experimental complications lead to ambiguous interpretations of *in vivo* observations related to phase separation and condensate formation.^47^

### 1.3 Interfacial (Surface) Effects

Surface interactions represent a special case of macromolecular partitioning.^4^ A protein near a membrane is in an environment significantly different from one that is distant from the surface.^49,50^ The same applies to the surface of large supramolecular structures such as cytoskeletal fibers. Proteins are localized at the surfaces of these structures by attractive electrostatic and/or hydrophobic interactions in addition to repulsive volume-exclusion interactions.^5,49^ Theory and experiments have shown that adsorbed macromolecules have a stronger tendency to self- or heteroassociate than those in bulk solution and that the tendency to associate increases substantially with the strength of attraction between the soluble macromolecule and the surface.^4,5^ Therefore, surfaces can act as scaffolds for protein organization in which nonspecific attraction between soluble proteins and the surfaces of membranes and fibers leads to enhanced surface adsorption of protein and self- and heteroassociation of adsorbed protein. Interestingly, these interfacial interactions can facilitate the formation of surface-associated assemblies and clusters, some of which could be compatible with phase-separated condensates.^51−53^

Quantitative characterization and correct interpretation of the combined effects of crowding, phase separation, surface interactions, and physicochemical homeostasis on reconstituted systems of increasing complexity will narrow the gap between *in vitro* and *in vivo* studies and provide further insights on the control of cellular functions and the emergent properties of the living cell. Moreover, this approach will aid the building and integration of functional modules from the bottom up in the context of synthetic cell research.^54,55^

## 2 Structure of Bacterial Cytoplasm and Physicochemical Homeostasis

### 2.1 Physicochemical Homeostasis

Physicochemical homeostasis is the ability of a system to maintain steady internal physical and chemical conditions such as (macro)molecular crowding, pH, ionic strength, and turgor pressure. Control of these generic factors is important for the catalytic performance, architecture, and vitality of any cell, regardless of its specific function or ecological habitat. We present the physicochemical homeostasis in connection to the volume regulation of the cell, because osmotic perturbations offer a means to alter and study the physical and chemical state of the cytoplasm. Moreover, osmotic up- or downshifts affect the macromolecular crowding and apparent viscosity, internal pH, ionic strength, and turgor pressure, and it is almost impossible to separate these properties from each other (see extended abstract published in Poolman 2023).^56^ Finally, we connect the physicochemical homeostasis to the energy status of the cell and focus on various interdependencies of these cellular parameters rather than on the mechanisms of the (membrane) proteins involved [for comprehensive reviews on these topics, we refer to refs (57−62)).

#### 2.1.1 Quantitative Aspects of Macromolecular Crowding

In bacteria, proteins make up the majority of the cell’s macromolecules (∼55% w/w) and, together with rRNA (∼15% w/w), are the most space-consuming molecules.^63^ They occupy macromolecular volume fractions (Φ) in the range of 0.13–0.24, depending on the growth conditions.^7,17,64−66^ The excluded volume fraction of the cytoplasm can be even higher when bacteria are exposed to severe hypertonicity, and barriers for diffusion can form due to aggregation of biomolecules.^67^ Intriguingly, in plasmolyzing *E. coli*, the cytoplasm appears as a meshwork allowing the free passage of small molecules while restricting the diffusion of bigger ones. As described in the Introduction, the background interactions (mostly nonspecific) between proteins and other macromolecules and their surroundings within the highly volume occupied bacterial interior can significantly influence the equilibria and rate of macromolecular reactions when compared to the same reactions in uncrowded media.

The high crowding of the cytoplasm speeds up slow (transition-state limited) reactions, allowing processes to occur rapidly and enabling bacteria to grow with doubling times well below 1 h. But there is an optimum to the crowding, because too high an excluded volume (Φ) slows down diffusion-limited reactions.^68^ Computational modeling of a model cell shows that protein synthesis, involving the interaction of large macromolecules (e.g., tRNA and mRNA with ribosomes), is more hindered by high crowding than metabolic pathways involving diffusion of small molecules to the active site of enzymes.^69^ For example, maximal biochemical fluxes for ribosomal systems peak at Φ = ∼0.12, whereas metabolic systems plateau at Φ values from 0.1 to 0.6. The (micro)organisms studied to date have macromolecular volume fractions in the range of 0.15–0.20, which seemingly is the optimum to maximize the overall reactions rates without translational diffusion becoming a limiting factor.

#### 2.1.2 pH Homeostasis

Protons, which participate in biochemical reactions as reactants and/or regulators of enzyme activity, can influence liquid–liquid phase separation and serve as a source of electrochemical energy, known as proton motive force (PMF). The PMF is composed of the membrane potential (ΔΨ, typically negative inside the cell relative to the outside) and the pH gradient (ΔpH, typically inside alkaline relative to the outside). In the equation
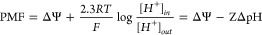
12.3*RT*/*F* equals 58 mV (at *T* = 298 K) and is abbreviated as Z, *F* is the Faraday constant, *R* the gas constant, and *T* is the absolute temperature. The generation of PMF is inseparable from the regulation of the internal pH. Bacteria and archaea generate PMF by electron transfer or respiration, light-driven proton translocation, ATP-driven proton pumps, or coupling of electrogenic transport to a metabolic reaction,^70^ and each of these mechanisms increases the internal pH (the ΔpH component of the PMF). Neutralophiles maintain a roughly neutral cytoplasmic pH (7.0–7.5) when growing in environments at pH 5.5–9.0,^71^ which implies control of proton fluxes. In fact, at alkaline pH the net translocation of protons will be from outside to inside rather than inside to outside because the cytoplasm needs to be acidified. Consequently, the ΔpH is reversed when cells grow at alkaline pH, and the ΔpH makes a larger contribution to the PMF at acidic than at neutral pH; the opposite relationship is observed for the ΔΨ such that the PMF and internal pH of neutralophilic bacteria can be kept relatively constant (see Figure 1b of ref (71)).

Protons are pumped out by respiration or other mechanisms and pumped back into the cell by PMF-consuming processes such as ATP synthesis or nutrient uptake. These processes are not necessarily in balance and prokaryotic cells have additional mechanisms to fine-tune the internal pH, but first we should estimate what is needed for bacteria to maintain a neutral internal pH. A cell like *E. coli* with a radius of 0.4 μm and length of 2.2 μm has a volume of ∼1 fL. At pH 7.2 the number of free protons is only about 10. A few protons entering or leaving such a cell would have a large impact on the internal pH in the absence of intracellular buffering capacity. In reality, a bacterial cell typically has inorganic and organic phosphates in the tens of millimolar range, and in several cases the total phosphate pool is well above 100 mM;^72^ the latter would buffer ∼10 million protons, but additional buffer components can be involved.

Does the internal buffering capacity play an important role in pH homeostasis? The internal buffering capacity has been determined experimentally for a number of Gram-negative and Gram-positive bacteria,^73^ and for *E. coli* it is ∼100 nmol H^•+^·(pH unit·mg of cell protein)^−1^ around neutral pH.^74^ The rate of proton extrusion by respiring *Escherichia coli* cells is 200–1000 nmol H^^•+^^·(min·mg of cell protein)^−1^,^75^ which corresponds to 1 to 5 million H^+^·(s·cell)^−1^. These numbers imply that the internal pH would change by 1 pH unit within seconds if the cell lacked additional mechanisms to compensate for the proton extrusion by the respiratory chain. In addition to passive influx of protons (leakage), the cell translocates protons back into the cell via membrane transport (uptake of nutrients, product excretion, and others), but most of these systems have not evolved to maintain a constant internal pH. For pH homeostasis the cell needs regulatory mechanisms that act fast (high turnover number) and have a specific pH dependence; that is, they are gated by the internal pH.

Cells use different transport mechanisms to simultaneously maintain a relatively constant PMF and internal pH by interconverting ΔΨ and ΔpH. Key regulators of bacterial pH homeostasis are cation/H^+^ antiporters, anion/H^+^ antiporters and metabolite decarboxylation pathways. pH-sensing cation/H^+^ antiporters, acidify the cytoplasm by exporting K^+^ or Na^+^ in exchange for protons when the internal pH gets too high.^71^ One well-studied K^+^/H^+^ antiporter is Kef from *E. coli*.^76,77^ Another well characterized bacterial system is the Na^+^/H^+^ antiporter NhaA from *E. coli*, which has a turnover number of >1000 s^–1^, which exchanges 2H^+^ for 1Na^+^ ions, and whose activity displays a steep pH dependence.^78,79^ The transport by NhaA is electrogenic, implying that it is driven by ΔΨ and chemical gradients of protons and Na^+^ ions. Assuming that a typical *E. coli* cell contains ∼1000 molecules of NhaA, this antiporter alone would allow a respiring cell [translocating 1 to 5 million H^+^·(s·cell)^−1^] to maintain its internal pH within limits. We note that NhaA is driven by ΔΨ, whereas respiration is inhibited by a high ΔΨ. Hence, there is an additional level of regulation (“respiratory control”) of the internal pH beyond pH sensing and gating by the antiporter. Furthermore, a cell typically has multiple ion/H^+^ antiporters, and a large fraction of the protons enters the cell via solute-H^+^ importers for the uptake of nutrients and synthesis of ATP.^70^

**Figure 3:**
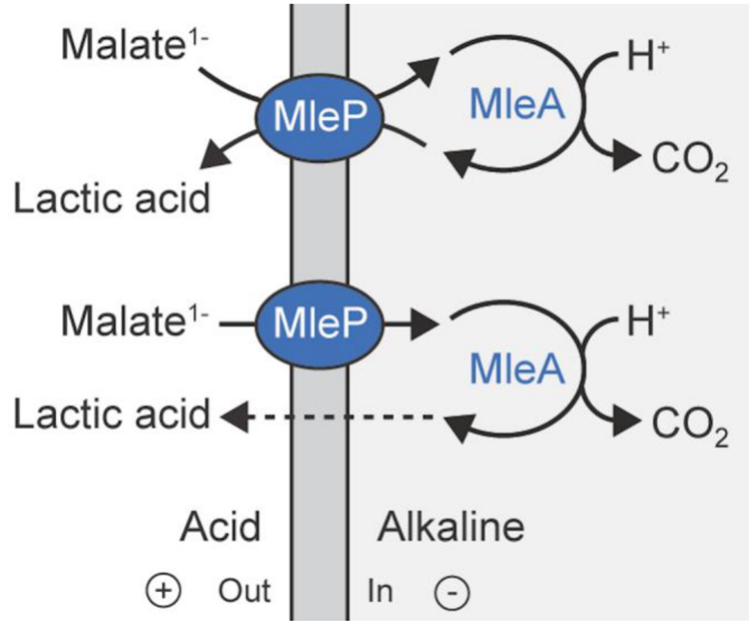
**Decarboxylation of malate by malolactic enzyme MleA, and electrogenic transport of malate via antiport or uniport by MleP.** Passive diffusion of lactic acid across the membrane is shown by the dashed arrow. The energetics of malate^–^/lactic acid antiport and malate^–^ uniport plus lactic acid diffusion are equivalent. Reproduced with permission from ref (70). Copyright 2019 Wiley-VCHVerlag GmbH&Co. KGaA,Weinheim.

pH-sensing ion/H^+^ antiporters acidify the cytoplasm, whereas chloride/H^+^ antiporters (pumping H^+^ out and Cl^–^ in) and metabolite decarboxylation operate during acid stress and alkalinize the bacterial cytoplasm.^70,71,80^ Decarboxylation pathways are found in both respiratory and fermentative bacteria, and they serve to decarboxylate carboxylic acids and amino acids. How do these pathways contribute to pH homeostasis and lead to the generation of a PMF? The chemistry of a decarboxylation reaction requires a proton, and thus, the internal pH is increased (and a ΔpH is formed) when the reaction takes place inside the cell. The substrate and product of the reaction differ in charge because a carboxylate group is removed, but the molecules are otherwise structurally similar. Hence, they can be transported by the same protein, as has been shown for numerous substrate/product antiporters.^70^ The substrate and decarboxylated product carry a different net charge, and thus, a ΔΨ is generated when an antiporter exchanges these molecules.^81−84^Figure 3 shows the case for malate decarboxylation, and here ΔΨ is generated by malate/lactic acid exchange or malate uniport, in addition to passive diffusion of lactic acid across the membrane. In both scenarios, the equivalent of 1 proton is pumped per molecule decarboxylated. Bacterial amino acid decarboxylases have remarkably low pH optima,^85,86^ and their activity increases when the internal pH drops due to enhanced proton influx. Hence, the enzymes have a built-in self-regulatory mechanism to deal with lower pH values and thus contribute to pH homeostasis by pH-dependent decarboxylation.

In summary, the above analysis shows that a relatively high buffering capacity of the cytoplasm is important for absorbing fluctuations in the internal pH, but pH sensing cation/H^+^ antiporters are essential for pH homeostasis under alkaline stress, whereas anion/H^+^ antiporters and metabolite decarboxylation are required under acid stress. Additional levels of regulation can come from the pH dependence of respiration, ATP synthesis/hydrolysis by F_0_F_1_-ATPase, and other processes.^70,71,87^ For longer time scales, pH-dependent regulation of the expression of genes for proton translocating systems can also play a role.

#### 2.1.3 Ionic Strength Homeostasis

The ionic strength of a cell is the effective (and not total) ion concentration of the cytoplasm, expressed in molar units (M). In the equation
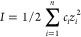
2*i* is the ion identification number, *z* is the charge of the ion, and *c* is the concentration (mol/L) of free ion. The ionic strength screens electrostatic interactions of (macro)molecules and is used to tune enzyme activity and gate membrane functions. The actual ionic strength of the cell is typically not known because a large fraction of the ions is bound to macromolecules. The vast majority of prokaryotes have an overall anionic proteome,^88^ and together with nucleic acids they bind a large fraction of the cations of the cell. The fraction of bound versus free ions is most often not known but can be obtained by comparing the total ion concentration by atomic emission spectrometry with the free ion concentration by specific optical probes. Fluorescence-based sensors have been developed to determine the actual ionic strength inside single cells.^18^ These probes allow observation of spatiotemporal changes in ionic strength in the hundreds of millimolar range and have been used to determine how the internal ionic strength of cells adjusts in response to osmotic challenges.

The ionic strength influences the structure of intrinsically disordered proteins,^89^ the activity of enzymes,^90^ ion channels^91^ and transporters,^92^ protein aggregation,^93^ phase separations,^94^ protein binding to (poly)nucleic acids,^95^ and many other processes. Hence, a given cell maintains its ionic strength within limits, but the actual amounts of ions vary considerably among different species. The most abundant cations in (micro)organisms are K^+^ (∼0.2 M in *E. coli*; ∼20 million K^+^ per cell) and Mg^2+^ (20–40 mM total; 1–2 mM free ion),^63^ but halophiles can also have a high concentration of Na^+^. The reported concentrations of K^+^ in *E. coli*, *Lactococcus lactis*, and the halophilic archaeon *Haloferax volcanii* are ∼0.2, 0.8, and 2.1 M, respectively,^88^ which suggests that across prokaryotes the ionic strength varies more than the internal pH does, but within a species the ionic strength is constrained.

When cells are exposed to an osmotic upshift, the cell volume decreases because water diffuses out. This results in an increase in internal ionic strength and a decrease in internal pH (the proton concentration increases, and a change in ionic strength affects the apparent p*K*_a_ of buffer components). The primary driver of cell volume regulation in *E. coli* and other bacteria upon osmotic upshift is the controlled accumulation of potassium and its counterion glutamate,^73,96,97^ which increases the cell volume but does *not* reduce the increased ionic strength. Excessively high ionic strength can impair enzyme function and be detrimental for the cell. Therefore, in a secondary response to the osmotic upshift, bacteria like *E. coli* and *Bacillus subtilis* replace the K^+^ ions by zwitterionic or neutral compatible solutes such as betaine (*N*-trimethylglycine), proline, and trehalose, thereby maintaining the osmotic pressure and ability to regulate the cytoplasmic volume but reducing the internal ionic strength.^97,98^ The osmoregulatory transporters BetP (*Corynebacterium glutamicum*), ProP (*E. coli*), OpuA (*L. lactis*), and homologues in archaea and bacteria can accumulate high levels of zwitterionic compatible solutes, which increases cell volume and reduces ionic strength.^62,70,92,99,100^ Importantly, these transporters sense ionic strength (or K^+^ ions) and are activated instantaneously when the internal ionic strength reaches a threshold value. Thus, like pH-gated cation/H^+^ antiporters that regulate the internal pH, ionic strength-gated compatible solute transporters regulate cell volume and indirectly influence internal ionic strength and pH.

In general, an ionic strength dependency suggests a role of electrostatic interactions according to the classical electrolyte and double layer theories.^3,101,102^ These theories predict that electrostatic interactions between charged surfaces are screened by a thermal distribution of small ions (ionic cloud), which reduce the range of Coulombic forces as measured by the Debye’s length, usually designated by *1/k*. As activation of osmoregulatory transporters takes place at relatively high ionic strengths (e.g., from 0.2 to 0.5 M), the contribution of the electrostatic force is small. Yet, osmoregulatory transporters such as OpuA are switched from off to on (maximally active state) over this range of ionic strengths, most likely by disrupting multivalent electrostatic interactions between protein residues and an anionic membrane surface^92,103^ (*vide infra*).

#### 2.1.4 Turgor Pressure

Cell turgor (*ΔΠ*) is the hydrostatic pressure difference that balances the difference in internal and external osmolyte concentration. In the equation
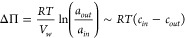
3*V*_*w*_ is the partial molal volume of water, *a* is the water activity, *c* is the total osmolyte concentration, and the subscripts *in* and *out* refer to inside and outside of the cell, respectively. A cell plasmolyzes when *ΔΠ* is zero. Although cell turgor is required for expansion of the cell wall, there is little information on what the lower limit of turgor pressure is before cell growth ceases. Depending upon the species, a bacterial cell may develop up to a few tens of atmospheres of pressure across the cell envelope. Wall-less bacteria such as *Mycoplasma sp.* are not protected against turgor pressure by a peptidoglycan layer, and thus, *ΔΠ* is low.^104^ The turgor pressure in thin-walled Gram-negative bacteria is in the range of 1–3 atm, which amounts to a difference in osmolyte concentration (*c*_*in*_ – *c*_*out*_) of 40–120 mM (∼40 mM/atm). The turgor pressure of thicker-walled Gram-positive bacteria such as *B. subtilis*, *L. lactis*, and *Listeria monocytogenes* can be as high as 20 atm,^67,68,105,106^ corresponding to *c*_*in*_ – *c*_*out*_ of ∼800 mM. Variations in turgor pressure during nutrient shifts in *E. coli* and *Caulobacter crescentus* give rise to elastic changes in surface area, which are thought to be caused by changes in cell width rather than length.^107^ Thus, mechanical forces originating from turgor pressure can regulate the width of bacterial cells and influence macromolecular crowding in the cytoplasm.

Turgor pressure variations are typically much larger when cells are confronted with hypertonic stress (osmotic upshift conditions). In *E. coli* turgor pressure decreases from ∼3 to 1.5 and <0.5 atm when the osmolality of the growth medium is increased from 0.03 to 0.1 and >0.5 Osm.^108^ Although a turgor pressure of <0.5 atm may be sufficient to sustain the growth of *E. coli*, it is possible that Gram-positive bacteria have a higher turgor pressure minimum, because of the potential requirement for higher mechanical (expansion) force acting on the thicker cell wall.^109,110^

**Figure 4:**
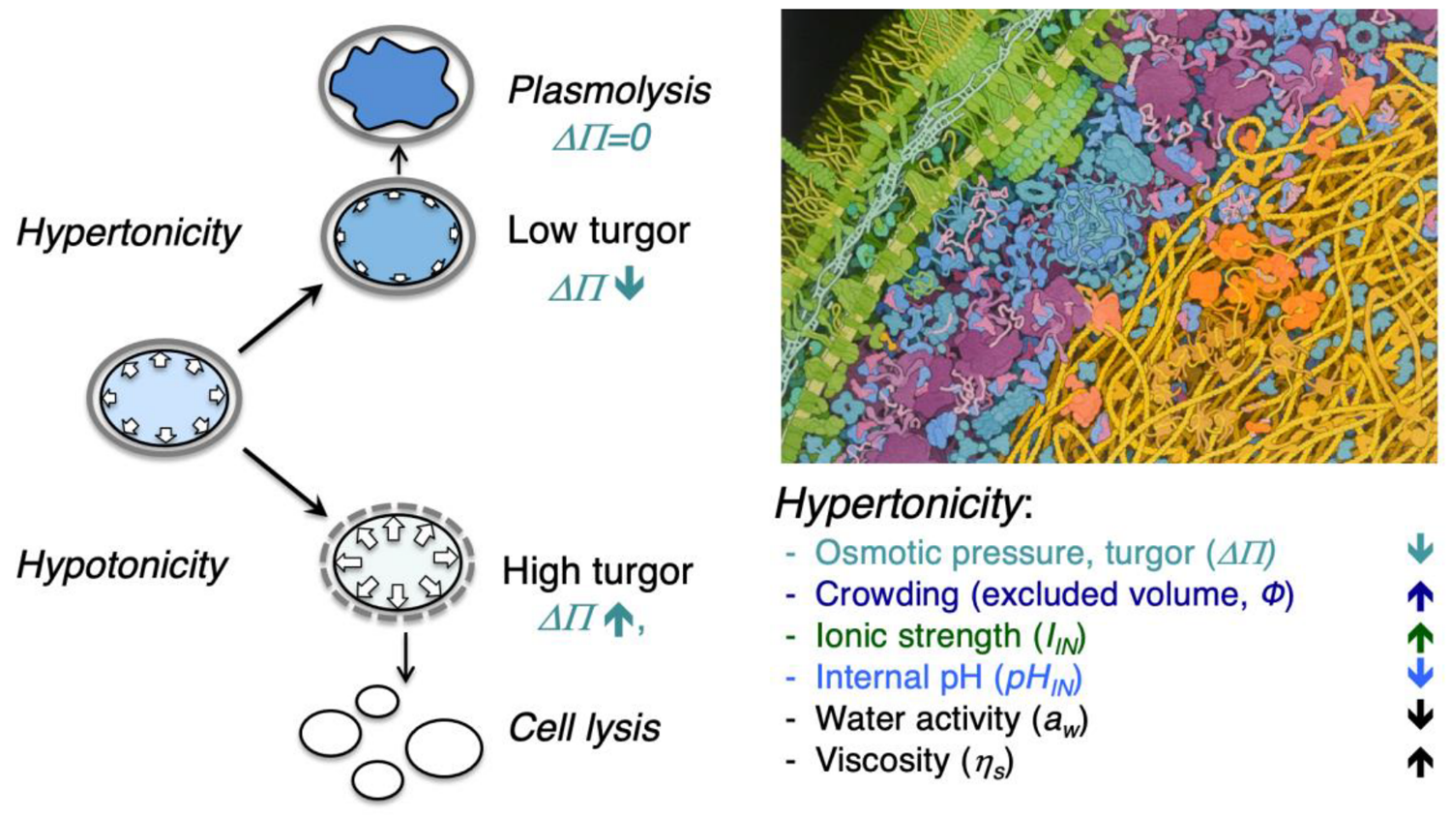
**Osmotic challenges and changes in the physicochemistry of the cell.** Hypertonicity leads to cell shrinkage and a lowering of the turgor pressure (Δπ); cells plasmolyze when Δπ is zero. During plasmolysis, the cell membrane shrinks away from the cell wall, leading to the collapse of the cytoplasm. The effect of hypertonicity on the overall physicochemistry of the cytoplasm is indicated in the bottom right of the figure. Hypotonicity leads to water uptake and swelling of cells, which increases Δπ and ultimately leads to cell lysis. Figure modified from ref (56). Copyright the Author(s) 2023. Published by Oxford University Press under the terms of the Creative Commons Attribution-NonCommercial License [http://creativecommons.org/licenses/by-nc/4.0/]. Top right: Illustration by David S. Goodsell, RCSB Protein Data Bank^114^ depicting the high crowding environment of the bacterial cell, the exclusion of large macromolecular complexes [e.g., (poly)ribosomes in purple] from the nucleoid, and the two-membrane system plus peptidoglycan layer of a Gram-negative bacterium.

Upon a sudden osmotic upshift, turgor pressure and cytoplasmic volume decrease. In addition, the ionic strength, crowding, and (macromolecular) viscosity increase while the internal pH and water activity decrease (Figure 4). Cells counter the detrimental effects of hypertonicity by activating (gating) specific transport proteins that accumulate large amounts of compatible solutes or by synthesis of these molecules,^111−113^ which hydrates the cytoplasm and reverses the physicochemical changes. Various osmoregulatory mechanisms have been described to protect cells against hypertonic stress. Here, we focus on the ATP-binding cassette transporter OpuA of *L. lactis*, to illustrate how a single protein integrates various signals and elicits a response to the stress that encompasses several physicochemical properties.

When the volume of the bacterium decreases and the ionic strength reaches threshold values, OpuA is activated and large amounts of betaine are taken up.^92^ Passive influx of water follows the accumulation of betaine, and consequently, the volume of the cell increases and the ionic strength decreases. The electrostatic gating force acts between a specific osmosensing domain on the protein and the negative membrane plane.^61,70^ Hence, the threshold ionic strength for activation of the transporter can be tuned by varying the fraction of anionic lipids in the membrane.^115^ Macromolecular crowding does not activate OpuA but acts synergistically with ionic strength,^116^ presumably by adversely affecting the electrostatic interactions of differently charged protein–membrane surfaces via excluded volume effects. It was long thought that ionic strength gating was the only mechanism that controlled OpuA activity and the transporter would be switched off after restoration of normal cell volume. The second messenger cyclic-di-AMP has recently been shown to act as a backstop for the protein to prevent rampant accumulation of betaine,^103^ that is, when the volume has been restored but the ionic strength of the stress-adapted cells is still above the gating threshold. Importantly, cyclic-di-AMP also plays a key role in the control of potassium transport, the other key component of cell volume regulation in bacteria.^117−120^

Figure 4 shows that hypotonicity leads to swelling of the cell and an increase in *ΔΠ.* A lipid membrane can stretch up to ∼5% area before lysis tension is reached.^121^ To excrete osmolytes when turgor pressure becomes too high, microorganisms activate mechanosensitive (MS) channels.^59^ Bacteria have different types of MS channels; for example, *E. coli* has seven, but other microbes have a smaller number.^122^ The best-studied MS channels are MscL and MscS, which jettison solutes with little discrimination, except for size, and thereby lower the *ΔΠ* and the risk of cell lysis. The sensing mechanism of these MS channels is completely different from that of the osmoregulatory transporters (*vide supra*). The increase in tension in the membrane following water influx is sensed as a decrease in lateral pressure on the protein, which facilitates the transition from the closed to the open state. The closed-to-open transition of MscL involves an iris-like expansion, which leads to a final open pore diameter of ∼2.8 nm and a conductance of ∼3 nS and requires a gating tension of ∼10 mN/m.^123^ The closed-to-open transition of MscS involves the rotation and tilt of pore-lining helices,^124^ which leads to a final open pore diameter of ∼1 nm and a conductance of ∼1.25 nS and requires a lower gating tension than that for MscL.^59,125^ The MS channels act (gate) on short time scales (∼20 ms),^126^ which is required to counter the rapid swelling upon hypoosmotic shifts. Both MscL and MscS are gated by membrane tension (γ) and the pressure across the membrane (*Δp*) does not play a role as stimulus,^127^ but the two parameters are connected as shown in the Young–Laplace equation:
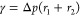
4Here, *r*_1_ and *r*_2_ are the principal radii of the membrane, which change when the cell volume changes.

### 2.2 Structure and Dynamics of Cytoplasm

#### 2.2.1 Macromolecular Composition of Cytoplasm

The bacterial cytoplasm is a complex and dynamic milieu that consists of water, ions, metabolites, macromolecules, and membraneless structures such as the nucleoid (DNA, DNA associated proteins, and RNA), inclusion bodies (irreversible assemblies of macromolecules), biomolecular condensates (reversible assemblies of macromolecules), and membrane-associated cytoskeletal elements. These complex assemblies are universally present in prokaryotes, although well-defined cytoskeletal structures are not found in the simplest bacteria and biomolecular condensates have so far only been studied in a few bacterial species. In addition, various metabolic enzymes across diverse microorganisms form intracellular bodies in the form of fibers and other types of functional mega-assemblies,^128,129^ which can be organism specific. The complex assemblies of macromolecules are mostly segregated from each other (*vide infra*), but they are not compartmentalized via a membrane. A variety of mechanisms underlie the physical separation of the cytoplasmic components, including macromolecular crowding, protein-based scaffolds, liquid–liquid phase separation, and spatial organization via biochemical gradients, but physicochemical factors such as the internal pH and ionic strength also play a role. Subcellular compartmentalization by lipid-based membranes is rare in prokaryotes, but anammoxosomes, magnetosomes, and acidocalcisomes are notable exceptions.^130^ Protein-based nano- and microcompartments are found in bacteria and archaea,^131,132^ and these protein-bounded structures encapsulate dedicated cargo proteins to create a specific environment for enzyme functioning.

In *E. coli* the chromosome and nucleoid-associated proteins localize around the cell center,^133,134^ where they form heterogeneous phase-like structure(s)^135^ that exclude translating ribosomes. These polysomes or polyribosomes (Terminology) localize at the cell poles and cytoplasmic periphery.^133,136,137^ Aggregated or misfolded proteins also localize at the cell poles but typically not evenly between the old and new pole.^138−140^ Single-molecule diffusion measurements with nanoscale resolution have shown that each cell has a so-called slow and fast pole.^141,142^ The slow diffusion at one pole coincides with the old pole of a dividing cell, where aggregated and misfolded proteins are more abundant and most likely hinder the diffusion more than at the newly formed pole.^143^ In terms of the structure of the bacterial cytoplasm, there is increasing evidence for the formation of phase-separated liquid droplets or biomolecular condensates,^144−149^ which are metastable structures where certain proteins partition and others are excluded (see also section 1). The function of biomolecular condensates in bacteria is mostly unexplored territory, but by analogy to mammalian cells they are likely involved in selective recruitment of client proteins, improving the efficiency of enzymatic reactions, and sequestering and processing of RNA and protein molecules, which can help *E. coli* cells resist environmental stresses.^149^ There are only a few studies where condensates in bacteria have been shown to increase the catalytic efficiency by concentrating enzymes and/or its substrate(s). One example is the sequestration and activity of a client kinase upon phase separation by ATP depletion in *C. crescentus*,^150^ showing that ATP depletion can promote LLPS, enforce protein compartmentalization, and sustain enzyme activity. Another example is the activity of a bacterial polynucleotide phosphorylase, which is enhanced when the enzyme colocalizes with RNase E within biomolecular condensates (in this case ribonucleoprotein bodies^151^).

The total of protein and RNA molecules in the cytoplasm of bacteria can reach volume fractions of 15–20% in growing cells and even higher in osmotically stressed cells.^7,14,17,64^ An excluded volume of 20% is equivalent to 3 million globular particles with a radius of 2.5 nm in a volume of 1 fL, which reflects the number and average size of proteins in an *Escherichia coli* cell. If the molecules were evenly distributed, their surface-to-surface distance would be ∼1.9 nm, which is smaller than the radius of the proteins and thus should significantly affect their diffusion.

The macromolecules, ions, and other small molecules of the cytoplasm form a gel-like medium with colloidal properties (Terminology). We postulated two decades ago that macromolecules are not evenly distributed in the cytoplasm and that regions of higher and lower crowding are present; transient networks of electrolyte pathways would wire the cytoplasm, guide the flow of biochemical ions, and increase local diffusivity.^61,101^ The high excluded volume, together with hyperstructures,^152^ metabolons,^153^ intracellular bodies,^128^ and liquid–liquid phase separation,^35,154^ would shape the cytoplasmic structure outside the regions of lower crowding. There is increasing evidence for this view of a dynamic and heterogeneously structured cytoplasm, as we show below.

**Figure 5:**
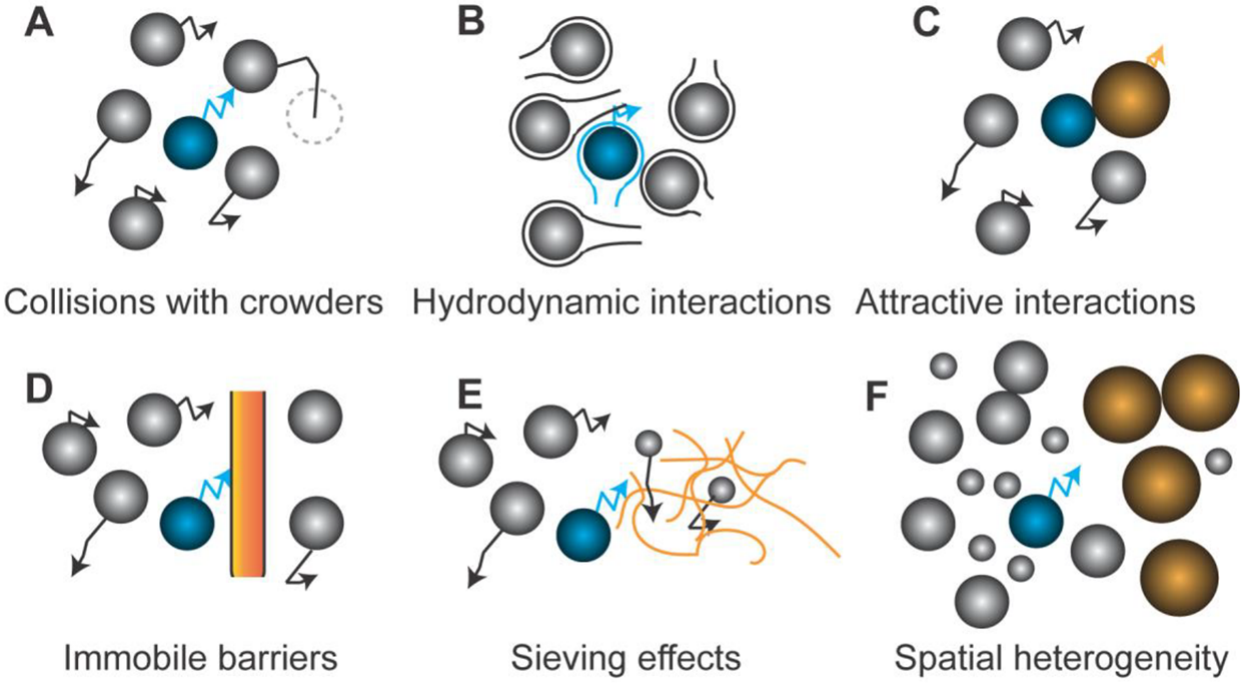
**Factors that affect protein diffusion inside cells.** (A) Hard sphere collisions of the probe (blue) with other freely diffusing molecules (crowders) lowers its diffusion coefficient. (B) Movement through the hydrodynamic wake of another molecule slows down the probe. (C) Complex formation with another particle leads to a lower diffusion coefficient due to the increased effective size of the complex. (D) Immobile barriers such as membranes confine particles in a given part of the cell. The dimensionality of diffusion is reduced at small distances from the barriers. (E) Sieving effects occur when the mesh size of immobile barriers is smaller than the size of the probe, leading to a size-dependent alteration of diffusion. (F) Weak intermolecular forces and steric repulsion between the different biopolymers induce spatial heterogeneity, leading to location-dependent diffusion coefficients of the probe. Reproduced from ref (68). Copyright 2018 Schavemaker, Boersma and Poolman under Creative Commons Attribution License (CC BY) [CC BY 4.0 Deed | Attribution 4.0 International | Creative Commons].

One way to characterize the dynamic structure of the cytoplasm is to determine the mobility or translational diffusion of a molecule. In fact, the translational diffusion coefficient of a molecule inside the cell is frequently used as a proxy of macromolecular crowding under different metabolic or stress conditions. However, the intracellular environment is not a homogeneous medium with a single diffusion coefficient for a given molecule; many factors may retard the diffusion of a protein in a crowded cell, as illustrated in Figure 5. Moreover, the thermodynamic nonideality of the cytoplasm makes the diffusion coefficient not simply a sum of its contributors. Recently developed microscopy and computational methods allow the diffusion coefficient of molecules inside cells to be determined with high spatial and temporal resolution.^141,142^ Below we discuss how these technologies have enabled the characterization of the dynamic structure of the cytoplasm.

#### 2.2.2 Dynamics and Translational Diffusion

Single-particle tracking in bacteria (*E. coli* and *C. crescentus)* and lower eukaryotes (such as *Saccharomyces cerevisiae*) indicates that the cytoplasm is an adaptable fluid that can change from a fluid-like to a more solid-like (“colloidal glassy”) state when cells are deprived of metabolic energy. Pioneering studies by the Jacobs-Wagner lab showed that the *E. coli* cytoplasm acts as a glass-forming fluid in which the diffusion of molecules is disproportionally limited by the size of the tracked component,^155^ which is an example of sieving effects (Figure 5E). Cellular metabolism fluidizes the cytoplasm, which allows larger components to diffuse over larger regions of the cell. When *E. coli* cells are exposed to osmotic (upshift) stress, the cytoplasmic volume decreases and consequently the excluded volume of the macromolecules increases beyond 20%.^67,156^ The decrease in the translational diffusion coefficient of green fluorescent protein (GFP) is proportional to the magnitude of the osmotic up-regulation, and under extreme conditions (≥250 mM NaCl or >500 mM sorbitol in the case of *E. coli*), the excluded volume taken by the macromolecules is so high that diffusion barriers (Figure 5D, mobility barriers) are formed and part of the GFP becomes trapped in discrete pools.^157^

Analogous diffusion studies have been performed in the cytosol of the budding yeast *S. cerevisiae*. Macromolecules are less able to move around in the yeast cytosol when cells are starved of sugar,^158^ which has been attributed to a decrease in cell volume and the accompanying increase in macromolecular crowding. In addition to steric effects, altered physical interactions between macromolecules (Figure 5C), e.g. due to an increase in ionic strength or lower pH at the smaller cytosolic volumes, can also play a role in the translational diffusion of proteins.^159^ In another study,^160^ the more solid-like state of the cytosol of energy-starved cells is attributed to acidification of the cytoplasm, which leads to widespread assembly of macromolecules and thereby a reduced diffusion of large particles. Munder and colleagues conclude that acidification and osmotic stress result in different states of the cytoplasm, and thus, the underlying mechanism of reduced diffusion may differ.^160^ Altogether, these and other studies^161−164^ in prokaryotes and eukaryotes show that metabolic activity directly or indirectly affects the apparent viscosity and structural organization of the cytoplasm. Indirect metabolic effects may include stress conditions that affect the stability of the proteome.^165^ If a fraction of proteins or protein domains unfold as a result of, e.g., heat stress, these denatured polypeptides may exhibit properties akin to those of intrinsically disordered proteins and increase the (local) viscosity. In a recent study,^166^ Di Bari et al. show that the unfolding of just a small fraction of proteins can cause a slowdown of protein diffusivity by forming an entangling interprotein network across the cytoplasm, which is dominated by hydrophobic interactions.

The recently developed technique of single-molecule displacement mapping has been used to resolve the dynamics of a wide range of selected target proteins differing in mass, oligomeric state, abundance, and number of interaction partners (expressed as loneliness factor) with nanoscale resolution,^141,142^ which has provided new insight into the dynamic structure of the bacterial cytoplasm. It was shown that the translational diffusion coefficient (*D*) of proteins in *E. coli* scales with the complex molecular mass, that is, the mass of the tagged polypeptide chain multiplied by the oligomeric state, and not with their abundance in the cell or their loneliness factor.^141^ Furthermore, the diffusion in the *E. coli* cytoplasm does not follow the Einstein–Stokes equation:^167^
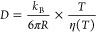
5

**Figure 6:**
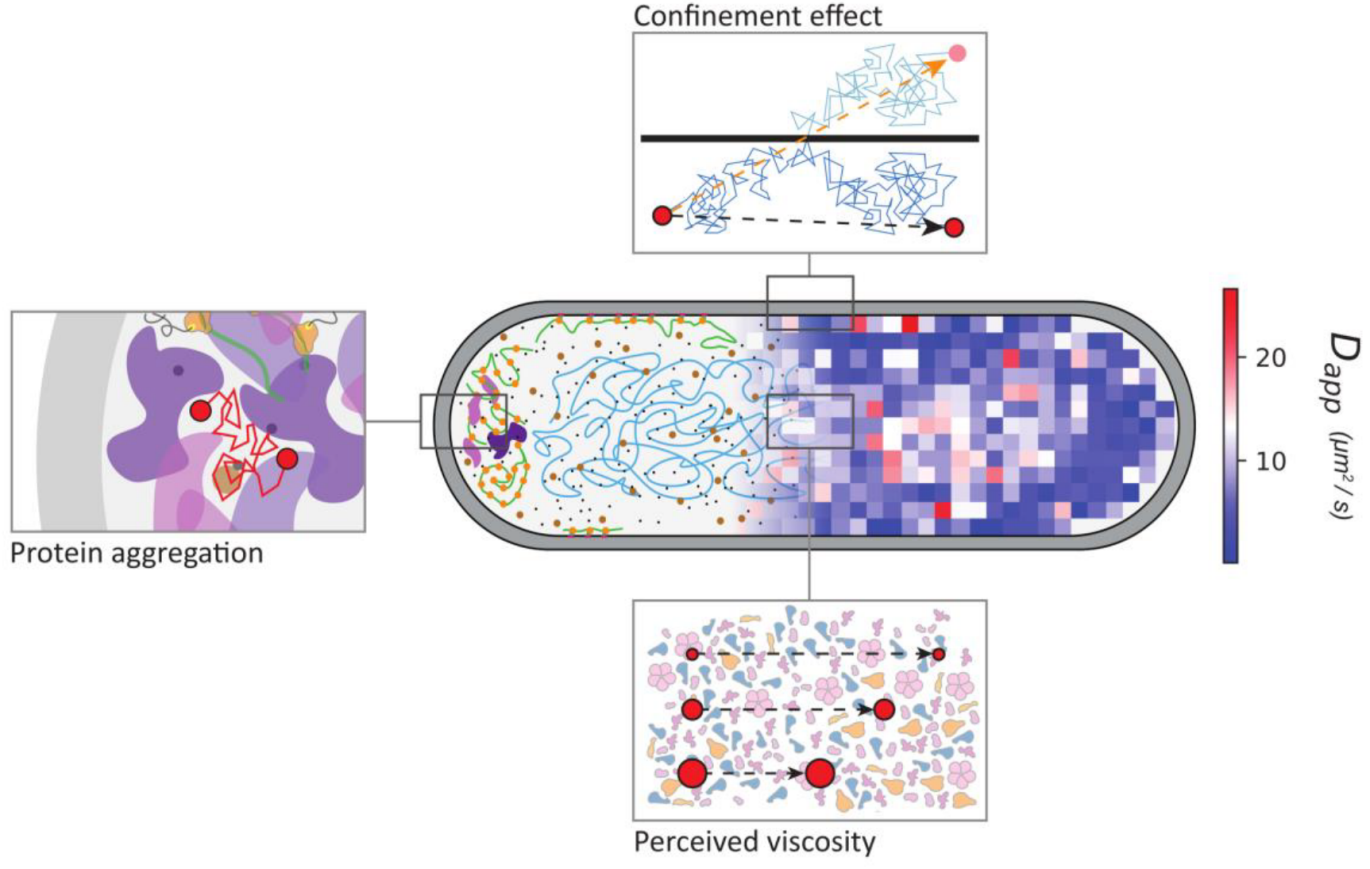
**Structure of*****Escherichia coli*****cytoplasm and impact of confinement, protein aggregation, and perceived viscosity on the translational diffusion of proteins (red particles).** The image in the middle shows a diffusion map obtained by single-molecule displacement mapping (right), a method to determine the mobility of (macro)molecules,^141,142^ which is overlaid with a schematic of the cytoplasm. The figure emphasizes three factors that affect the translational diffusion of molecules: (i) confinement; (ii) aggregation of macromolecules at the cell poles; and (iii) perceived viscosity. Since diffusion of proteins scales with their complex mass, bigger particles will be affected more by the crowding of the cytoplasm than smaller molecules (hence they perceive a different viscosity) and move relatively more slowly, leading to the deviation from the Einstein–Stokes equation. *Dapp* = apparent diffusion coefficient of molecules; the pixel size indicates the spatial resolution at which the diffusion of molecules in the cell can be determined. Reproduced from ref (143). Copyright 2023 Mantovanelli et al. under the terms of the Creative Commons Attribution License [https://creativecommons.org/licenses/by/4.0/].

The dependence of the diffusion coefficient on the complex mass of proteins follows a power law relationship *D* = α*M*^β^, where *M* is the complex mass and α and β are fitting parameters. The exponent β would be −0.33 in the Einstein–Stokes equation, assuming the proteins are globular and not interacting with each other. A value of β = −0.6 has been found for the diffusion of proteins in the cytoplasm of *E. coli*.^141,143^ The stronger than predicted dependence on molecular mass reflects the high macromolecular crowding of the cytoplasm and the collisions with other macromolecules, for which the term “macromolecular viscosity” has been introduced. The deviation of the diffusion coefficients from the Einstein–Stokes equation is explained by the proposal that the cytoplasm is a dilatant, non-Newtonian fluid. A characteristic of dilatant fluids is that viscosity increases with stress applied to the fluid. Larger components inside the cell impose a higher pressure on the environment, which in response becomes more viscous. In this view, the viscosity of the cytoplasm is considered as a function of the analyzed macromolecule, which will be subjected to a perceived viscosity depending on its size (Figure 6). This has led to a modified version of the Einstein–Stokes equation (eq 6):
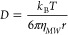
6where *η*_*MW*_ represents the perceived viscosity as a function of the molecular weight. The perceived macromolecular viscosity varies from 9.9 cP to 18.1 cP for protein ranging in mass from 26 kDa to 318.9 kDa.

Similar observations of size-dependence of diffusion were made in a recent study, employing fluorescence correlation spectroscopy and computer simulations. Here, it was concluded that the size-dependence of diffusion is consistent with eq 5 when the specific dumbbell shape of the protein fusions is taken into account.^168^ Furthermore, pioneering studies on protein diffusion in *E. coli* have been made by ensemble measurements, using fluorescence recovery after photobleaching (FRAP),^67,156,157,169−171^ reviewed by Mika and Poolman.^172^ Although the ensemble measurements provide less detail and spatial resolution than single-molecule analyses such as single-molecule displacement mapping, the data are in agreement with the notion that the bacterial cytoplasm behaves as a non-Newtonian dilatant fluid and has macromolecular viscosity that is a function of the probe size. Finally, the diffusion of proteins in the mass range of 26–319 kDa is in agreement with the apparent average mesh size of ∼50 nm of the *E. coli* chromosome.^173^ Thus, the tested proteins with a Stokes radius up to 5 nm may not be affected by the meshwork of the chromosomal DNA.

Importantly, the translational diffusion of the selected proteins is location-dependent in *E. coli*, with the cell poles displaying slower diffusion throughout the whole set of investigated proteins and one pole showing faster diffusion than the other.^141,143^ The extent of the slowdown in the pole regions exceeds the confining effects of the cell membrane boundary, as inferred from computer simulations, and instead is most likely a consequence of hindrance by large macromolecular complexes due to accumulation of damaged proteins primarily at the old cell pole (Figure 6).^143^ Preliminary experiments on protein diffusion in the Gram-positive pathogen *L. monocytogenes* point toward a similar location-dependent mobility.^105,174^ It still is an outstanding question whether symmetrically dividing unicellular microorganisms age.^175^ The differences in diffusion coefficients and protein probe concentrations between old and new cell poles suggest that exclusion of aggregates and other supramolecular complexes from the nucleoid leads to bacterial aging.^139^ The selective segregation of aggregates to the old cell pole may maintain the viability of the whole population.

In general the diffusion coefficients for proteins like GFP are similar across bacterial species,^68^ which points toward similar levels of macromolecular crowding. Furthermore, both the Gram-negative bacterium *E. coli* and the Gram-positive bacterium *L. lactis* respond to osmotic stress by a drop in protein diffusion, which is mitigated when the medium contains osmoprotectants (Terminology). For both organisms a drop in cell size and diffusion coefficient happens even after a small osmotic upshift (0.1–0.2 Osm).^176^ This suggests that the cell wall, which is initially stretched, causes the cytoplasm to shrink when the turgor pressure is decreased (see also ref (109)). There are also important differences between the two organisms. *L. lactis* is less susceptible to osmotic challenge than *E. coli*, as it requires higher medium osmolalities to decrease the diffusion, which most likely relates to the order of magnitude higher turgor pressure of *L. lactis* relative to that of *E. coli*.^176^ An even more striking difference is that in *L. lactis* the GFP diffusion coefficient drops much more rapidly with volume than in *E. coli*. This suggests a different adaptability of the cytoplasmic fluid, but the underlying cause is unknown.

#### 2.2.3 Diffusion-Limited Reactions and Surface Properties of (Macro)molecules

How common are diffusion-limited reactions in the cytoplasm of prokaryotic cells? Schavemaker et al.^68^ reviewed cases where protein diffusion plays a determining role in the physiology and biochemical organization of the cell. Reactions are diffusion limited when the association rate constant (*k*_*on*_) depends only on the translational diffusion coefficient. The *k*_*on,*diffusion_ of a protein diffusing in the cytoplasm with *D* = 10 μm^2^/s and needing to interact with another molecule is ∼10^8^ M^–1^ s^–1^. As most proteins are not reactive over their entire surface, a more realistic diffusion-limited *k*_*on*_ is in the range of 10^5^–10^6^ M^–1^ s^–1^.^68^ Here the assumption is that only a fraction of the surface (the interaction interface) of a molecule is reactive and the interaction between two molecules is not steered through specific (oppositely charged) surfaces. Protein pairs such as Barnase–Barstar from *Bacillus amyloliquefaciens* manage to have a *k*_*on*_ of 10^8^–10^10^ M^–1^ s^–1^ and apparently behave beyond the diffusion limit. The interaction of Barnase (cationic, p*I* ∼9.2) with Barstar (anionic, p*I* ∼4.9) is driven by electrostatic attraction,^177−179^ which allows the *k*_*on*_ for the binding of the ribonuclease to the inhibitor protein to be orders of magnitude higher than the nonelectrostatic diffusion limit. For such interactions, the magnitude of the diffusion coefficient is crucial, with the initial interaction of the proteins likely to be the slowest step. Other diffusion-limited reactions in prokaryotes can include enzymes with very high *k*_*on*_ values,^68^ the ternary complex of amino acyl-tRNA, EF-TU plus GTP finding the ribosome,^180,181^ proteins present in the cell at low copy numbers (longer distances to cover), and proteins transiently binding to membranes or other large structures (e.g., the Min oscillation system^182^).

**Figure 7:**
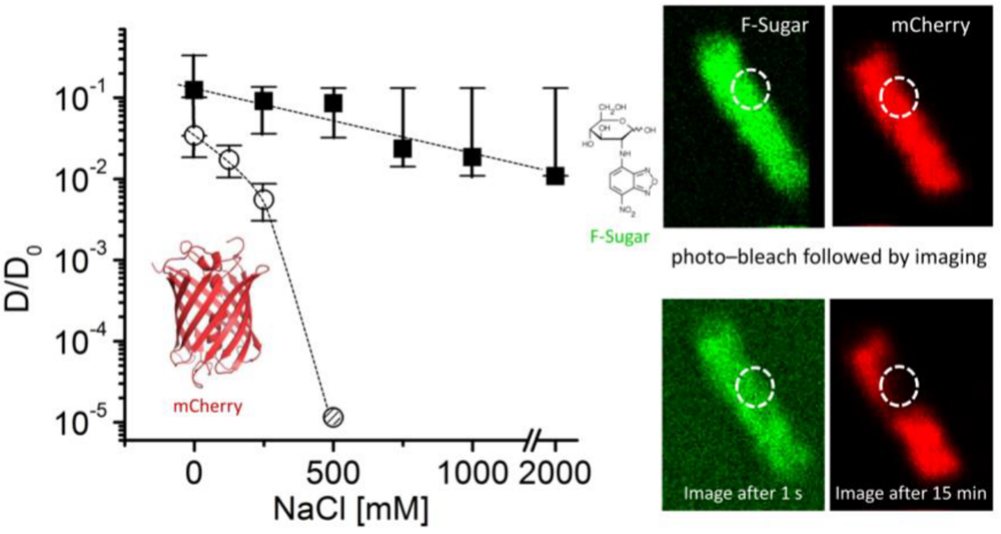
**Effect of osmotic upshift (NaCl stress) on the diffusion coefficient of the red fluorescent protein mPlum and NBD-glucose (FSugar).** The D values are normalized relative to the diffusion coefficients in the absence of NaCl (*D*_0_); data taken from ref (67). Copyright 2010 Blackwell Publishing Ltd. The images on the right show a photobleaching experiment of *E. coli* cells untreated (left) or upshifted with 500 mM NaCl (right).

Most of the processes in the cell are most likely reaction rather than diffusion limited, despite the high crowding in the bacterial cytoplasm. This changes when cells are exposed to osmotic upshift and the crowding increases further. Consequently, the diffusion coefficient of macromolecules decreases by orders of magnitude (Figure 7) and many reactions will become diffusion limited. In extreme cases, diffusion barriers (Figure 5D) are formed and molecules are trapped in supramolecular aggregates.^157^ Remarkably, under conditions where proteins are trapped, small molecules like fluorescent sugars (NBD-glucose in Figure 7) are little affected by osmotic upshifts and can readily diffuse throughout the entire cytoplasmic volume even at 1 M or higher concentrations of NaCl stress (Figure 7). These data indicate that the cytoplasm acts as a molecular sieve (Figure 5E) during both high and low osmotic stress but with a different mesh size. The remarkable diffusion of NBD-glucose in plasmolyzed cells is also consistent with the notion of electrolyte pathways wiring the cytoplasm.^101^ The rapid diffusion of small molecules (ions, metabolites, signaling molecules) may keep the cell biochemically active, even when the majority of enzymes are trapped. This may allow the cell to recover from extreme osmotic stress, provided it can take up or synthesize compatible solutes.

The cytoplasm consists of various types of nucleic acids and >1000 types of protein, but only 50 protein types make up 85% of the cytoplasmic proteome of *E. coli*.^183^ These abundant proteins have a large impact on the structure of the cytoplasm through, e.g., weak and nonspecific interactions with other molecules (https://www.ebi.ac.uk/intact/home). However, analysis of protein diffusion as a function of loneliness factor in the *E. coli* cell does not reveal a correlation between a protein’s diffusion coefficient and the number of interaction partners.^141^ The boundary conditions for the importance of generic nonspecific interactions (Figure 5C) between macromolecules have been probed in a study of diffusion of surface-modified fluorescent proteins. The diffusivity of a set of GFP variants with a net charge ranging from −30 to +25 has been analyzed in *E. coli* (Gram-negative bacterium), *L. lactis* (Gram-positive bacterium), and *H. volcanii* (archaeon).^88^ These three organisms differ in their cytoplasmic ionic strength, as shown by measurements on the K^+^ ion concentrations, which, as mentioned above, are ∼0.2, 0.8, and 2.1 M, respectively. In *E. coli* the diffusion coefficient of GFP variants depends on the net charge and its distribution over the surface of the protein, with cationic proteins diffusing up to 100-fold slower than anionic ones. The decrease in GFP mobility is due to the binding of cationic GFP to ribosomes. This effect is weaker in *L. lactis* and *H. volcanii* due to electrostatic screening. Interestingly, the number of cationic proteins in *E. coli* with a net charge >+10 (surface charge comparable to that of the slowed cationic GFPs) is only 35, of which 18 are ribosomal proteins, 14 are DNA/RNA associated, and 3 have unknown functions. The same holds true for the vast majority of (micro)organisms, with endosymbionts of plants and insects being notable exceptions.^88^ Protein–protein interaction pairs such as cationic Barnase and anionic Barstar are rare in bacteria. Thus, the proteome of bacteria is generally anionic and appears to have evolved by avoiding highly cationic surfaces; the cationic proteins would lower the overall diffusivity and might affect the functioning of the ribosomes. The highly cationic proteomes of some endosymbionts indicate that these organisms have special mechanisms to avoid slow diffusion and perturbation of ribosomal function.

#### 2.2.4 Nucleoid Structure

The bacterial nucleoid excludes ribosomes and some other proteins (see section 2.2.1), suggesting that it acts like a molecular sieve. As mentioned above, translating ribosomes (polysomes) are excluded from the nucleoid and localize mostly at the cell poles and cytoplasmic periphery.^173^ Nevertheless, ribosomal subunits with Stokes radii in the range of 15–20 nm can penetrate the DNA meshwork of the nucleoid as shown in *E. coli* and other bacteria.^137,184,185^ This also holds for metabolic enzymes with Stokes radii in the range of 5 nm.^141^ When the molecule size is close to the average mesh size of the nucleoid, the diffusivity of the particle becomes limited but smaller molecules diffuse freely through the meshwork (Figure 5E).

What are the molecular sieving properties of the nucleoid and what biophysical properties of the cytoplasm are important for its structure? The apparent mesh size of the *E. coli* chromosome is around 50 nm.^173^ Obviously, the volume of the cell, confinement by the cell membrane, ionic strength (polyvalent cations in particular), and macromolecular crowding play key roles in the structure and phase properties of the nucleoid, in addition to specific proteins associating with the DNA. The high excluded volume of the cytoplasm causes repulsion between macromolecules, which results in a compacting force through steric effects.^33^ This can lead to condensation of DNA, nucleoid size reduction, and DNA segregation,^186^ which can be antagonized by DNA-associated proteins.^187^ Furthermore, the overall quality of the cytoplasm as solvent will play a role. In polymer chemistry the quality of a solvent is classified as good when it exhibits a high degree of solubility and compatibility with a polymer, that is, if it allows the polymer to be well dissolved and dispersed. A poor solvent has limited solubility or affinity for a polymer and can induce phase separation in polymer solutions. Using the mesh size of the nucleoid and DNA concentration in the cell, Xiang and colleagues^173^ concluded that the cytoplasm behaves as a poor solvent for the chromosome. Computer simulations show that the poor solvent leads to chromosome compaction and domain formation. RNAs may contribute to the poor solvent effects, which would connect chromosome compaction and domain formation to transcription.

The volume of growing *E. coli* cells is ∼1 μm^3^, and the average volume of the nucleoid with one chromosome (∼4.6 × 10^6^ base pairs) is estimated to be ∼0.7 μm^3^.^173^ From these numbers one can calculate the average DNA concentration in the nucleoid region of around 7 mg/mL,^173^ but 10-fold higher concentrations have also been reported (see footnote 6 in Murphy and Zimmerman^188^). In the older studies, cells appear larger and the nucleoid occupies a smaller fraction of the cytoplasmic volume. Interestingly, when the genome size of bacteria is plotted against cell volume^189^ there is an enormous variation in the amount of DNA per unit of cell volume. For instance, the tiny *Bdellovibrio bacteriovorus*(190) accommodates a chromosome of ∼3.8 × 10^6^ base pairs in a volume that is more than 10 times smaller than the *E. coli* cytoplasm. Thus, irrespective of the volume of the nucleoid region, the DNA must be compacted even more than in *E. coli*. Similarly, other small bacteria such as *Haemophilus influenzae*, *Mycoplasma* sp., and *Pelagibacter* sp. have much more DNA per unit of volume than *E. coli* (see supplement of Bailoni and colleagues^189^). The more compacted DNA will result in a smaller mesh size of the corresponding chromosome, which may affect the exclusion of proteins from the nucleoid and the distribution of macromolecules inside these cells, and possibly their aging. Indeed, the chromosome of *B. bacteriovorus* is highly compacted in a polarized nucleoid that excludes freely diffusing proteins during the nonproliferative stage of the cell cycle.^191^

#### 2.2.5 Fluidization of the Cytoplasm

What causes the fluidization of the cytoplasm by metabolism? Both in *E. coli* and the lower eukaryote *S. cerevisae*, depletion of metabolic energy reduces mobility of proteins, which has been attributed to a lowering of the ATP pool,^155^ a lowering of the internal pH,^192^ and an increase in macromolecular crowding.^158^ The mechanistic basis for the fluidization of the cytoplasm is complex, as ATP levels, internal pH, and crowding are connected and each of these physicochemical parameters can affect molecular interactions (e.g., protein aggregation) but also chromosome compaction. Multiple antibiotics studies have shown that changes in nucleoid compactness influence the diffusivity of molecules.^193−196^ Furthermore, when an enzyme undergoes large conformational changes in its catalytic cycle, it induces hydrodynamic flows in the surrounding fluid or membrane.^197^ Such pulsating flows can act on any passive particles in the solution or lipid bilayer. The collective hydrodynamic effects of active macromolecules can increase diffusion of all particles in the medium and in special cases result in directed flows. The collective conformational changes of enzymes and other macromolecules will be higher when cells are in a metabolically active state than when metabolic activity is low.

How could a change in ATP concentration by itself affect protein mobility? ATP has been postulated to act as a biological hydrotrope^198^ (Terminology). A hydrotrope is capable of solubilizing (hydrophobic) substances in an aqueous solution without the need for micelle formation. ATP and GTP at physiological millimolar concentrations have been shown, at least *in vitro*, to have hydrotropic properties and keep proteins soluble and minimize their aggregation,^199,200^ which may keep the cytoplasm more fluid. NMR spectroscopy has shown that ATP interacts weakly with various proteins, which may provide protection to protein surfaces.^201^ It has also been postulated that the dynamics of enzymes catalyzing metabolic reactions can have a “stirring” role in the cytoplasm.^163^ Many enzymes are ATP or GTP dependent, and depletion of these nucleotides will reduce the conformational dynamics of these proteins, which indirectly may affect other enzymes. There is debate whether or not enzymes at work (irrespective of ATP) are able to self-propel or to break free from supramolecular structures,^202−204^ which would also have a fluidizing effect. Recent studies on the diffusion of single molecules do not show catalysis-induced diffusion of alkaline phosphatase and urge a revisit of previous findings and models.^205^ However, there is increasing evidence that enzymatic activity generates a microflow in the surrounding medium,^206^ which will impact the diffusivity and dynamic structure of the cytoplasm.

## 3 The Bacterial Nucleoid

**Figure 8:**
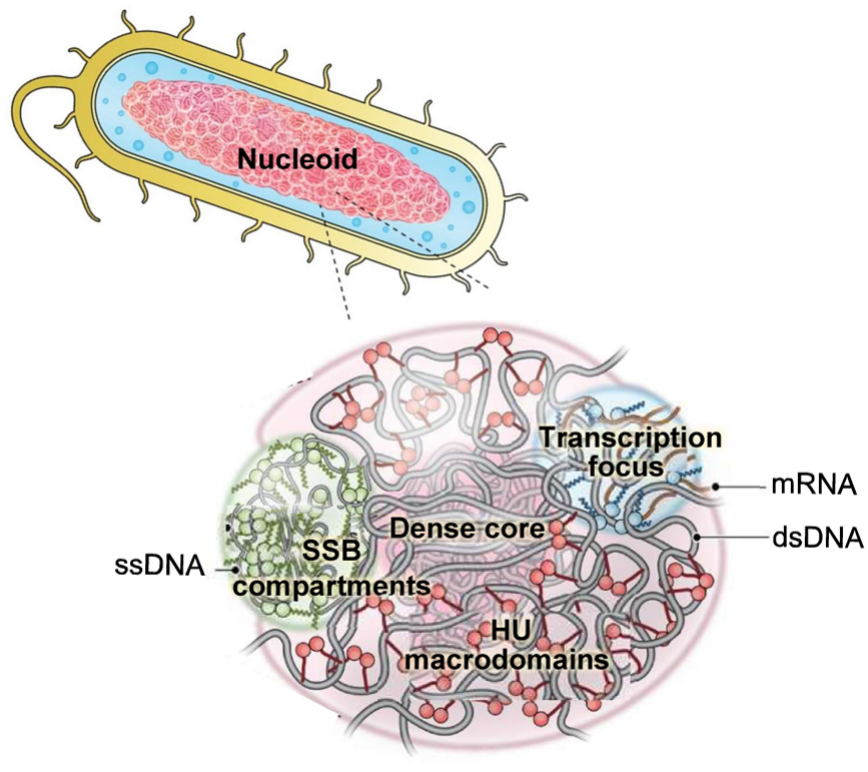
**The bacterial genome is organized as a phase-separated nucleoid.** HU is a histone-like protein that packages DNA into a dense core surrounded by a less dense phase of DNA and associated proteins. Transcriptional foci are dynamic condensates comprised of RNA polymerase and other transcription factors. The single-stranded DNA binding protein (SSB) also forms compartments. Abbreviations: dsDNA, double-stranded DNA; ssDNA, single-stranded DNA. Figure adapted and modified with permission from ref (221). Copyright 2021 Elsevier Ltd.

The bacterial nucleoid, with its several megabases of chromosomal DNA, is remarkably confined and compact despite the lack of a dedicated membrane to enclose it.^207,208^ Initially described as a collection of loops emanating from a dense core organized by proteins and RNA,^209,210^ the nucleoid has since been revealed to be a condensed phase (Figure 8) formed by LLPS through the interaction of multivalent cations and proteins in the presence of crowding agents.^211−213^ Mobility within this dynamic structure allows organization of the chromosomal loci as required during the cell cycle.^214^ Interestingly, its size and positioning within the cell are regulated by crowding and cell geometry.^134,215^ Atomic force microscopy and simulations with varying DNA concentrations show that self-crowding modifies nucleoid shape and properties depending on supercoiling density, which is essential for DNA replication.^216^ Nucleoid size also changes in response to antibiotics.^217,218^ For example, inhibition of translation with chloramphenicol results in ultracompaction of the nucleoid, presumably because of the loss of coupled translation with membrane insertion of proteins (transertion).^193,219^ Although most bacteria have a single, circular chromosome, in a few cases the genetic material distributes in two or more chromosomes.^220^

The role of phase separation in the organization of DNA-based structures and regulation of protein-nucleic acid complexes in different organisms, including bacteria, has been comprehensively reviewed recently.^221^ Quantitative simulations propose that nucleoids are assembled and organized by segregative phase separation, probably as a first level of compaction, as a result of demixing of the chromosome and the macromolecules within the cytoplasm. These simulations show that different geometries of molecular crowders result in different repulsive interactions important for nucleoid organization.^222^ By analogy to the mitochondrial genome in eukaryotic cells, the bacterial chromosome is further organized by nucleoid associated proteins (NAPs), which bind to DNA with little sequence specificity,^207^ in contrast to mammalian nuclear genomes that assemble into orderly spaced nucleosomes. It is worth noting that the highly crowded conditions within the nucleoid result from the high density of NAPs that coat the chromosome (ca. 30% of the chromosome in *E. coli*), limiting its available protein-free regions.^223^ In fact, a phenomenological model of cytoplasm length-scale-dependent viscosity that considers crowding, including NAPs on DNA, shows that it alters the nonspecific binding of transcription factors and their 1D diffusion along DNA in *E. coli*.^224^ Some of the NAPs exhibit phase separation behavior, including the histone-like heat-unstable nucleoid protein (HU, see also section 7), a DNA-binding protein from starved cells (Dps, see also section 7), single-stranded DNA binding protein (SSB, see sections 6.1 and 7), and RNA polymerase (RNAP).

HU is one of the most abundant NAPs, and this protein is conserved across all bacteria.^225^ Upon interaction with MukB, HU ensures proper positioning of the chromosomal replication origin *oriC* in *E. coli.*(220) Two isoforms, HU-A and HU-B, contain intrinsically disordered regions and domains for homo- and heterodimerization. *In vitro*, these proteins form coacervates with DNA, causing phase separation, favored by PEG as crowding agent.^226^ Using fluorescently labeled HU, multiple dynamic submicron-sized condensates have been observed in *E. coli* cells that rearrange, probably through separation and fusion, over a time scale of a few tens of seconds. DNA and protein concentration, increasing temperature, and lower pH and salt concentrations are among the factors that enhance the condensation of HU-B. HU-A also assembles into homotypic and heterotypic condensates with HU-B, although it is less prone to coacervation with DNA than HU-B. This is consistent with the prevalence of HU-A mainly as dimers and discrete complexes with DNA, whereas HU-B self-associates into dimers, tetramers, and octamers and forms multiple higher order complexes with DNA, emphasizing the importance of weak multivalent interactions for condensation. HU condensates recruit a variety of nucleic acids, and phase separated HU-DNA droplets colocalize with DNA polymerase *in vitro*.

HU proteins also form heterotypic condensates with Dps,^226^ a NAP that contains disordered regions and assembles into dodecamers *in vitro*. In the presence of DNA, Dps demixes into condensates of smaller size compared to those of HU. Despite being dynamic and hence liquid-like, Dps condensates display a mixture of round and irregular shapes, compatible with a lower tendency to fuse. The distinct properties of HU and Dps condensates may be due to differences in interfacial surface tensions or shear relaxation characteristics. When assembled in the presence of DNA, condensates involving the two proteins consist of multiple droplets of Dps encircled by a larger droplet of HU-A or HU-B, a remarkable behavior probably arising from the different properties of HU and Dps condensates. Moreover, this arrangement seems to be dependent on DNA binding by Dps, as crowding-driven homogeneous condensates, in which both proteins fully colocalize, are obtained in the absence of nucleic acids.

Dissimilarities in the condensation features of the two HU isoforms and Dps suggest an interesting mechanism to spatiotemporally tune the level of phase separation through the HU-A:HU-B:Dps ratio. For example, during the early logarithmic growth phase, accumulation of HU-A would decrease phase separation by DNA in nucleoids to allow constant replication and gene expression. In contrast, higher levels of HU-B in the late logarithmic phase and of Dps in the stationary phase or during starvation^227^ would favor phase separation, promoting DNA compaction and providing resistance to stress (see section 7).

**Figure 9:**
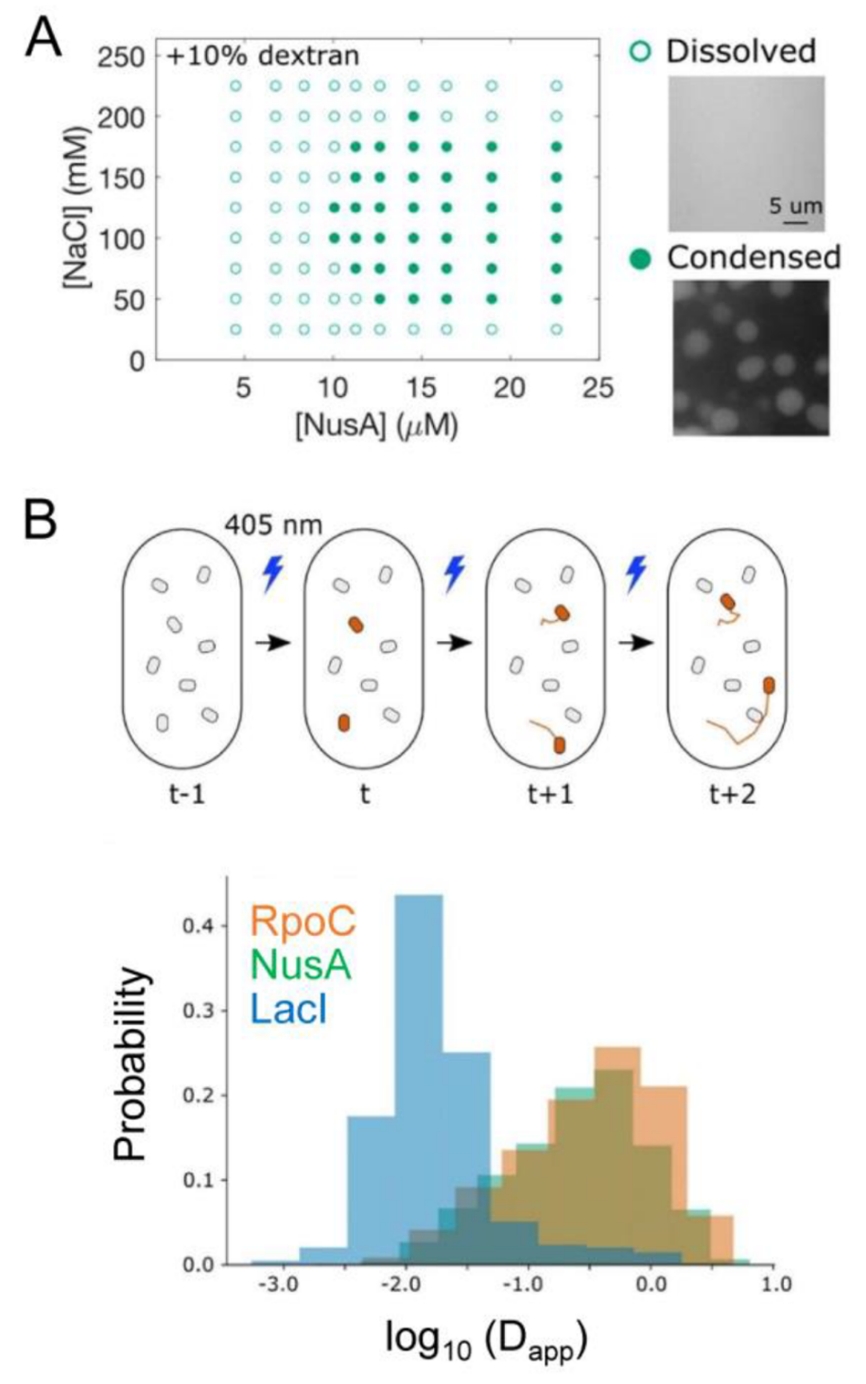
**Formation of biomolecular condensates by NusA and dynamics of components of RNAP clusters.** (A) Phase diagram for purified NusA in the presence of dextran. Open circles correspond to conditions in which the protein is dissolved, as in the image on the right (top), while closed circles indicate conditions in which the protein is condensed, as in the image on the right (bottom). (B, top) A cartoon depicting how single molecules of NusA are tracked over time in living *E. coli* cells. Cells expressing NusA fused to the photoconvertible fluorescent protein mMaple are continuously activated with 405 nm light, which photoconverts mMaple from a green-emitting form to a red-emitting form, allowing single NusA-mMaple molecules to be tracked over time. (B, bottom) Distribution of *D*_*app*_ (apparent diffusion coefficients) for fluorescent fusions of RpoC, NusA, or LacI that were tracked over time, showing faster movement of the former two compared with DNA-bound LacI. Figure adapted from ref (145). Copyright 2020 the Authors. Published by PNAS under Creative Commons Attribution-NonCommercial-NoDerivatives License 4.0 (CC BY-NC-ND) [CC BY-NC-ND 4.0 Deed | Attribution-NonCommercial-NoDerivs 4.0 International | Creative Commons].

RNAP forms clusters in *E. coli* that behave as biomolecular condensates arising from LLPS.^145^ This ability to form condensates is notable, as bacterial RNAP lacks the disordered C-terminal domain present in eukaryotic Pol II.^228^ RNAP condensates are prevalent in cells during the logarithmic phase and gradually disband once cells reach the stationary phase. The RNAP clusters seem to be independent of the folding of the chromosome into a compact structure, emerging instead from weak protein–protein interactions that involve the transcriptional antiterminator protein NusA. NusA exhibits phase separation *in vitro* and *in vivo* (Figure 9), enhanced by its modular architecture with multiple folded domains connected by flexible linkers and the presence of protein and RNA binding domains.^145^ Biomolecular condensation of NusA is driven by crowding, and phase diagrams in solutions containing 100 g/L dextran show that it is regulated by protein concentration and salt (ionic strength). LLPS of NusA *in vivo* is observed through cellular foci nucleated by protein–protein or protein–RNA interactions, whose size depends on protein concentration. Experiments *in vivo* suggest that another protein in the antitermination complex, NusB, may also be involved in the phase separation of RNAP.

Single-molecule tracking demonstrated that proteins within the RNAP condensates are highly dynamic,^145^ indicating the general usefulness of this technique to study biomolecular condensates in living bacterial cells. Broad distributions of diffusion coefficients were observed for the RNAP β′ subunit (RpoC) and NusA. In the case of RpoC, this distribution is likely the result of different activity states, including molecules engaged in transcription or nonspecifically bound to DNA, in agreement with other reports.^229,230^ These proteins had higher diffusion coefficients with a wider distribution compared with that of a DNA locus, indicative of slower diffusion compared to the proteins (Figure 9). Some overlap between the distributions of the proteins and the DNA was observed, likely corresponding to protein molecules of RpoC engaged in active mRNA transcription and NusA molecules engaged in transcriptional antitermination. The ability of RNAP to undergo LLPS in bacterial cells has important implications for transcriptional regulation and subsequent rRNA processing in bacteria, in response to internal and external cues.

## 4 Crowding and Phase Separation in Membrane Environments

**Figure 10:**
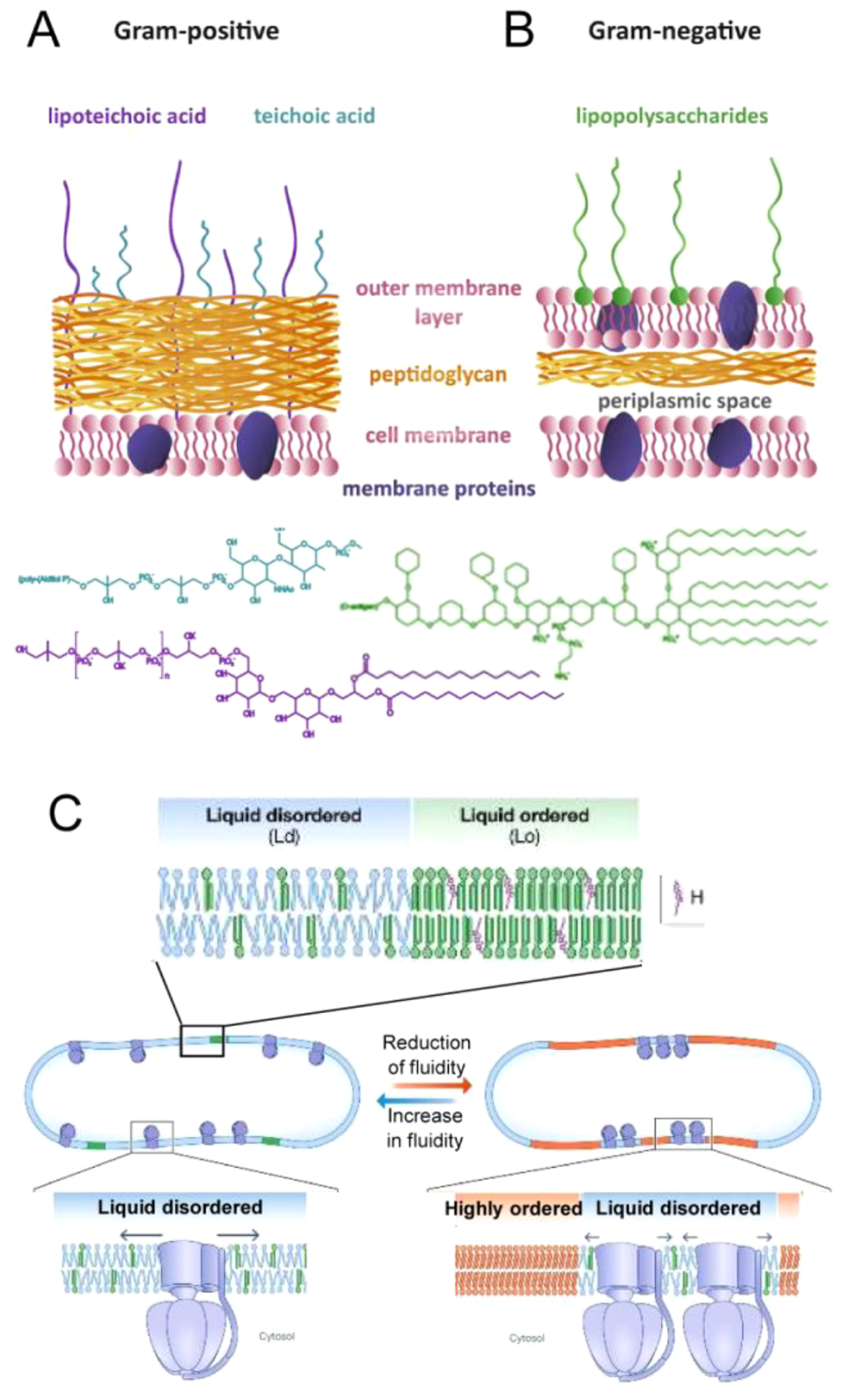
**Structure of the bacterial cell envelope and fluid state of the membrane.** (A and B) Models of Gram-positive (A) and Gram-negative (B) cell envelopes. Adapted in part from ref (237). Copyright 2019 the Authors. Published by Springer under the terms of the Creative Commons Attribution 4.0 International License [http://creativecommons.org/licenses/by/4.0/]. (C) Reversible phase separation induced by reduction of membrane fluidity. Bilayers are typically in the liquid-disordered phase (Ld, blue), but they can phase separate into liquid-disordered and liquid-ordered phases (Lo, green) when, e.g., hopanoids are present. Both are fluid phases. Extreme fluidity reduction triggers massive phase separation into highly ordered Lo phases within large parts of the membrane, forcing membrane proteins into the fluid phases. Under these conditions the membrane maintains its integrity and semipermeability. Adapted and modified with permission from ref (234). Copyright 2022 the Author.

Numerous cellular processes involve biological membranes, which facilitate the local concentration of highly ordered functional complexes at defined positions. In bacteria, the cytoplasmic membrane is an asymmetric bilayer of variable phospholipid and glycolipid compositions, depending on the species, and occupied by a high amount of integral and membrane-associated proteins^231^ (Figure 10). Gram-negative bacteria such as *E. coli* have an additional outer membrane that protects cells against harsh environments, contributes to their mechanical stability, and excludes many types of antibiotics, enhancing antibacterial resistance.^232^ The outer membrane consists of an asymmetric bilayer, with an inner leaflet of phospholipids and an outer one of lipopolysaccharides (LPSs) and membrane proteins.^233^ Bacterial membranes, whether from Gram-positive or Gram-negative bacteria, can contain hopanoids (sterols, equivalent of cholesterol in mammalian cells), pentacyclic triterpenoid lipids that are thought to enhance membrane integrity and impermeability by condensing the membrane.^231^

Membranes are characterized by the dynamic localization of lipids and proteins. The diversity of lipid acyl chains [saturated, (poly)unsaturated, branched, and cyclopropane rings] results in different membrane packing densities and fluidities.^234^ Membranes are normally in the form of liquid phases with highly dynamic lipids, but liquid–liquid demixing is observed in membranes of living cells composed of saturated and unsaturated acyl chains, as well as with certain other types of lipids, such as the aforementioned hopanoids, organized in liquid-ordered and liquid-disordered domains^234^ (Figure 10). Lipid rafts, nanoscale domains associated with the formation of liquid-ordered regions, are well characterized in eukaryotic cells^235^ but have also been observed in *B. subtilis* cells.^236^

### 4.1 Remodeling of the Membrane by Crowding and Phase Separation

The effects of macromolecular crowding on the structure and dynamics of biological membranes, including those of bacteria, have been comprehensively reviewed recently.^49^ It is clear that the multifaceted effects of crowding pervade over multiple length scales. Crowding in the membrane reduces the diffusion of membrane proteins and increases their clustering, which can alter their function. Crowding in solution increases membrane adsorption of proteins and modulates the protein:lipid affinity accordingly. The asymmetric crowding of one side at a membrane, for example by binding a protein at the periphery of a membrane, can cause membrane remodeling by inducing curvature leading to vesicle formation, as well as induce lipid-phase separation. The high protein density in and at the membrane thus has the potential to affect a plethora of protein-associated processes and may play a role in tuning higher levels of membrane organization.

Physicochemical properties of biological membranes largely depend on changes in the environment such as temperature, osmolarity, etc. Consequently, proper cell function relies on homeostatic regulation to preserve vital membrane features such as fluidity. Living organisms regulate their lipid composition in response to changes in temperature through reversible phase separation, which can result in formation of specific membrane domains into which some proteins partition and others are excluded. In bacteria such as *E. coli* or *B. subtilis,* an overall low membrane fluidity induced by alteration of fatty acid composition triggers large-scale lipid phase separation and promotes segregation of normally dispersed integral membrane proteins into the fluid areas.^238^ Extreme changes in lipid fatty acid composition, more drastic than those in the normal adaptation mechanism to temperature shifts, can lead to very low membrane fluidity that reaches the limit for cell viability.^238^ This lipid phase separation results in partitioning of membrane-associated proteins into the liquid membrane regions, affecting protein function (Figure 10C). For example, membrane fluidity affects the localization of MreB and FtsZ, key proteins of the membrane-associated elongasome and divisome complexes, respectively, perturbing cell morphology. Whereas membrane fluidity changes do not affect cell division in *B. subtilis*, similar changes in *E. coli* cells result in a defect in divisome assembly. Conversely, membrane fluidity changes perturb the cell wall synthesis machinery in *B. subtilis* but not in *E. coli*. Low membrane fluidity has no detectable effects on chromosome replication and segregation in *B. subtilis*, although some effects on nucleoid compaction have been observed in *E. coli*, possibly related to perturbation of RNase E (see section 7).

Localization of the phospholipid cardiolipin at the cell poles of rod-shaped bacteria has been proposed to occur by microphase separation produced by osmotic pinning of the membrane to the cell wall.^239^ Unlike individual lipids, large lipid domains of finite size generated by such phase separation gain the ability to sense cell curvature, favoring their spontaneous localization to the most curved areas of the cell (the poles). The biophysical model of Mukhopadhyay et al. shows the dependence of lipid domain localization on size distribution, which with increasing lipid–lipid short-range interactions becomes larger and narrower.^239^ The relationship between localization and strength of the pinning is determined by the balance between the osmotic pressure difference along the membrane (resulting from gradients of osmolyte concentrations, environmental variables, and some growth processes) and the inward force exerted by the cell wall. Heterogeneity in membrane pinning facilitates localization of lipid domains in cellular regions with reduced osmotic pressure differences. In support of this idea, cardiolipin relocalizes from the cell poles, where osmotic pressure differential is high, to the midcell division septum, where osmotic pressure is predicted to be lower, during *B. subtilis* sporulation.^240^ This model also predicts a critical concentration for formation of cardiolipin domains. For example, *E. coli* with reduced cardiolipin content loses polar localization of both cardiolipin itself and the osmoregulatory integral membrane protein ProP.^241^

**Figure 11:**
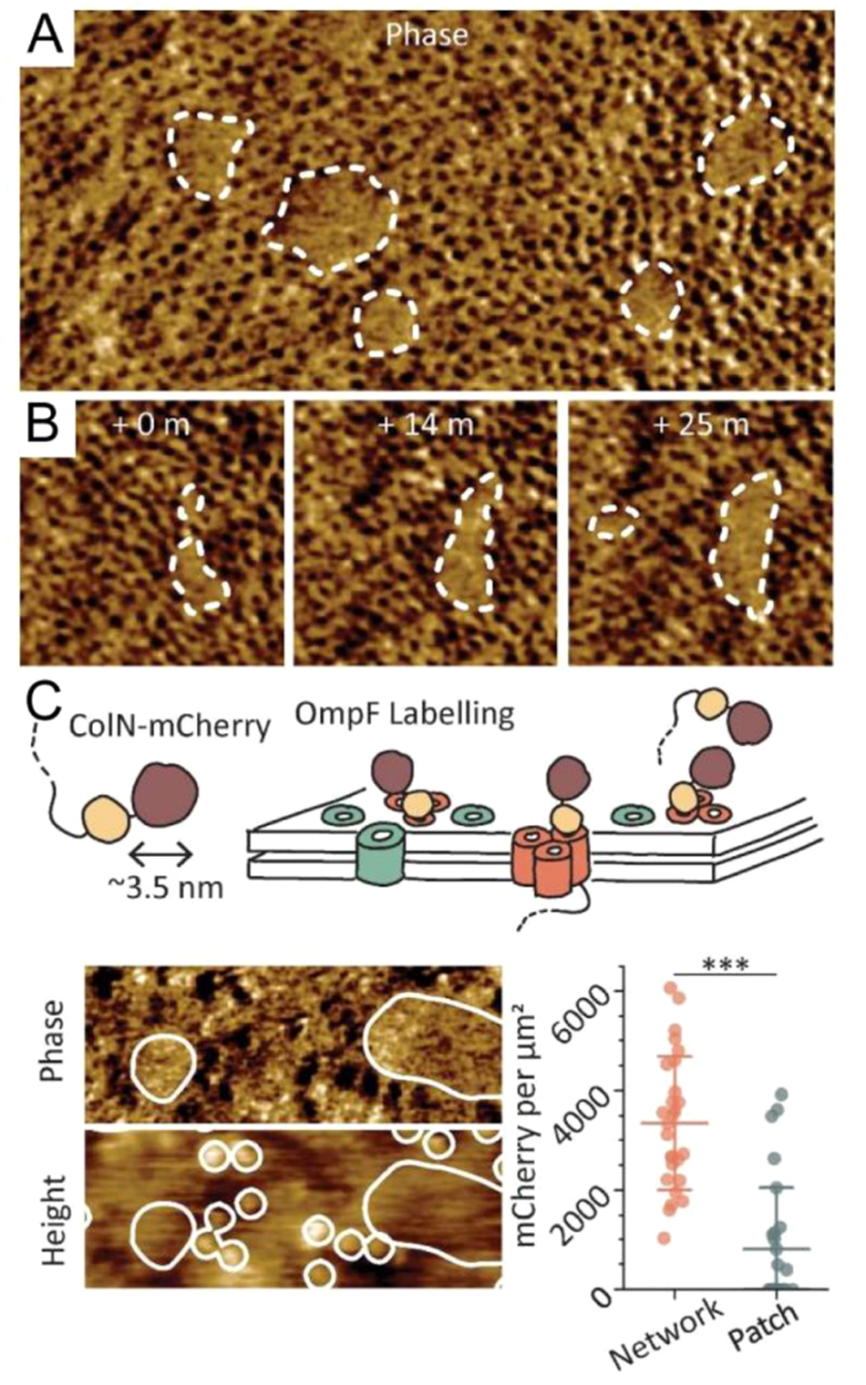
**Outer membrane of*****E. coli*****contains protein-free LPS patches.** (A) AFM phase image with phase-separated LPS patches highlighted by dashed lines. The pores identify the protein network surrounding the patches, formed by porins as OmpF. (B) At time scales consistent with cell division, under these experimental conditions, patches merge, grow, and split apart. (C) Illustration of OmpF labeling by colicin N^1–185^mCherry, used to localize the porin within the membrane surface in the height image. The phase image of the same area is used to localize the patches. Quantification of the labels per area shows that OmpF colocalizes with the pore network. Reprinted in part with permission from ref (242). Copyright 2021 PNAS.

Imaging by atomic force microscopy (AFM) of the entire external membrane surface of live and metabolically active *E. coli* has identified large-scale networks of proteins. Key components of the outer membrane such as the porin OmpF are distributed throughout, interrupted by small gaps of phase-separated LPS that merge, grow, and split with time (according to a liquid phase behavior) while maintaining their location^242^ (Figure 11). The surface fraction occupied by the lipopolysaccharide phase is dependent on concentration and LPS-LPS interaction strength. Modulation of the levels of the most abundant proteins has a clear impact on the amounts of pores formed by the porins. Disruption of lipid asymmetry by mislocalized phospholipids at the surface induces formation of new phases that deform the membrane,^242^ likely altering its barrier function and rendering cells more susceptible to some antibiotics.^243^ Along the same lines, molecular dynamics simulations propose that polymyxin B, a lipopeptide with antimicrobial activity, loosens the packing of the LPS external membrane upon binding, which triggers the flipping of phospholipids from the inner to the outer leaflet.^244^ This results in phase separation of the outer leaflet, with defects at the boundaries between LPS and phospholipid domains because of the hydrophobic mismatch that facilitates internalization of polymyxin B toward the inner membrane.

### 4.2 The Membrane as a Facilitator of Biomolecular Condensation

There is increasing evidence that many protein and protein–nucleic acid clusters assembled at the membrane display the hallmarks of biomolecular condensates. The role of membrane surfaces as key factors acting in the regulation of phase separation, along with post-translational modifications, has been analyzed in studies focused principally on eukaryotes (see Snead and Gladfelter^51^ and references therein). According to these studies, membranes generally lower the concentration threshold for biomolecular condensation,^245^ likely because they restrict diffusion to two-dimensions, although it is also possible that specific factors present in membrane boundaries may nucleate condensation and spatiotemporally regulate phase separation through changes in their distribution.^51^ In addition, it has been proposed that membranes can locally control the stoichiometry of elements within condensates and alter their dynamic properties and functions.^51^ Condensates in turn can drive membrane remodeling, suggesting that there may be an interdependence between lipid organization and the condensation of proteins and nucleic acids.^51^

**Figure 12:**
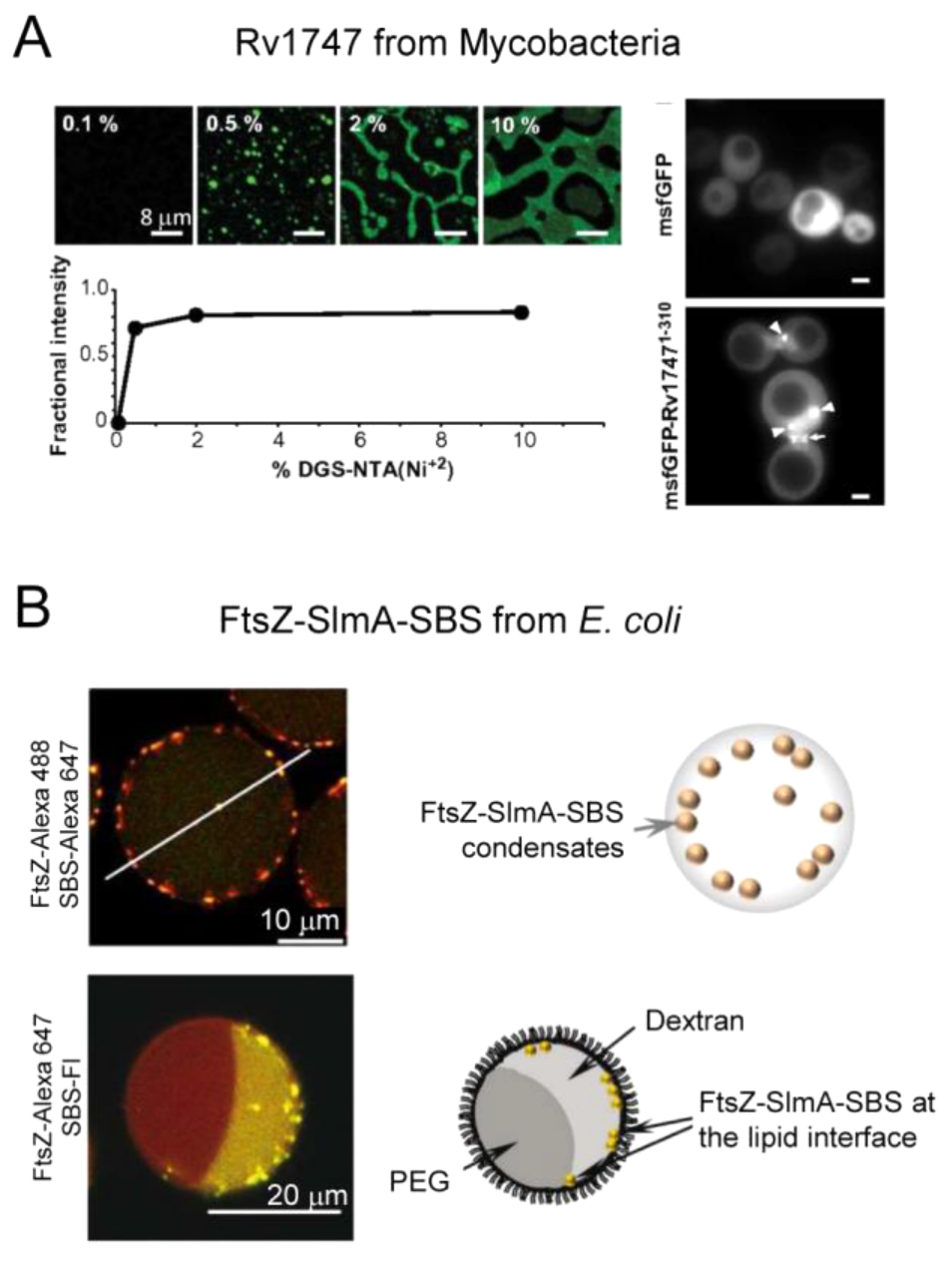
**Biomolecular condensates formed by integral or amphitropic proteins at the lipid membrane.** (A) (top, left) Fluorescence images showing spontaneous clustering of Rv1747^1–310^ on supported lipid bilayers. Nonphosphorylated His_6_-tagged OG-Rv1747^1–310^ is anchored to the DGS-NTA(Ni^2+^) within the lipid bilayers. (bottom, left) Quantification of the phase separation by the fractional fluorescence intensity vs weight percentage of the NTA(Ni^2+^) lipid. (right) Clustering also occurs in yeast, as shown by the arrowheads in the fluorescence images of cells expressing msfGFP-Rv1747^1–310^, in contrast to cells expressing msfGFP. Reprinted in part with permission from ref (246). Copyright 2019 PNAS. (B) Representative merged confocal images of the encapsulated FtsZ-SlmA-SBS nucleoprotein condensates into microfluidics-based microdroplets stabilized by the *E. coli* lipid mixture, showing preferential membrane location in a homogeneous crowding model generated with dextran (top) and in a compartmentalized cytoplasm model generated by a binary PEG/dextran LLPS system (bottom). The distribution of the condensates within the encapsulated systems is depicted on the right. Top, partly reproduced from ref (247). Copyright 2023 the Authors. Published by the Royal Society under the terms of the Creative Commons Attribution License [http://creativecommons.org/licenses/by/4.0/]. Bottom, partly reproduced with permission from ref (248). Copyright 2018 the Authors.

Examples of biomolecular condensates at the bacterial membrane can also be found *in vivo* and in cytomimetic systems. One such example is the integral membrane ATP-binding cassette (ABC) transporter Rv1747 protein from Mycobacteria, a virulence factor whose cytoplasmic regulatory module forms biomolecular condensates.^246^ This module can assemble into higher-order oligomers, depending on the phosphorylation state of its intrinsically disordered domain that bridges two 2 phosphothreonine-binding Forkhead-associated domains. Interestingly, phosphorylation enhances the reversible phase separation of this protein and modifies the dynamic properties of the resulting condensates, probably because of its impact on the self-association of the transporter. This is in line with the idea that post-translational modifications are a key cellular mechanism enabling the control of biomolecular condensation,^51^ suggesting that this principle may be extended to the kingdom of bacteria. The cytosolic domain of the Rv1747 transporter also forms biomolecular condensates when attached, through a histidine tag, to supported lipid bilayers containing the lipid 1,2-dioleoyl-*sn*-glycero-3-[(*N*-(5-amino-1-carboxypentyl)iminodiacetic acid)succinyl] (DGS-NTA) (Figure 12). Foci of this transporter are also observed in the cellular membrane upon heterologous expression in bacteria and yeast. Notably, the full-length protein assembles into clusters in Mycobacterial membranes that are more dynamic than those of the cytoplasmic regulatory module, suggesting that the transmembrane and nucleotide-binding domains may regulate the material properties of the condensates. Condensation in this system appears to have a functional role, as serine/threonine protein kinases and phosphatases colocalize differently with biomolecular condensates of the transporter: the kinases are homogeneously distributed within the condensates, while the phosphatases form foci at condensate interfaces.

Other integral membrane proteins that assemble into condensates are SpmX and PodJ, both involved in the regulation of asymmetric division in *C. crescentus* (see section 6.3). Mediated by their respective intrinsically disordered regions, SpmX forms biomolecular condensates on its own and with the pole-organizing protein PopZ, resulting in the regulation of DivJ kinase activity in response to nutrient availability.^150^ Furthermore, SpmX antagonizes phase separation of the polar organelle development protein PodJ, which forms condensates whose fluidity is possibly regulated by the membrane.^249^

In addition to integral membrane proteins, amphitropic proteins able to interact peripherally with the membrane can also form biomolecular condensates in bacteria. As part of their functional interactions, some of these proteins also bind nucleic acids, concomitantly with the membrane or in a competitive manner, participating in the overall regulation of phase separation. This is the case for the single-stranded DNA-binding protein (SSB)^250^ involved in DNA replication (see section 6.1) and the nucleoid occlusion factors from *B. subtilis* and *E. coli*, Noc^147,251^ and SlmA,^248,252^ respectively, important for proper positioning of the cell division ring (see section 6.3). In the case of SSB, reversible foci lacking DNA at the membrane of *E. coli* cells disband upon DNA damage,^250^ compatible with biomolecular condensates negatively regulated by DNA binding.^253^ This suggests a model in which phase-separation at the membrane would serve as a mechanism to store SSB in an inactive state when the levels of its ssDNA substrate are low.^253^

The role of lipid membranes in the biomolecular condensation of bacterial proteins has been addressed through reconstitution of nucleoid occlusion factors SlmA and Noc in minimal membrane systems. When heterotypic nucleoprotein condensates of SlmA are encapsulated inside cell-like microfluidics microdroplets that display crowding and compartmentalization in the lumen and are stabilized by *E. coli* lipids, they preferentially localize at the membrane boundary^248^ (Figure 12). Condensation of these cell division proteins is enhanced by lipid surfaces, as determined by reconstitution in supported lipid bilayers.^254^ In fact, the enhancing effect of the lipid membrane is also observed with FtsZ alone, as incipient formation of FtsZ condensates in microdroplets occurs in conditions under which no condensates are formed in bulk.^255^ Similarly, condensates of Noc interact with the outer membrane of giant unilamellar vesicles (GUVs), with the membrane monolayer of water-in-oil droplets in which the protein is encapsulated, and with supported lipid bilayers.^147^ Membrane binding seems to stabilize Noc condensates that display a notable preference for the more flexible liquid disordered domains, and negatively charged lipids significantly increase phase separation. Noc condensates also change the physical properties of the membranes. Therefore, as in eukaryotic cells, some membrane-associated condensate forming proteins in bacteria have the ability to modulate, and be modulated by, their membrane partners.

## 5 Comparison of Prokaryotic and Eukaryotic Cytoplasmic Structures

Comparison of prokaryotes with eukaryotes often provides insights into general principles of biochemical organization. Most studies on biomolecular condensates and biochemical organization of the cytoplasm have been conducted in eukaryotes due to their relatively large size, which permits formation of larger condensate structures and facilitates their imaging. The prokaryotic and eukaryotic domains of life share many biochemical similarities despite the hallmark macroscopic structural differences. Notable examples of the similarities are major metabolic pathways; the proteostasis machinery, with major chaperones having homologues in both domains; the machinery and mechanisms of macromolecular synthesis (DNA replication, transcription, and translation); as well as energy transducing machinery and signal transduction, which are highly conserved across different kingdoms of life.

Prokaryotes and eukaryotes are enormously diverse, and comparison based on model systems can become anecdotal. For example, the two most common model systems to represent prokaryotes and eukaryotes, *E. coli* and HeLa cells, differ significantly in size, but some plant cells have a cytosol that is only 100 nm in diameter, as the vacuole takes up most of the cytoplasm.^256^ This cytosol is almost 10 times smaller than the diameter of *E. coli*, and hence more similar to those of *Pelagibacter* species, which is one of the smallest (and most abundant) bacterial species on Earth. Eukaryotic organelles such as the endoplasmic reticulum (ER) and mitochondria have dimensions similar to bacteria, but there is a tremendous diversity among organelles depending on cell function. We will thus compare eukaryotes and prokaryotes with these limitations in mind.

### 5.1 Cell Volume

*E. coli* has a volume of 0.5–2.0 μm^3^, whereas a HeLa cell reaches 500–4000 μm^3^.^63^ This difference in cell size has a number of consequences for biochemical organization, most notably that a smaller volume limits the number of molecules needed to achieve high concentration: a HeLa cell needs ∼2,000 more molecules to reach the same concentration as *E. coli*. A small cell volume induces more confinement effects, and combined with the lower number of molecules, this reduces the size of biomolecular condensates and aggregates in bacteria. For example, polyQ-containing proteins grow into aggregates with dimensions corresponding to the cell diameter of *E. coli* (about 1 μm)^257^ and can be more than 5 μm in diameter in HEK293T cells, depending on the expression level.^258^ In general, the size of biomolecular condensates can be expected to scale with the cell volume.^259^ Although small bacterial cells generally have higher surface to volume ratios compared with most eukaryotic cells, the intracellular membrane systems of the latter compensate for this with a high membrane surface area that can promote more condensate adsorption or formation. For example, condensation of an RNA-binding protein is promoted at the ER membranes, and the properties of these condensates are modulated by the presence of RNA.^260^ Condensate-like clusters also occur at the plasma membrane during the formation of F-actin.^261^ In these cases, a condensate scaffold component is proposed to be tethered to the membrane. Such membrane tethering is analogous to the bacterial RNA degradosome that forms condensates on the bacterial cytoplasmic membrane,^144^ suggesting that condensate tethering is a more general strategy in all cells.

### 5.2 Diffusion and Active Transport

The small size of most bacteria allows them to rely solely on passive diffusion as the main mode of intracellular transport. As mammalian cells are larger, they evolved an additional active transport network where myosins carry cargo along actin filaments, and kinesins and dyneins along microtubules.^262^ The importance of the cytoskeleton is underscored by the notable abundance of actin and tubulin in such cells. The cytoskeletal network enables long(er) distances to be reached for large cargo (vesicles and organelles) rapidly, which is especially relevant in axons, flagella, and other cell extensions. It has recently been proposed that most vesicles, which are in the 25-nm-size range, similar to that of ribosomes and other supramolecular complexes, rely on passive diffusion in a normal mammalian cell.^263^ The mammalian cell is less crowded than bacterial cells such as *E. coli* and can therefore maintain >3 times higher diffusion coefficients (*vide infra*). The distance a particle travels by Brownian motion is determined by the diffusion coefficient:
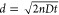
7where *d* is the distance traveled, *n* is the dimensionality of the confinement, and *D* is the translational diffusion coefficient.^68^ Although a mammalian cell is much larger than a bacterial cell, a molecule or complex rarely needs to travel from one end of the cell to the other but instead more locally between membranes or molecular complexes. Travel between compartments such as the ER, Golgi, mitochondria, and plasma membrane is shortened by large membrane surface areas, which increases the chance for membrane proximity and membrane contact sites. Hence, both mammalian and bacterial cells rely in large part on Brownian motion of their components.

In addition to being the highways of the cell, microtubules and other filamentous structures are major dynamic organizers of the cytoplasm of mammalian cells, as filaments are in bacteria. In both cell types, protein filaments are crucial in coordinating cell division (see section 6.3). In mammalian cells they provide mechanical strength and shape, which are largely provided by a rigid cell wall in most prokaryotes and eukaryotes such as fungi and plants. Cell walls are stronger and can withstand the higher pressures that these species have to endure. The cytoskeleton also provides additional organizational roles. For example, F-actin serves as a functional adhesion site for biomolecular condensates.^264^

### 5.3 Biomolecular Condensates

An emerging mode of dynamic organization is phase separation that results in biomolecular condensates. Here, proteins interact in a multivalent manner, driving phase separation. Proteins that undergo phase separation frequently have intrinsically disordered domains and heterotypic interactions with RNA. Intrinsically disordered regions (IDRs) modulate phase separation based on polymer-physics principles: comparatively unfavorable interaction with the solvent drives self-assembly of the polymers, reducing the energetic cost. Bioinformatics allows estimation of the percentage of proteins with extended disorder and found 28–42% in mammalian cells, 19–44% in yeast, and 4–29% in *E. coli*. While the numbers among studies vary widely, bacterial proteins have consistently less disorder than eukaryotes.^63,265^ IDRs in mammalian cells are often used in signaling, where their residues are phosphorylated or decorated with other post-translational modifications such as ubiquitination, glycosylation, lipidation, methylation, etc. Phosphorylation can determine whether a protein partitions into condensates:^266^ for example, NPM1 (nucleophosmin 1) phosphorylation drastically changes its interaction network, resulting in reduced partitioning in the nucleolus. In other cases, phosphorylation induces phase separation, such as condensation at a phosphorylated disordered domain of EGFR, a receptor tyrosine kinase.^267^ Kinases can also be recruited into condensates where they phosphorylate their target, which in turn modulates the condensate size.^268^ Also, in bacterial cells, many hydroxyl or nitrogen bearing amino side chains are phosphorylated and used for signal transduction. In *C. crescentus*, condensates are used as localized signaling hubs where phosphates are transferred between the participants for asymmetric patterning (described in more detail in section 6.3).^269^ The efficiency of this pathway depends on the material state of the condensate, with optimal performance and sufficient fluidity, which is governed by the IDRs and oligomerization domains of the scaffold protein PopZ.^146^

IDRs are, in principle, not needed for phase separation, as proteins with multiple interaction domains can form condensates similar to patchy colloids that undergo phase separation.^270^ Nonetheless, IDRs seem to be pervasive, for example, in the phase separation of the enzyme ribulose-1,5-biphosphate carboxylase (RuBisCO), which is mediated by the disordered protein Essential Pyrenoid Component 1 (EPYC1) of *Chlamydomonas reinhardtii* (green algae), which has multiple binding sites to connect multiple RuBisCOs.^271^ Similarly, disordered proteins assemble RuBisCOs in prokaryotic cells. For example, the intrinsically disordered protein CsoS2 assembles RuBisCOs through multivalent binding in the α-carboxysome from the γ-proteobacterium *Halothiobacillus neapolitanus*.^272^ In the case of the β-carboxysome from cyanobacterium *Synechococcus elongatus*, the RuBisCOs are linked by the protein CcmM.^273^ This protein has folded domains that bind RuBisCOs and has IDRs between the folded domains that function as linkers. Each RuBisCO specifically binds four CcmMs. The dynamic biomolecular condensate properties result from the disordered linker domain within CcmM, creating a network of RuBisCOs. McdB assists in positioning these carboxysomes in *S. elongatus*.^274^ This protein has been shown to phase separate also. This occurs through self-association with a coiled-coil dimerization and a trimerization domain while its IDR modulates its solubility. The functional relevance of self-assembly is not yet clear, but may involve tuning McdB binding to the carboxysome components, such as CcmM. NusA, an antitermination factor for RNA polymerase involved in rRNA synthesis, is one of the few proteins with high disorder in *E. coli*.^145^ It phase-separates *in vitro* and *in vivo* and may thereby nucleate RNAP foci (see also section 3).

RNA is a prevalent component in biomolecular condensates in eukaryotes, including stress granules and the nucleolus. mRNA half-lives are shorter in *E. coli* (4 min) than in mammalian cells (10 h) and comparable to *S. cerevisiae* (20 min).^63^ A short mRNA lifetime does not seem to prevent condensate formation, as RNA condensates containing stably incorporated mRNA are found in *S. cerevisiae*, such as P bodies,^275^ and in mammalian cells. RNA-containing droplets have also been found in various bacteria (see section 3), for example, in the form of the RNA degradosome.^276^ Moreover, RNAP condensates are formed through protein–protein interactions and are mostly involved in rRNA synthesis (in *E. coli*).^145^ This is particularly interesting because the nucleolus of mammalian cells is a separate compartment that produces rRNA.

In addition to these useful functions, biomolecular condensate formation can potentially be an intermediate step toward pathological protein aggregates.^46^ Notable examples of such behavior are the protein Huntingtin exon 1 associated with Huntington disease,^277^ tau associated with Alzheimer’s,^278^ and FUS associated with some forms of fALS.^279^ Preconcentrating such proteins enhances aggregation, although the probability of a transition to a fibrillar state will also depend on the chemical properties of the biomolecular condensate.^280^ Furthermore, it is unclear if these pathways are relevant beyond experiments with purified protein, high overexpression levels, or model cell lines. Nonetheless, biomolecular condensates have the potential to alter protein aggregation, and there is no reason to assume that this cannot occur in prokaryotes.

### 5.4 Molecular Density

Molecular density affects biochemical organization through macromolecular crowding effects, chemical interactions, and solvent quality. Molecular density is commonly measured by refractive index and, recently, by Raman imaging.^281,282^ The refractive index, which mostly reports on protein content, combined with volume measurements, indicates that *E. coli* maintains a macromolecular density of 300 mg/mL ± 15%.^107^ This compares to the 300–400 mg/mL biomacromolecule (protein + RNA) concentration obtained from cell dry weight.^7,283^ This is similar to fission yeast, which maintains a density of 280 mg/mL.^284^ In contrast, mammalian cells maintain a somewhat lower concentration of about 200 (90–260) mg/mL, as shown by a wide range of techniques and mammalian cell types.^281^ Normalized stimulated Raman imaging also reports on protein content.^282,285^ Using this method, the densities of the mammalian cytoplasm, nucleus, and nucleolus are 75, 85, and 115 mg/mL, respectively, and vary upon perturbations such as osmotic stress, ouabain treatment (inhibition of Na^+^/K^+^ ATPase), cytoskeleton disruption, cell senescence, and quiescence. Interestingly, the concentrations of protein in cell tissues vary: pancreatic islet maintains about 200 mg/mL, kidney glomerulus 100–200 mg/mL, skeletal muscle cells 200–300 mg/mL, and Zymogen granules in the pancreatic islet 300 mg/mL. Perhaps the matrix stiffness in different tissues reduces cell volume and thereby increases crowding.^286^ These findings suggest that measurements of immortal cell lines on glass slides or in suspension have less relevant densities. Determining protein concentration requires separate cell volume measurements, which can be challenging given the variety of cell shapes, and sample preparation (e.g., fixation) may generate artifacts. Nonetheless, if mammalian cells in tissues indeed have higher density, they may be more similar in density to cells of other domains of life.

The protein density is related to macromolecular crowding. Density is usually the weight per volume, whereas macromolecular crowding is the volume taken up by the bystander macromolecules providing steric hindrance. Macromolecular crowding is a function of the steric properties of the macromolecules, their number density, and how they are organized and can be measured by diffusion or dedicated probes.^17^ Diffusion of GFP suggests there is lower crowding in mammalian cells than bacterial cells (*vide supra*), which matches the density measurements. The lower crowding in mammalian cells has also been confirmed by a macromolecular crowding sensor (unpublished). The biochemical organization can strongly increase macromolecular crowding effects^287^ such as increased protein self-assembly.^287^

A common source of a change in density and crowding in most cells is osmotic stress. Fundamentally, the different domains of life have a similar response: release of water to the extracellular environment with a higher osmolality leads to a reduction of cell volume, which increases crowding, ionic strength, and internal osmolality.^288,289^ Cells recover volume through uptake of potassium ions and compatible solutes from the medium, as well as synthesis of other noncharged molecules such as sugars over the longer time frame. Full crowding recovery and adaptation takes half an hour to hours in *E. coli* and HEK293T, as shown with a FRET-based macromolecular crowding sensor.^17,66^ Cell growth already resumes before the crowding stabilizes at a new level. Hyperosmotic stress is one of the most frequently used perturbants in the laboratory to generate phase separation in mammalian cells. Phase separation may be induced by increased concentration of phase-separating proteins, macromolecular crowding, a change in ionic strength, or an active response of the cell. For example, the eukaryotic protein WNK1 kinase phase separates due to the macromolecular crowding in cells with hypertonicity, which activates a signaling pathway for cell volume recovery.^290^ As protein condensation has been less investigated in bacteria, osmotic stress-induced phase separation has not been described yet to the best of our knowledge. It is, however, known that hypertonic stress leads to nucleoid condensation.^291^

### 5.5 Stickiness

Weak and native associative interactions can alter protein stability or trigger formation of (transient) protein assemblies, as has been shown for purinosomes or G bodies in eukaryotic cells. Stickiness can also arise from nonspecific (hydrophobic, electrostatic) interactions between macromolecules,^292^ where, for example, chaperones bind to exposed hydrophobic surfaces or unfolded proteins expose their hydrophobic regions to stick to the cell’s biomacromolecules. Human cell lines possess a more extensive and complex chaperone and proteostasis system compared to prokaryotes and may have a different stickiness profile than bacteria, which are, on the other hand, more crowded.

When biomacromolecular surface chemistries are incompatible, it can cause misfolding, aggregation, and phase separation. Generic nonspecific interactions, or stickiness, lead to lowered diffusion, which can be tuned by the charge of biomacromolecules, as shown for the set of charged GFPs.^88^ The internal ionic strength depends on the bacterial species (section 2.1.3) and further tunes these interactions. The regulation of protein surface properties through mutation, i.e., the tuning of protein stickiness, is required in the presence of macromolecular crowding. Cellular macromolecules have coevolved over many generations, which may have led to “optimal stickiness”, but this is not the case when new proteins are introduced (e.g., by heterologous expression). In-cell NMR measurements have shown that amino acid substitutions in a Cu/Zn superoxide dismutase (SOD1) did not significantly impact its stability in eukaryotes but did in bacteria.^293^ Differences in stability of macromolecules in mammalian and bacterial cell lines can be related to the lower macromolecular crowding in eukaryotes.

Translational diffusion modulates diffusion-limited reactions and depends strongly on the physicochemical characteristics of the macromolecules such as stickiness and crowding, and these differ for numerous bacterial and mammalian cell types. Indeed, the less crowded mammalian cells allow faster translational motion of fluorescent proteins than bacteria do: fluorescent protein diffusion in various bacteria is in the range of 3–12 μm^2^/s, whereas it is 27 μm^2^/s in fibroblast cells and 24 μm^2^/s in *Dictyostelium discoideum*,^68^ compared with 87 μm^2^/s in aqueous media. The diffusion in bacteria is more in the range of that in the ER lumen, which is 5–10 μm^2^/s.^294^ In both eukaryotes and *E. coli*, diffusion depends on stickiness, where supercharged cationic GFPs have been shown to stick to ribosomes. In human cells, a positively charged peptide fused to GFP has a lower diffusion coefficient in the vicinity of F-actin.^88,142^ The diffusivity in *E. coli* shows stronger dependence on the charge of an introduced protein than in mammalian cells,^295^ probably due to higher crowding providing shorter distances for sticky, electrostatic interactions. Also, rotational diffusion (i.e., the rotation of a molecule along its own axes) of human SOD1 barrel is lower in *E. coli* than mammalian cells,^296^ but the same rotational diffusion coefficient is found for GFP.^294^ Homologously expressed bacterial TTHA rotates freely in *E. coli*, whereas heterologously expressed HAH1 does not. Here, the most important factor is the intracellular context, which has coevolved with the native protein, whereas a protein that is not in its native environment may experience enhanced stickiness and thus a slowed rotation.^295^ Amino acid substitutions have been shown to increase the rotational diffusion coefficients of HAH1 and SOD1 in bacteria, apparently by reducing their stickiness in the cell.^296^

Aside from stickiness and additional weak and transient molecular interactions, there are significant differences in other structural levels of cellular protein organization. Whereas prokaryotes have smaller proteins on average than mammalian cells,^63^ recent predictions based on Alphafold2 indicate a higher degree of protein homo-oligomerization in bacteria than in mammalian cells. According to Schweke et al.,^297^ 45% of the *E. coli* proteome forms homo-oligomers, compared to 20% in human cells. We suggest that bacteria use more noncovalent homo-oligomerization, such as ATP-binding cassette (ABC) transporters consisting of self-assembled dimers that originate from a single short gene, whereas equivalent genes in eukaryotes have been duplicated and fused. Indeed, most ABC transporters in *E. coli* are homodimers, whereas they are fused in mammalian cells.^298,299^

### 5.6 Ribosomes

Cells contain a high concentration of ribosomes, and it has been proposed that they reduce diffusion of particles in the range of 20–40 nm. Cryo-TEM measurements suggest that the cytosolic concentration of ribosomes in yeast cells is exceptionally high, at 23 μM or 20% of the cytosolic volume.^162^ The concentration drops to 13 μM when cells are treated with rapamycin. Rapamycin targets mTORC1, which prevents mTORC1 from sensing amino acids and controlling ribosome concentration. The ribosome concentration estimated for *E. coli* is 10 μM, which is close to that of yeast.^8^ The ribosome concentration for mammalian cells has been estimated at 1 μM,^300^ but this concentration may be less accurate, as the cell volume was not measured precisely. Rapamycin reduces both diffusion and protein phase separation in yeast and mammalian cells; in yeast, the diffusion coefficient increases 1.8-fold, and in human HEK293 cells, 1.25-fold. In addition, there is an 80% and 50% decrease in SUMO_10_-SIM_6_ droplet area in yeast and HEK293 cells, respectively. As the cytoplasmic ribosome concentration may be very low in mammalian cells, this would suggest an additional mechanism, such as the presence of mRNA, that determines the viscosity. Indeed, a recent study by Xie et al. shows that mRNA condensation upon stress, such as carbon depletion, increases the diffusivity of proteins in the cytosol.^301^ Barriers presented by mRNA organization may in fact dominate over the ribosome crowding effects proposed earlier.

By analogy with rapamycin treatment in eukaryotic cells, ATP depletion in *E. coli* cells reduces the diffusion of particles larger than 30 nm,^155^ although the mobility of GFP is not. The size dependence is similar to that of a colloidal glass transition. The same reduction in diffusion can be seen in yeast, where energy depletion reduces the diffusion of the same viral matrix particles as in *E. coli*.^192^ Munder et. al suggested this is caused by an acidification of the cytoplasm, and Joyner et al. suggested an increase in macromolecular crowding.^158,160^ Later TEM images showed major changes in the yeast cytoplasm upon energy depletion, including more lipid droplets, membrane invaginations, membranous structures, and fibrillar aggregates,^302^ each of which could present roadblocks for larger diffusing particles. This is likely similar to an aging yeast cell, where similar large ultrastructural changes were seen.^303^ Of note, the direction of the diffusion change of these particles is strongly dependent on the particle identity,^301^ which may be due to a change in proteomic stickiness upon ATP depletion.^304^ This suggests that other phenomena may play a role.

### 5.7 Ionic Strength

In the cell, ionic strength plays a crucial role in organizing biomacromolecules. As biomacromolecules are charged, ion pairing between their residues increases affinity and specificity in protein–protein and protein–polynucleotide interactions. However, counterions screen the charge of these residues and need to be replaced during binding. Hence, counterions play a role in protein–protein and protein–polynucleotide interactions, as well as complex coacervate formation. *E. coli* has a cytoplasmic ionic strength of ∼300 mM, which compares to ∼140 mM for the cytosol of mammalian cells.^18,63^ Counterions need to be removed for charged proteins to interact, which costs energy and is less favorable at higher counterion concentration. Furthermore, a higher ionic strength leads to increased Debye screening of the protein charge, resulting in shorter-range attraction between opposite charges. This assumes the ions to be inert point charges, but the identity of small molecule anions also matters, as it can determine preferential interactions as given by the Hofmeister series. Glutamate is the predominant anion in *E. coli*, whereas chloride is the most abundant anion in mammalian cells. Glutamate is more kosmotropic (Terminology) than chloride and should interact less with proteins.^305^ Indeed, preferential exclusion of glutamate from a single-stranded DNA binding protein enhances condensation of this protein, whereas chloride interacts with the protein and therefore reduces condensation.^306^ Moreover, kosmotropic salts such as sodium fluoride can enhance the phase separation of the RNA-binding protein FUS, whereas chaotropic salts such as sodium bromide and sodium iodide inhibit it.^307^ Therefore, the specific ion interactions and ionic strength together may alter the biochemical organization of the cytoplasm in eukaryotes in a different manner than in prokaryotes.

Next to ionic effects, interactions with species-specific and electrostatically neutral osmolytes also have the potential to affect solvent quality and potentially trigger phase separation. Here, the effect of the solutes is highly solute specific, which is determined by how well they are hydrated and mostly how much they directly interact with a protein and with which moieties (amide or side chain).^308^ Common kosmotropes such as glycine betaine and trehalose are thus excluded from the protein surface, stabilizing the proteins. These are thus called compatible solutes and used in the different domains of life.^309^ They are vital when the intracellular solute concentration needs to be increased upon hypertonic stress. Chaotropic solutes such as urea are less common in cells. Urea is a waste-product in mammals but can be a nitrogen source for bacteria.^310^ Indeed, buffer experiments show that TMAO, which is a common cosolvent in deep sea fish and highly kosmotropic, enhances phase separation of γ-d-crystallin, whereas urea inhibits it.^311^ Because the cell’s small molecule composition is highly species- and (stress) condition-dependent, the interactions of small molecules with the biomacromolecules that drive biochemical organization will vary in different species and conditions.

## 6 The Bacterial Cell Cycle Machinery

### 6.1 Effects of Crowding and LLPS on Chromosome Replication

Replication of the bacterial chromosome is an essential cell cycle process that ensures faithful duplication of the genetic material to pass onto daughter cells, which is followed by segregation and completion of cell division. Chromosome replication is driven by a protein machine, the replisome, that acts bidirectionally to duplicate the DNA, from the chromosomal origin of replication (*oriC*) to the terminus of replication (*ter*), in three stages: initiation, elongation, and termination.^312^ The replication process in bacteria is exquisitely coordinated by crosstalk mechanisms with chromosome segregation and cell division, partially overlapping with them, as a means to rapidly proliferate and survive.^313^ The impact of macromolecular crowding on some of the multiple systems involved directly or indirectly in replication has been described. So far, a protein involved in the process, (ss)DNA-binding protein (SSB), has been shown to form biomolecular condensates. It would not be surprising if other proteins participating in chromosome replication will also be found to undergo phase separation, given their multiple domains of homo- and heteroassociation and their ability to form complexes with long DNA chains (single or double stranded) or to bind membranes, features commonly observed in proteins prone to phase separation.

#### 6.1.1 Crowding and Chromosome Replication

Various studies in bacteria and other microorganisms have indicated that DNA replication has a strong dependence on macromolecular crowding.^314−316^ One of these studies demonstrated that crowding increases the activity of *E. coli* DNA polymerase I, a processive enzyme that participates in the joining of Okazaki fragments during lagging-strand replication and in repair of damaged DNA.^317^ Addition of PEG 8000, dextran T-70, Ficoll 70, or bovine plasma albumin as crowding agents enhances the reaction rates of nick-translation and gap-filing by the enzyme, counteracting the ionic strength-dependent reduction of activity observed at KCl > 0.1 M in dilute solution, or with other salts. Smaller molecules such as glucose, sucrose, or low molecular weight PEG have lower or no effect on DNA polymerase I activity. Crowding remarkably decreases the apparent K_M_ values of DNA polymerase I for DNA, counterbalancing the increase in K_M_ observed at high ionic strength in dilute solution and presumably enhancing the binding of the polymerase to DNA. These early results suggest that crowding could act as a metabolic buffer on macromolecular interactions, extending the range of intracellular conditions to which bacteria can adapt.

Crowding also seems to play a crucial role in regulating the precise timing of chromosomal replication initiation by the protein DnaA.^318^ This protein is an ATPase that binds to specific DNA sequences within *oriC*, leading to the assembly of the replication complex in all known eubacterial species. DnaA is active when bound to ATP and inactive in its ADP-bound form. The exchange of ADP for ATP is stimulated upon interaction of the protein with the lipid membrane or with specific sequences on the chromosome.^319−321^ By using fluorescent analogs of ATP, it was found that high concentrations of Ficoll 70 accelerate the exchange of ATP on the membrane-bound DnaA.^318^ Thus, a crowding effect at the interface between the membrane and the aqueous phase, where the protein is located, accounts for the highly cooperative shift from a relatively slow to a rapid nucleotide exchange. Crowding would probably enhance DnaA oligomerization, consistent with the known tendency of this protein to self-associate,^322^ although stabilization of a compact conformation cannot be ruled out. In addition, it is possible that interactions with other proteins are facilitated by crowding *in vivo*, but this remains to be confirmed.^318^

#### 6.1.2 Phase Separation and Chromosome Replication

**Figure 13:**
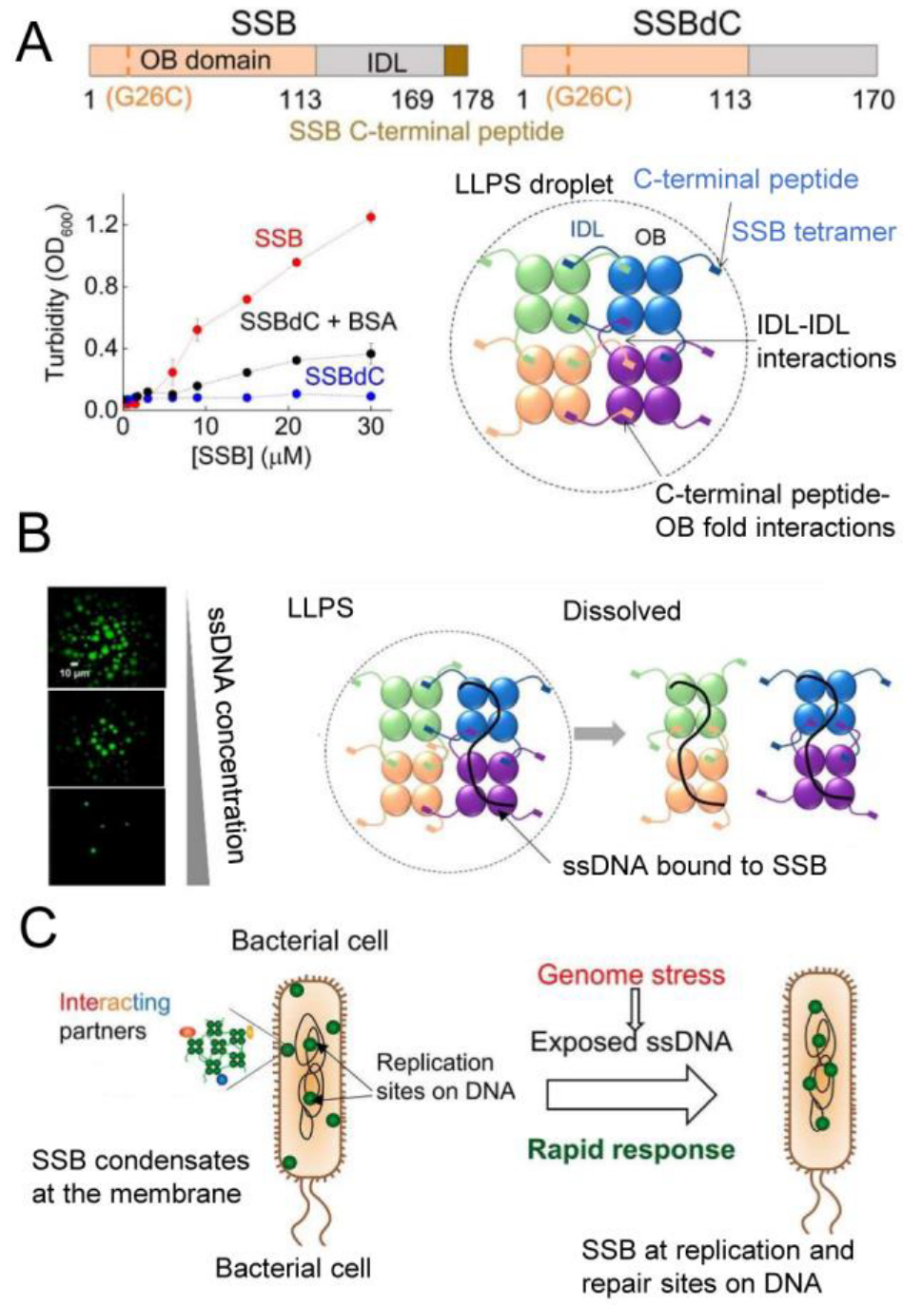
**Formation of biomolecular condensates by SSB and regulation by ssDNA.** (A) Multifaceted interactions of SSB structural regions are required for efficient LLPS. Schematic domain structures of SSB constructs are shown at the top, with numbers indicating amino acid positions at boundaries of structural regions. The SSBdC construct lacks the C-terminal peptide region. Below, turbidity is shown as a function of protein concentration, in the absence and presence of BSA (150 g/L), along with a model of LLPS-driving interactions. (B) SsDNA regulates SSB phase separation, as shown at the left by fluorescence microscopy of samples containing SSB, fluorescein-labeled SSB, and increasing concentrations of unlabeled dT_79_, and, on the right, a schematic model for the LLPS-inhibiting effect of ssDNA (black line). (C) Proposed model for the *in vivo* role of SSB LLPS, based on data from refs (253) and (250). Figure adapted from ref (253). Copyright 2020 the Authors. Published by PNAS under Creative Commons Attribution-NonCommercial-NoDerivatives License 4.0 (CC BY-NC-ND) [CC BY-NC-ND 4.0 Deed | Attribution-NonCommercial-NoDerivs 4.0 International | Creative Commons].

Bacterial SSB, essential for chromosomal DNA replication and repair, has been shown to form biomolecular condensates.^253^ The intrinsically disordered linker of SSB is required for condensation, and the interactions of its conserved ssDNA binding domain and C-terminal peptide upon self-association of the protein enhance the process, as do glutamate ions.^306^ SSB condensation occurs in the absence of DNA (Figure 13). In contrast to many other examples of phase separation either aided or disfavored by nucleic acid binding, in this case the role of DNA depends on the ssDNA:SSB stoichiometry. *In vitro,* SSB phase separates at low ssDNA:SSB ratios and, under these conditions, DNA partitions into the condensates. Increasing the ssDNA:SSB ratio inhibits phase separation, caused by competition between ssDNA and the SSB C-terminal domain for binding to the ssDNA binding domain. SSB partner proteins such as the DNA repair protein RecQ strongly partition into the SSB condensates, including those with low binding affinity (*K*_d_ in the tenths of micromolar range), although specific interaction is required. Small molecules such as nucleotides also accumulate to a slight degree inside the condensates, and the diffusion of clients within them scales with their size. Besides its canonical interaction with ssDNA, SSB enrichment inside the condensates also enables binding to RNA despite its lower affinity for SSB compared to ssDNA. This suggests that SSB may have a role in RNA metabolism.

Formation of SSB condensates has been analyzed *in vitro,* in dilute solutions containing glutamate and with BSA or PEG as crowding agents, and in cell extracts.^253^ As is typical for condensates assembled in bacteria, *in vivo* confirmation of these condensates remains challenging due to their small size. Theoretical estimations by the authors^253^ show that the maximum diameter of an intracellular SSB condensate would be ∼120 nm, if the entire pool of cellular SSB molecules (∼2,000 SSB tetramers) formed a single droplet. Although super-resolution microscopy approaches allow visualization of particles of this size, assessing their dynamic properties for compelling demonstration of LLPS behavior is not straightforward. Nonetheless, *in vivo* reports show the formation of SSB foci at replication forks and also near the cytoplasmic membrane,^250^ presumably reflecting the known interaction of SSB with membrane lipids. This suggests that condensation may provide a means to regulate SSB function, favoring its storage near the membrane at low local ssDNA concentration when DNA repair needs are minimal (Figure 13). When the free SSB pool exceeds the DNA-bound fraction, condensation would be expected to occur even at genomic DNA sites. An increase in cytoplasmic ssDNA, reflecting a demand for SSB in DNA repair, would dissolve the condensates and “release the guards” of the genome, enabling SSB to repair damaged DNA.

### 6.2 Effects of Crowding and LLPS on Bacterial Plasmid and Chromosome Segregation

**Figure 14:**
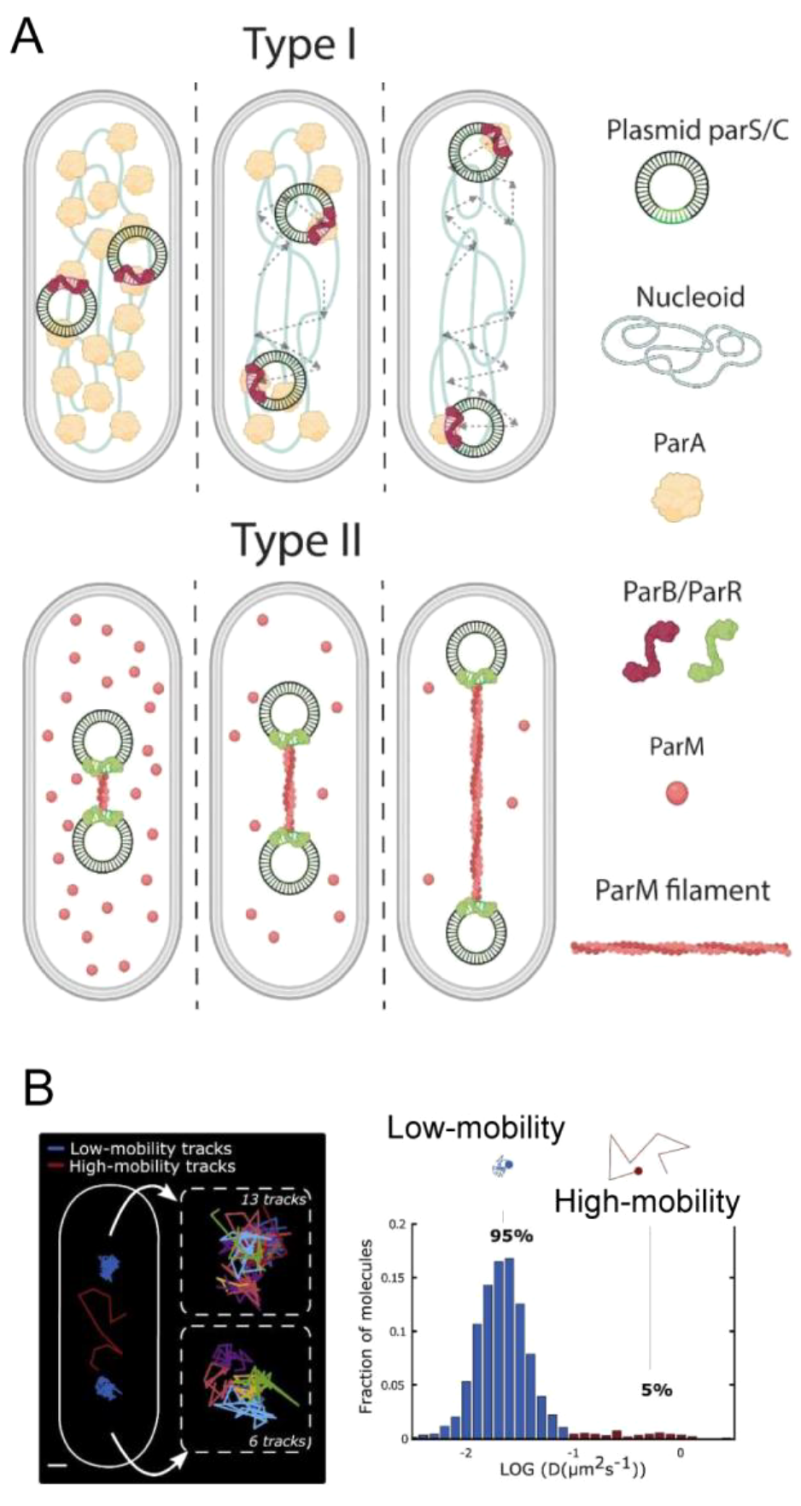
**DNA segregation and effects of phase separation.** (A) (top) Plasmid segregation by *parABS* following a pulling mechanism (Type I). ParB binds *parS* sequences on the plasmids, and the ParB-*parS* nucleoprotein complex moves poleward, with its attached plasmid, through interactions with ParA that is localized between ParB-*parS* and the poles. Dashed arrows depict the path of the plasmids. (bottom) Plasmid segregation by *ParMRC* through a pushing mechanism (Type II). ParR binds *parC* sequences on the plasmids. A ParM filament polymerizes from soluble monomers between the ParR-*parC* nucleoprotein complexes on a pair of plasmids and pushes them apart toward the poles. Reprinted in part and adapted from ref (324). Copyright 2021 Gogou, Japaridze, and Dekker under the Creative Commons Attribution License (CC BY) [CC BY 4.0 Deed | Attribution 4.0 International | Creative Commons]. (B) Dynamic properties of ParB condensates. (left) Representative image of a live cell (cell contour represented by a white line) with low-mobility (blue) and high-mobility (red) trajectories of single ParB molecules. Magnified views of each ParB condensate with different low-mobility trajectories are shown with different colors. (right) Histogram of apparent diffusion coefficients for low-mobility (blue) and high-mobility (red) trajectories. Reprinted in part with permission from ref (148). Copyright 2020 Elsevier Inc.

Prior to division, each future daughter bacterial cell inherits a fully replicated chromosome as a consequence of chromosome segregation. In many bacteria, this crucial process begins during replication with migration of the duplicated replication origins to opposite cell poles, followed by bulk segregation of the chromosome toward each cell pole and the resolution and transport of the replication termini at the division septum.^214^ Segregation is promoted by chromosomal macrodomains that further organize the DNA.^323^ Segregation mechanisms vary within bacterial species.^324^*C. crescentus*, *B. subtilis*, and *Vibrio cholerae* use an active pulling mechanism (Figure 14) that directs the chromosomes toward the bacterial poles through forces and directionality, using the dynamic *parABS* system. During growth of *C. crescentus* and *V. cholera*e, the ParB CTPase binds the *oriC*-proximal *parS* sequences in the chromosome, and the nucleoprotein complex thus formed moves poleward through interaction with the ParA ATPase, which seems to form a concentration gradient within the cell. In bacteria devoid of these active segregation systems, such as *E. coli*, spontaneous demixing of chromosomes occurs by entropic forces exerted on the replicating DNA.^325^

Segregation of plasmids, which are much smaller than bacterial chromosomes (1–1,000 kbp vs 1–10 Mbp^326^) is simpler and more widely studied. Segregation of most high-copy-number plasmids occurs through random Brownian motion.^327^ Low-copy-number plasmids, on the other hand, often encode dedicated segregation systems to maintain inheritance.^328,329^ One such system, plasmid P1 of *E. coli*, uses *parABS*(324) (*vide infra*). The other, plasmid R1 of *E. coli*, uses a mitotic-like mechanism powered by two proteins encoded by the plasmid, ParR and ParM; the latter is a homologue of actin. In this system, *parC* DNA sequences near the replication origin of the plasmids bind to the ParR protein, which in turn interacts with the ParM actin. As a result, R1 plasmid segregation is driven by ParM polymers, which connect a pair of plasmids through the ParR-*parS* interaction, pushing them toward opposite cell poles.^328^

#### 6.2.1 Crowding Promotes DNA Segregation

Entropy-driven segregation of the two replicated daughter chromosomes in rod-shaped bacteria has been modeled using two flexible ring polymers in the presence of cylindrical confinement and crowding agents.^330^ Crowders were simulated as spherical particles of MW 67 kDa, and the volume fraction of crowders (Φ) ranged between 0 and 0.3 to mimic the cell’s response to external osmolarity changes that cause dehydration of the cytoplasm, resulting in increased macromolecular crowding.^67^ In unsegregated polymers, contacts between them increase with higher Φ due to slower polymer dynamics. However, stronger crowding induces crowding particles to localize between polymer rings, enhancing ring–ring separation and increasing the mean residence time of separated rings at the cylinder ends. This is in agreement with theoretical predictions of entropic repulsion between overlapping segments of long polymer chains.^331^ According to Langevin dynamics simulations using a similar model, the segregation time was determined to increase with increasing Φ due to slower chain diffusion, whereas, for a fixed volume fraction, the segregation time decreases with increasing size of the crowders.^332^ Experiments with *E. coli* showed that protein oscillations exerted by the Min system can guide demixing of the chromosomes through interactions between MinD and DNA. Such interactions can enhance the entropic effects that, according to simulations, do not seem to be sufficient to drive full segregation on their own.^333^

Although the influence of crowding for each specific segregation step remains largely unknown, there are several reports that characterize the effects of crowding on overall segregation. For example, bacterial actin-like proteins, known as Alps, form polymers to promote segregation of various plasmids, and molecular crowding enhances their organization into complex structures.^334^ Supramolecular structures are also formed by AlfA protein from *B. subtilis* to segregate pBET131 plasmids during bacterial growth and sporulation,^335^ ParM polymers to segregate plasmid pSK41 in *Staphylococcus aureus*, as well as ParM polymers to segregate plasmid R1 in *E. coli*. All of these structures exhibit a multiplicity of states depending on nucleotide association, ionic strength, and pH. Besides electrostatic interactions through counterions with like-charged filaments,^336^ excluded volume effects from macromolecular crowding shift the equilibrium between single filaments and bundles.^337,338^*In vitro* studies have determined that R1-ParM bundling results mainly from molecular crowding, with a random distribution of filament polarity within the bundles stabilized by long-range electrostatic attractive forces between patches of residues.^339^ These properties presumably result in equally efficient DNA capture at both ends of the bundle. The increased stiffness of the filaments, their ability to handle large DNA cargos, and their structural plasticity^340^ are all factors that allow segregation to occur. Interestingly, ATP-triggered filament bundles formed by AlfA over a wide range of ionic strengths and pH values in dilute buffers were similar to supramolecular structures formed in the presence of crowding agents.^341^ As with pSK41-ParM, which also spontaneously forms bundles in the absence of crowders,^342^ the formation of the different kinds of polymorphic structures is thought to be mostly mediated by counterions.^343,344^ It is notable that bacterial actins work with a small number of associated regulatory proteins compared with the multiplicity of eukaryotic actin- or microtubule-associated protein modulators. Thus, it is attractive to postulate that in bacterial polymerizing systems, the greater functional degrees of freedom conferred by molecular crowding and counterions result in a greater diversity of filament–filament interactions, which obviates the need for numerous accessory proteins.^334^

#### 6.2.2 Direct and Condensate-Driven Effects of Phase Separation on Segregation

Phase separation-related demixing of the multiple DNA molecules found in a typical prokaryotic cell^345^ affects its internal organization and function. An artificial nanofluidic model has allowed quantification of the interactions of two dsDNA molecules in cavities with controlled anisotropy. The conclusion was that the two molecules spontaneously demix in elliptical cavities and orient along the poles with increasing cavity anisotropy.^346^ Mixing a large dsDNA molecule with a plasmid results in the exclusion of the plasmid toward the poles. Such an uneven distribution is enhanced by molecular crowding and is reminiscent of similar nonuniformity observed for high-copy-number plasmids in bacterial cells.^347^ Interestingly, a variety of large structures in bacterial cells, described as biomolecular condensates, foci, aggregates, etc., seem to often localize in zones excluded by the nucleoid. These structures appear at the cell poles, form in response to internal and environmental stresses^348,349^ (see section 7), and freely diffuse in the regions of the cytoplasm devoid of nucleoid,^139,350^ suggesting their localization might be influenced by segregation-induced entropic forces. One example of a protein involved in chromosome segregation^351^ that forms such structures under starvation conditions is the *E. coli* GTPase ObgE,^348^ which localizes in the cytoplasm and partly associates with the membrane.^351^

Phase separation has been described *in vivo* for the aforementioned *E. coli parABS* system that segregates plasmid P1.^148^ Similar to the chromosome segregation systems in other species, it consists of the DNA site *parS*, the DNA binding protein ParB, and the ATPase ParA. In *E. coli* cells plasmid *parS*-associated ParB forms nanometer-sized condensates whose fusion is prevented by the ATPase activity of the ParA motor.^148^ Two different dynamic behaviors have been found by using single-molecule tracking photoactivated localization microscopy (sptPALM) within these condensates: a low-mobility fraction of immobile ParB dimers bound to *parS*, and a high-mobility fraction of ParB dimers nonspecifically interacting with the DNA (Figure 14). Distribution of the replicated DNA along the cell length occurs upon ParA binding to the condensates that, accordingly, appear segregated. In a separate study, the effect of high pressure on the ParB condensates has been addressed in live *E. coli* cells by fluorescence intensity fluctuation-based methods, namely two-photon scanning number and brightness (sN&B) and raster scanning imaging correlation spectroscopy (RICS).^352^ Application of 100 MPa of pressure disrupts ParB condensates, some of which reassemble upon pressure release, indicating that they are reversible. Brightness analysis shows that the protein forms dimers in the condensates, disrupted by the application of pressure.

The ParB-*parS* partition complex of the *parABS* system was demonstrated to undergo LLPS *in vitro.*(353) In the presence of crowders such as PEG, dynamic round condensates of ParB from *C. glutamicum* are stabilized by the interaction with *parS*. Electrostatic interactions regulating ParB self-association seem to be involved in the formation of these condensates, because, as for many others, an increase in the ionic strength of the solution increases the saturation concentration needed for LLPS. As mentioned above, ParB binds and hydrolyzes the nucleotide CTP, and this CTPase activity is enhanced by interaction with *parS*. Interestingly, CTP stabilizes ParB condensates, significantly decreasing the saturation concentration for phase separation. This effect is specific for CTP, since nucleotides such as ATP or GTP, not recognized by ParB, disfavor condensation. This constitutes another example of phase separation promoted by the nucleotide CTP, as is the case for the nucleoid occlusion factor Noc of *B. subtilis* (see section 6.3). ParB homologues from other bacteria also form biomolecular condensates with analogous CTP regulation, suggesting an evolutionarily conserved mechanism for this protein in segregation. In *C. crescentus*, specific association of ParB to *parS* sites is controlled by the ATPase ParA, with the latter pulling the duplicated origin region toward the opposite cell pole. ParA concentrations at the new pole become thus slightly higher, triggering polymerization into a liquid phase-condensate of PopZ, the polar organizing protein that anchors ParB*S* to the pole.^354−356^

Chromosome segregation may be also affected by crowding effects and phase separation in other organizational systems that contribute to this essential process,^357^ its regulation, or its coordination with other cell cycle steps. For example, SMC (Structural Maintenance of the Chromosome) proteins, which are present in all bacteria as well as eukaryotes, organize and compact the DNA and probably mediate segregation by organizing replicated DNA into individual chromosomes prior to segregation. In *B. subtilis* and *C. crescentus*, SMC condensins interact with ParB^358,359^ bound to *parS* sequences. Distribution of SMC proteins in *B. subtilis* is modulated by XerC and XerD recombinases, which bind to the *dif* site at the chromosome replication terminus (*ter*) and catalyze the resolution of chromosome dimers that arise from replication.^360^ In *E. coli*, the SMC homologue MukB along with its partner proteins MukE and MukF organize in axial cores, including in cells with lower molecular crowding.^361^ The MukBEF complex binds to chromosomal sites everywhere except in the *ter* macrodomain, as a result of the antagonistic action of MatP protein,^362^ a key organizer of the *ter* macrodomain. In *B. subtilis* and *E. coli*, SMC proteins also interact with bacterial topoisomerases that contribute to chromosomal organization and segregation.^363,364^

Other factors driving segregation include systems that coordinate chromosome segregation with cell division. Among them are the divisome spatial positioning systems in *E. coli* such as the Ter-linkage mediated in part by MatP;^365^ a similar system has been characterized in *C. crescentus.*(366) The nucleoid occlusion effector in *E. coli*, SlmA protein, also coordinates cell division and chromosome segregation when bound to its specific sequences in MatP-free DNA regions outside of the Ter macrodomain.^367^ SlmA has been shown to form heterotypic condensates with the central division protein FtsZ *in vitro* (see section 6.3) that might ultimately affect its role in the coordination of segregation with division.

### 6.3 Effects of Crowding and LLPS on Bacterial Cell Division

**Figure 15:**
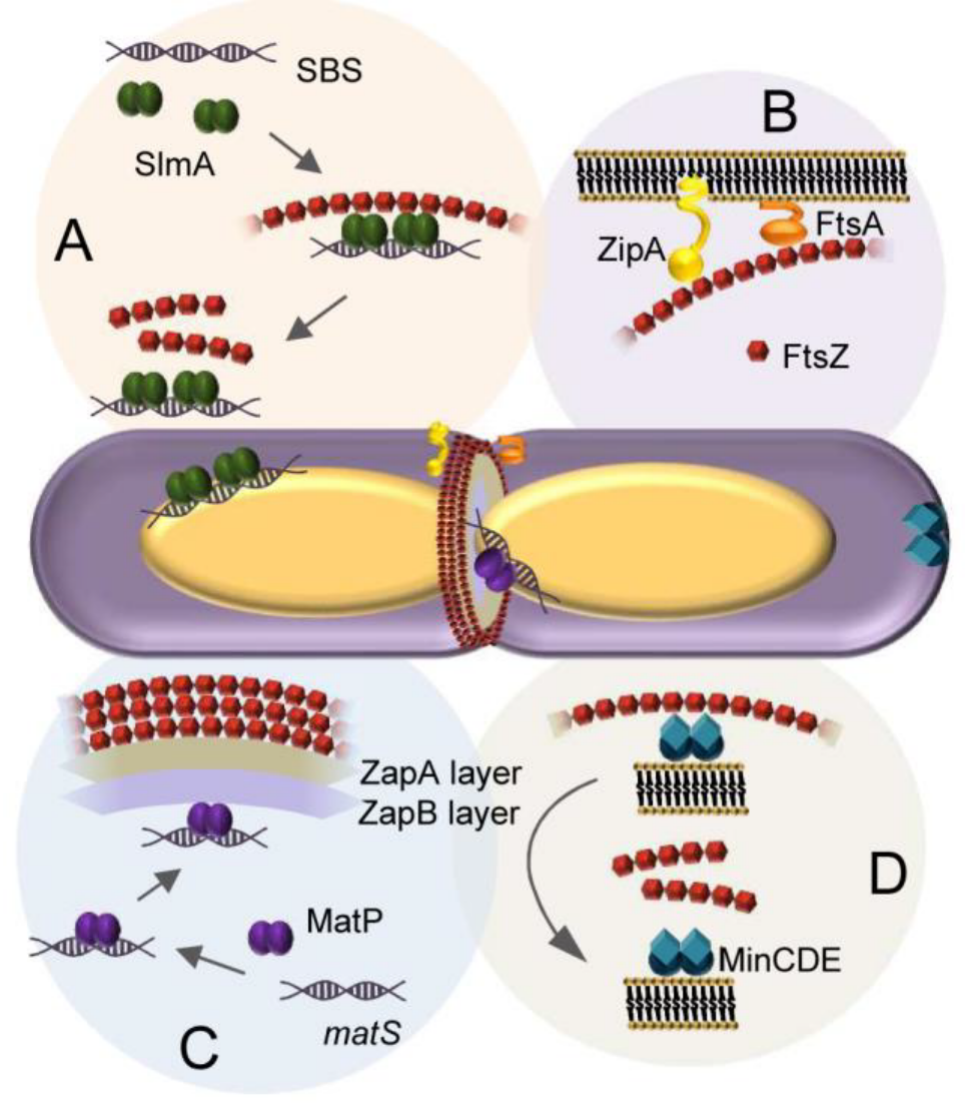
**Schematic representation of a dividing *E. coli* cell showing the FtsZ ring at midcell**. (A) Nucleoid occlusion, mediated by the protein SlmA bound to specific DNA sequences (SBSs) on the chromosome, antagonizes Z-ring formation near the chromosome. (B) Two proteins, ZipA and FtsA, anchor the Z-ring to the membrane. (C) The Ter linkage involving the proteins ZapA, ZapB, and MatP, which binds *matS* sequences at the Ter macrodomain of the chromosome, promotes Z-ring assembly at midcell. (D) The oscillatory MinCDE system, formed by the proteins MinC, MinD, and MinE, prevents Z-ring assembly at the cell poles. Adapted from ref (371). Copyright 2021 by the Authors. Published by MDPI, Basel, Switzerland under the terms and conditions of the Creative Commons Attribution (CC BY) license [https:// creativecommons.org/licenses/by/4.0/].

Most bacteria divide by binary fission, relying on a multiprotein machinery, the divisome, whose assembly is subjected to a precise regulation in time and space through the coordinated action of various protein factors (Figure 15).^368^ The cytokinetic ring is built by polymers of the protein FtsZ, a GTPase engaged in a complex scheme of reversible self-association reactions controlled by nucleotides, cations, and salt.^369^ Regulation of assembly of this “Z-ring” takes place through interactions of FtsZ with partners and ligands, some of which bind to a conserved C-terminal domain (CCTD) of FtsZ, which in *E. coli* interacts with at least 6 different proteins.^370^

#### 6.3.1 Macromolecular Crowding and Cell Division

The vast majority of the studies exploring the impact of macromolecular crowding on bacterial cell division have focused on FtsZ, because of its ability to form polymorphic structures of large size, alone or assisted by the many proteins with which it interacts, whose interconversion equilibria are susceptible to modulation by excluded volume effects.^372^

##### 6.3.1.1 Crowding and FtsZ Oligomers

The impact of crowding on the oligomerization of the GDP-bound form of *E. coli* FtsZ has been studied using nonideal tracer sedimentation equilibrium, a method in which the dilute species is labeled to distinguish it from the crowders.^373^ This oligomerization takes place according to an indefinite linear self-association model in which a Mg^2+^ ion is bound by each protein monomer added to the oligomer, and the affinity for monomer incorporation gradually decreases with oligomer size.^374^ This mechanism is radically different from that used for the cooperative formation of FtsZ polymers elicited by GTP.^375^ By using iodinated FtsZ as tracer, equilibrium gradients have been measured and analyzed to retrieve the apparent weight-average molar mass of this protein as a function of its concentration and of those of the crowders, BSA or cyanmethemoglobin.^373^ The two crowders tested interact with FtsZ exclusively via steric repulsion, having large effects on its association constants in the presence of Mg^2+^. The effects are particularly pronounced at high crowder concentration, leading to high oligomer sizes. Consequently, decamers and larger oligomeric species of FtsZ, only minimally represented in dilute solution, become more abundant in crowded conditions.^373^

Brownian dynamic simulations have been applied to study macromolecular crowding effects on the rates of FtsZ dimerization. In this approach, the rate constants in crowding conditions are obtained from the rate constant in the dilute solution, applying a factor that accounts for the crowding effect.^376^ Simulations show that crowding reduces the diffusion of FtsZ, due to the concomitant increase in viscosity. At crowder excluded volume fractions below 0.3, this reduction is somehow counteracted by crowding-related enhancing effects, resulting in negligible overall changes in the FtsZ dimerization rate constant. At excluded volume fractions of 0.3, however, the enhancing effects prevail and the dimerization rate constant is ∼4 times higher compared to that in dilute solution.

##### 6.3.1.2 Crowding and FtsZ Polymers

**Figure 16:**
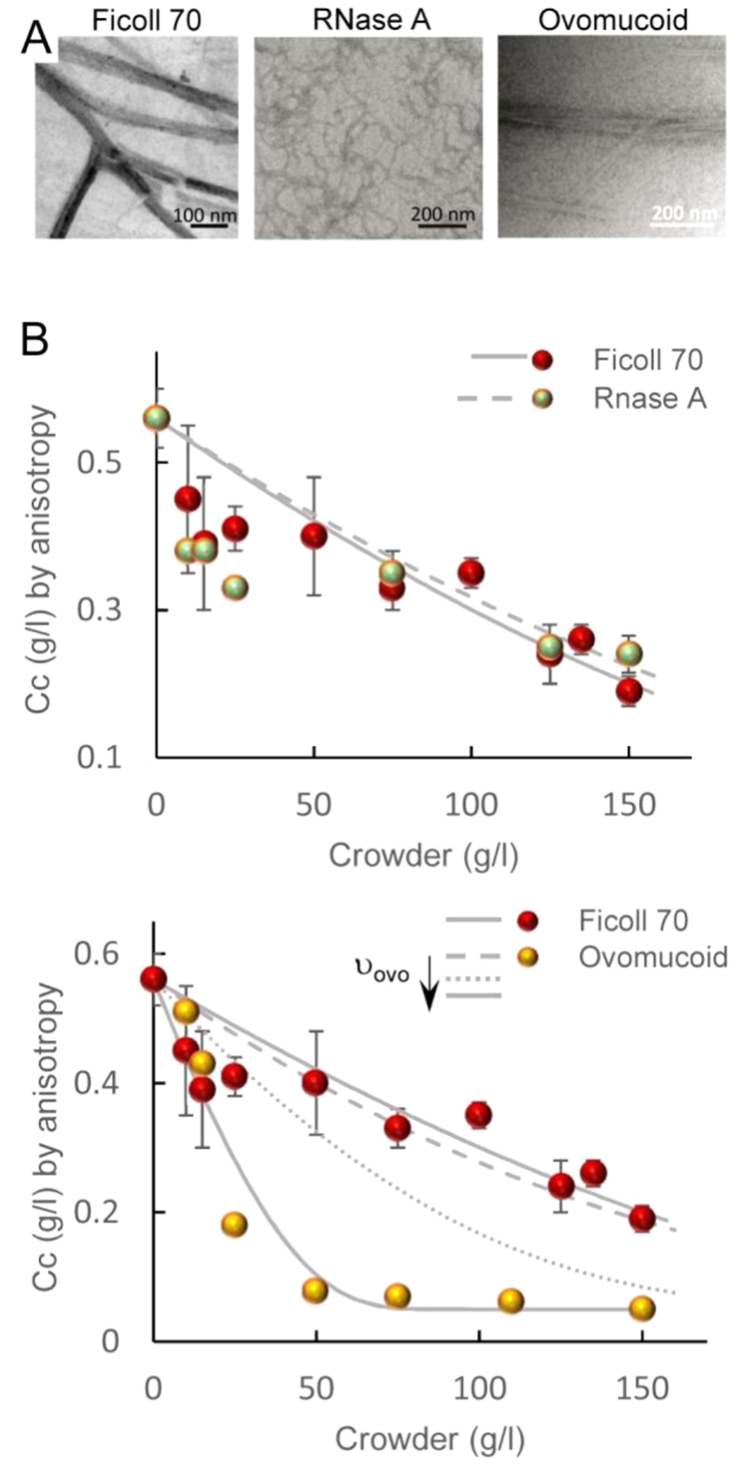
**Effect of crowders on the polymerization of FtsZ.** (A) Electron microscopy images of GTP-triggered FtsZ polymers in the presence of the specified crowders. (B) Variation of the critical concentration of polymerization (*Cc*) of FtsZ with the concentration of Ficoll 70, ovomucoid, and RNase A. Lines correspond to simulations according to a volume exclusion model, showing a pure volume exclusion behavior for Ficoll 70 (υ_Ficoll_ = 0.96 mL/g) and for RNase A (υ_RNase_ = 0.703 mL/g). Experimental data in the presence of ovomucoid cannot be explained in terms of a pure volume exclusion behavior (dashed line, υ_Ovo_ = 0.69 mL/g) or assuming repulsion with like molecules (dotted line, υ_Ovo_ = 1.61 mL/g), instead being compatible with a model assuming additional effects (solid line, υ_Ovo_ = 6.6 mL/g). Arrow in the legend depicts increasing volume exclusion. Adapted or reprinted in part from ref (378), copyright 2016 Monterroso et al. Published by PLOS under the terms of the Creative Commons Attribution License [CC BY 4.0 Deed | Attribution 4.0 International | Creative Commons], and ref (23), copyright 2003 Elsevier Inc. under the terms of the Creative Commons CC-BY license [CC BY 4.0 Deed | Attribution 4.0 International | Creative Commons].

Dramatic effects of crowding on GTP-induced *E. coli* FtsZ polymers, usually one subunit-thick under dilute solution conditions,^370^ have been reported by different laboratories. Electron microscopy and AFM images of FtsZ in the presence of high concentrations of model crowding agents like Ficoll 70 or dextran T70 evidence the formation of FtsZ bundles through lateral association of single-stranded protofilaments^23^ (Figure 16). These larger structures are energetically more favorable under crowding conditions than the protofilaments, as they exclude less volume. The bundles are still dynamic, but their disassembly rate and GTPase activity are lower compared to those of the protofilaments. In addition to linear bundles, rings and toroids of *E. coli* FtsZ have been described in an electron microscopy study, in which methyl cellulose or poly(vinyl alcohol) is used as a crowder.^377^

Polymerization of *Mycobacterium tuberculosis* FtsZ has also been scrutinized using these crowding agents.^379^ Variable arrangements of the same type as those observed with *E. coli* FtsZ are observed, including rings and toroids, in the presence of KCl. However, when more closely inspected, some structural features of these *M. tuberculosis* FtsZ assemblies are different from those of *E. coli* FtsZ, suggesting distinctive assembly mechanisms.^379^ Moreover, in the presence of Na^+^ ions, FtsZ from *E. coli* forms helical spirals, whereas for *M. tuberculosis* FtsZ the equilibrium is shifted toward long bundles. By using time-lapse TIRF microscopy, the rate of elongation of the FtsZ bundles from *M. tuberculosis* in crowding conditions has been determined. After an elongation phase, a steady state is reached after which the lengths of the bundles mostly decrease or remain unaltered.

Taken together, these studies indicate that the polymorphic nature of the GTP-induced FtsZ filaments is also maintained in crowding conditions. Their final arrangement strongly depends on conditions such as type and concentration of salts or pH and on the particular FtsZ protein being studied, similar to that usually observed for this protein in dilute solution.^380^ Importantly, all these studies conclude that crowding favors lateral interactions of FtsZ polymers, known to be a prerequisite for engaging in a functional Z-ring.^381,382^ Indeed, bacteria have proteins specifically devoted to the cross-linking of FtsZ protofilaments, acting as positive regulators of Z-ring assembly, the most important ones being the Zap proteins (ZapA, B, C, and D). As Z-ring formation needs to be restricted to the cell center at the time of cell division, mechanisms antagonizing the crowding-induced bundling are likely necessary *in vivo.*(23) Consistent with this, several negative regulatory proteins and systems in bacteria inhibit Z-ring formation at the wrong places in the cell. In *E. coli*, the two main spatial regulators are the Min system and nucleoid occlusion, which act on lateral interactions between FtsZ filaments as well as longitudinal interactions between FtsZ subunits within protofilaments.

The Min system of *E. coli* comprises the proteins MinC, MinD, and MinE that together block FtsZ ring assembly at the cell poles (Figure 15). Powered by ATP-driven bulk migration of MinD and MinE from one cell pole to the opposite cell pole, the concentration of the MinD-binding protein MinC over time ends up being highest at the cell poles and lowest at midcell, where the future Z-ring forms.^383,384^ The key regulatory mechanism is that direct interaction of MinC with FtsZ selectively inhibits Z-ring formation at the cell poles, thus helping to corral FtsZ polymers to midcell. The C-terminal region of MinC recognizes FtsZ,^385^ interfering with the lateral association of its filaments.^386^ The N-terminus of MinC, on the other hand, inhibits protofilament assembly,^387^ resulting in a two-pronged disruption of FtsZ protofilament bundles.

Co-reconstitutions of FtsZ and the Min system on lipid bilayers, together with the membrane tethering protein ZipA that interacts with FtsZ (Figure 15), have shown strong coupling between both systems, which is reflected in the formation of antiphase waves that are enhanced in crowding conditions.^388^ This behavior is consistent with the antagonistic regulation of FtsZ polymerization and bundling by the Min system, and its corralling of FtsZ to midcell.

FtsZ bundles formed in noncrowding conditions are also disrupted by SlmA,^389^ the protein that mediates nucleoid occlusion in *E. coli.*(390) Nucleoid occlusion, mediated by SlmA binding to several SlmA binding sequences (SBSs) on the bacterial chromosomal DNA, prevents FtsZ rings from forming over unpartitioned chromosomes and causing potentially catastrophic chromosome breakage (Figure 15). Notably, the nucleoprotein complexes of SlmA with its specific binding sequences accelerate FtsZ depolymerization in crowding conditions comparable to those found in the cytoplasm^247^ analogously to that described in dilute solution.^391^ This suggests that lateral interactions do not confer particular resistance to the antagonistic action of SlmA. Interestingly, the positive regulator ZapA (Figure 15) partially reverses the acceleration of FtsZ disassembly by SlmA/SBS, and it does so more efficiently in the presence of crowding agents.^247^ These results suggest that excluded volume effects might contribute to the regulation of Z-ring formation, reinforcing the agonistic action of specific factors, while not interfering with the antagonists, as they are designed to counteract crowding-related effects such as lateral interactions and bundling. We speculate that crowding could be one of the missing factors determining Z-ring localization, since in the absence of both negative (the Min system and nucleoid occlusion) and positive regulators, multiple discrete Z-rings still form and are biased toward the cell center.^392^

The polymerization of FtsZ by GTP occurs through a cooperative mechanism characterized by a critical concentration (*Cc*), which represents a threshold above which polymers are formed.^380^ Light scattering and fluorescence anisotropy based determinations of *Cc*(393) have shown that high concentrations of unrelated proteins, Ficoll, dextran, or sucrose decrease its value^378^ (Figure 16), consistent with the notion that crowding generally favors self-association. Evolution of the experimentally determined *Cc* with the concentration of the different crowders is compared in this study with simulations based on volume exclusion theory, assuming that FtsZ polymerization behaves as a first-order phase transition and with activity coefficients defined in terms of the exclusion volume (V_ex_), concentration, and masses of all species in the solution. The extent of crowding effects on the *Cc* agrees with the exclusion volume behavior (i.e., simulations using partial specific volumes of the species as V_ex_ are compatible with the experimental data) in the case of neutral inert polymers such as Ficoll or dextran and for proteins like RNase A when its own oligomerization is considered. In contrast, reductions in the *Cc* larger than expected for a pure exclusion effect are observed for ovomucoid as crowder and for DNA at relatively low concentration. Effects beyond excluded volume predictions may be partially attributed to additional electrostatic repulsion between negatively charged ovomucoid or DNA and FtsZ, which is also negatively charged at neutral pH.

**Figure 17:**
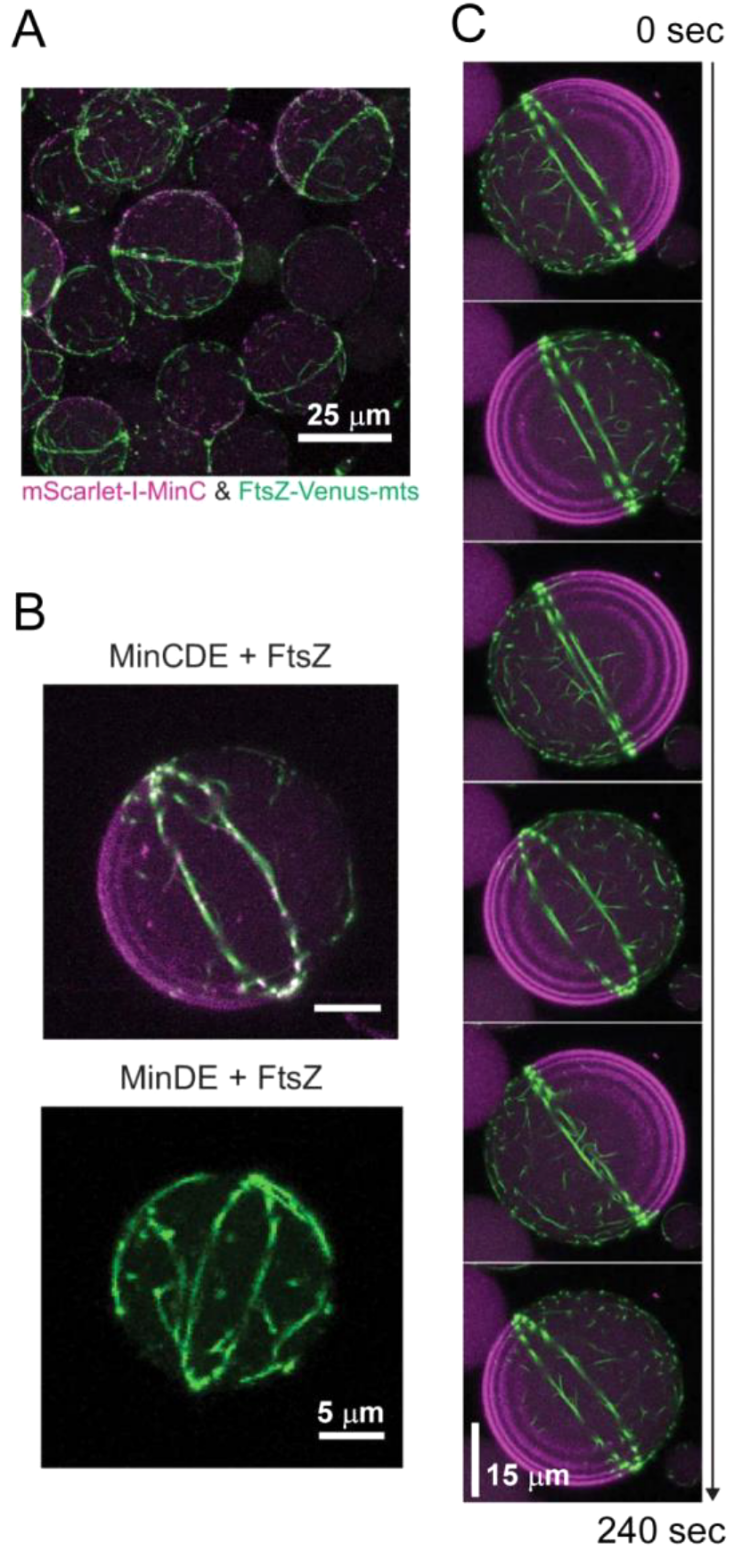
**Positioning of Z-ring by the Min system in vesicles.** (A) 3D maximum projection of a merged confocal image of vesicles containing the MinCDE proteins (mScarlet-I-MinC, magenta) and FtsZ-Venus-MTS (green) in dextran 70, showing that Z-rings are spatially restricted to the vesicle midpoint by the inhibitory action of the Min-oscillatory wave. The MTS is a heterologous amphipathic helix (membrane targeting sequence) fused to FtsZ-Venus that artificially tethers it to the membrane. (B) 3D projections of a Z-ring positioned by the MinCDE system (top) as in panel A, and a Z-ring that is still positioned at the vesicle midpoint, albeit less efficiently, by the Min system lacking MinC (bottom: Min waves are not visible because of the absence of mScarlet-I-MinC). (C) Time-lapse confocal images of the Z-ring (FtsZ-Venus-MTS, green) stabilized by the oscillatory pole-to-pole Min waves (magenta) as reflected by mScarlet-I-MinC. Adapted in part from ref (396). Copyright 2022 the Authors. Published by Springer Nature under a Creative Commons Attribution 4.0 International License [https://creativecommons.org/licenses/by/4.0/].

FtsZ polymers have also been studied inside microdroplets generated by microfluidics, through which hundreds of droplets of controlled size and composition are obtained, containing crowding agents coencapsulated with the protein and stabilized by *E. coli* lipids.^394^ The distribution of the fibrous protein networks was found to be dependent on FtsZ and crowder concentration. In both cases, increasing concentrations rendered a spread of the FtsZ polymer network, reducing the so-called depletion zone most probably generated by geometric and entropic restraints. Restrictions imposed by the spatial boundaries are also characterized by modifying the container shape. FtsZ has been later encapsulated inside lipid-stabilized microdroplets containing protein crowding agents or *E. coli* lysates.^395^ The appearance of bundles is observed in all cases, either from the beginning or after shrinkage of the microdroplets, leading to concentration of the crowding agents but also of FtsZ and, presumably, of the buffer components. Macromolecular crowding is one of the decisive experimental factors in the bottom up reconstitution of a minimal machinery for autonomous division, as shown in a study in which FtsZ and the Min system were encapsulated in lipid vesicles with crowding agents (Figure 17).^396^ This study provides a showcase of the emergence of cell division in a minimal system.

##### 6.3.1.3 Mixed Macromolecular Crowding and FtsZ

**Figure 18:**
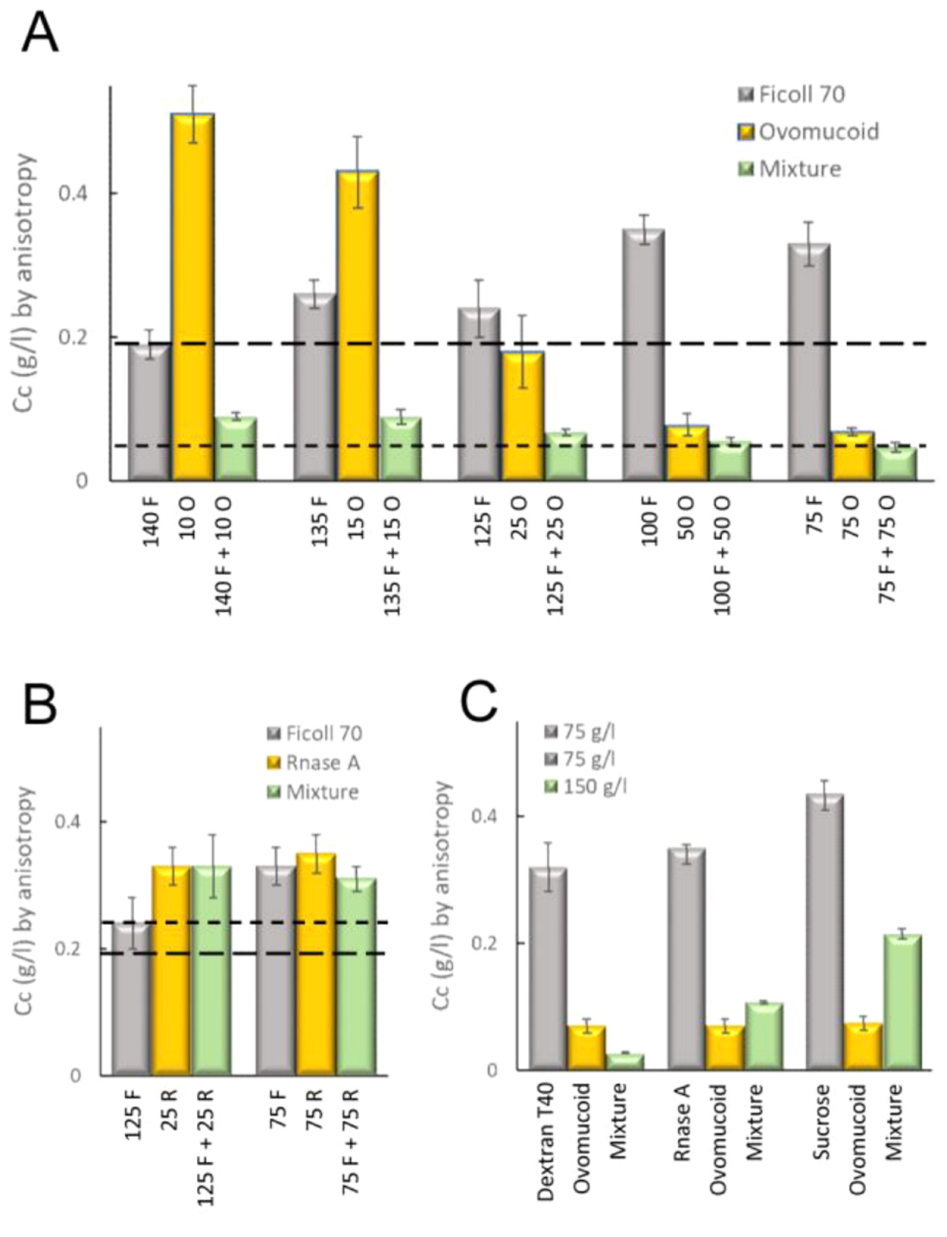
**Effect of mixed crowders, involving inert polymers and proteins, on the polymerization of FtsZ.** (A and B) *Cc* values determined in the presence of the specified crowders. F, O, and R are Ficoll 70, ovomucoid, and RNase A, respectively. The numbers in the *x-*axis are their concentrations in g/L, alone, or in the mixtures. Total crowder concentration in the mixtures is 150 g/L. Long and short dashed lines depict the *Cc* values in the presence of 150 g/L Ficoll (A and B) and ovomucoid (A) or RNase A (B), respectively. (C) *Cc* of FtsZ assembly in the presence of the specified individual crowders and their mixtures (50%). Adapted from ref (378). Copyright 2016 Monterroso et al. Published by PLOS under the terms of the Creative Commons Attribution License [CC BY 4.0 Deed | Attribution 4.0 International | Creative Commons].

The assembly of FtsZ has also been probed in single-phase systems containing two crowders,^378^ as a closer approximation of the bacterial cytoplasm, in which crowding effects arise from various macromolecules with different properties rather than from one type of macromolecule. This is one of the few studies available on mixed macromolecular crowding, aimed at discerning whether these mixtures display additive or nonadditive behavior. The *Cc* of FtsZ assembly in these mixtures is always lower than that in dilute solution, but the effects generally deviate from the plain sum of those exerted by the individual crowders, and either reinforcement or counteraction of each other’s effects occur, depending on their physicochemical properties (Figure 18). Thus, the dramatic effects of negatively charged ovomucoid are strongly potentiated by Ficoll or dextran but counteracted by positively charged RNase A or by the osmolyte sucrose.

##### 6.3.1.4 Crowding and Other Division Proteins from *E. coli*

In addition to the above-described analysis of crowding effects on the Min system reconstituted on supported lipid bilayers alongside FtsZ and ZipA,^388^ two other crowding studies have focused on this oscillating protein complex. The first study used it as a model system to evaluate the ability of a new multicompartmental reaction-diffusion modeling method, *Spatiocyte*, to reproduce the effects of volume exclusion associated with crowding.^397^ This method is applied for the simulation of MinD translational diffusion in a crowded compartment with a 34% volume occupancy and also on a crowded surface with 23% of the area occupied with inert and immobile crowder molecules. Anomalous diffusion is observed in both cases, more pronounced on the crowded surface despite the lower occupancy, which is attributed to the lower dimensionality of the surface space. The results agree with previous studies suggesting that crowding on the cell membrane reduces diffusion of MinD and MinE.^398^ The second study investigates the impact of sucrose as a crowding agent on MinE amyloid-like structures involving its N-terminal domain. Lateral bending of the protein fibrils on mica surfaces seems to be modulated by crowding and ionic strength, according to AFM imaging.^399^

#### 6.3.2 LLPS and Cell Division

**Figure 19:**
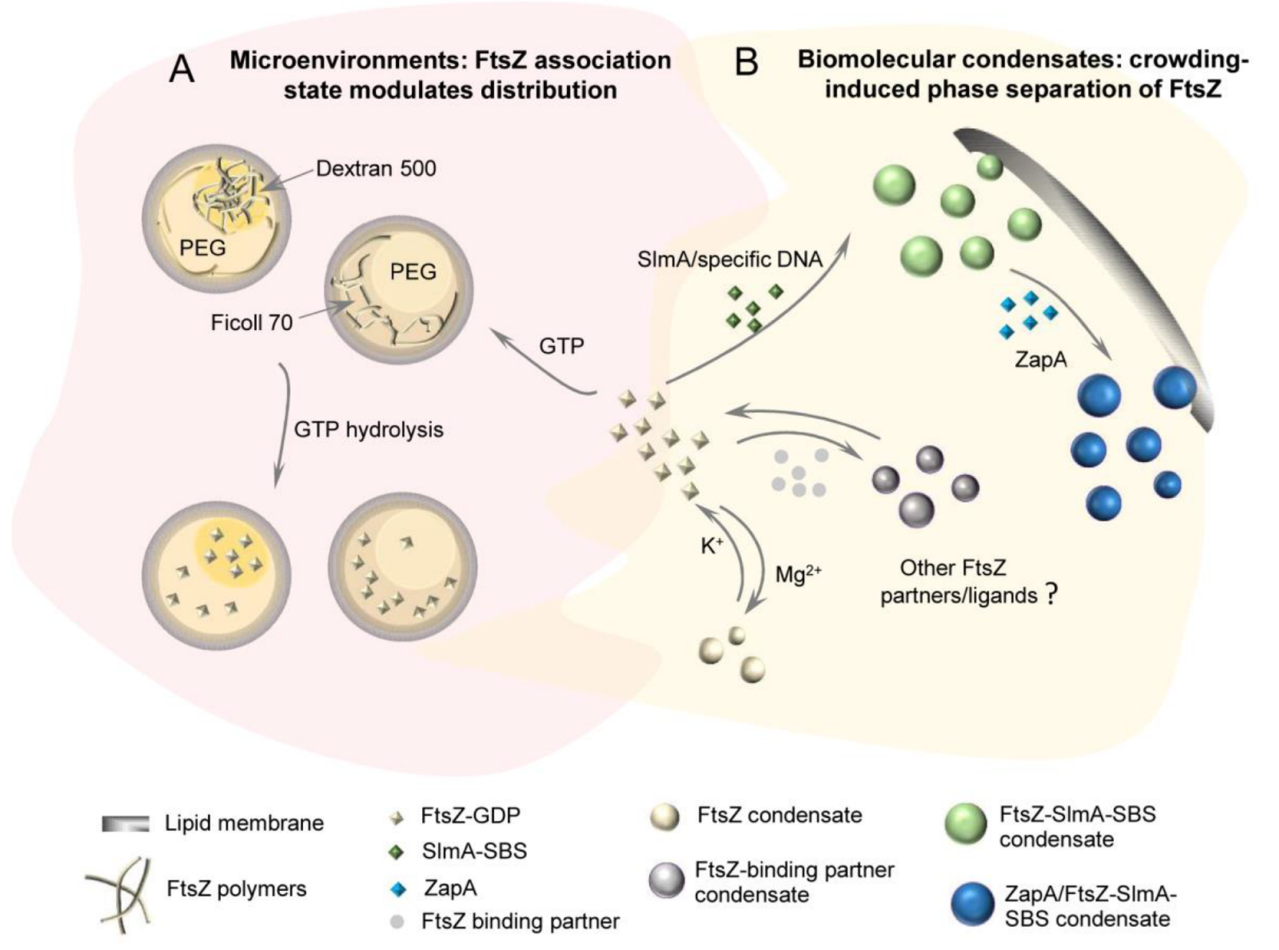
**FtsZ and phase separation.** (A) FtsZ distributes differently in encapsulated phase-separated binary mixtures of crowders (PEG/dextran 500 and PEG/Ficoll 70 are shown as examples) depending on its association state. Dissociation of polymers upon GTP depletion produces redistribution within phases of FtsZ species, that are no longer found at the lipid membrane confining the microdroplets.^401^ (B) Under crowding conditions promoting phase separation, FtsZ, alone or in the presence of binding partners, forms biomolecular condensates that congregate at the lipid boundary depending on their composition.^247,248^

As with the crowding reports, most studies of LLPS involving bacterial division proteins have been focused on *E. coli* FtsZ oligomers, polymers, and multiprotein or nucleoprotein complexes with partners. These works address two LLPS-related phenomena: the behavior of FtsZ in model crowding systems displaying aqueous two-phase behavior, and assembly of FtsZ into homotypic and heterotypic phase-separated biomolecular condensates driven by crowding^400^ (Figure 19). There is also a large body of research on the phase separation of proteins involved in the regulation of asymmetric division in *C. crescentus*.

##### 6.3.2.1 LLPS and *E. coli* FtsZ

**Figure 20:**
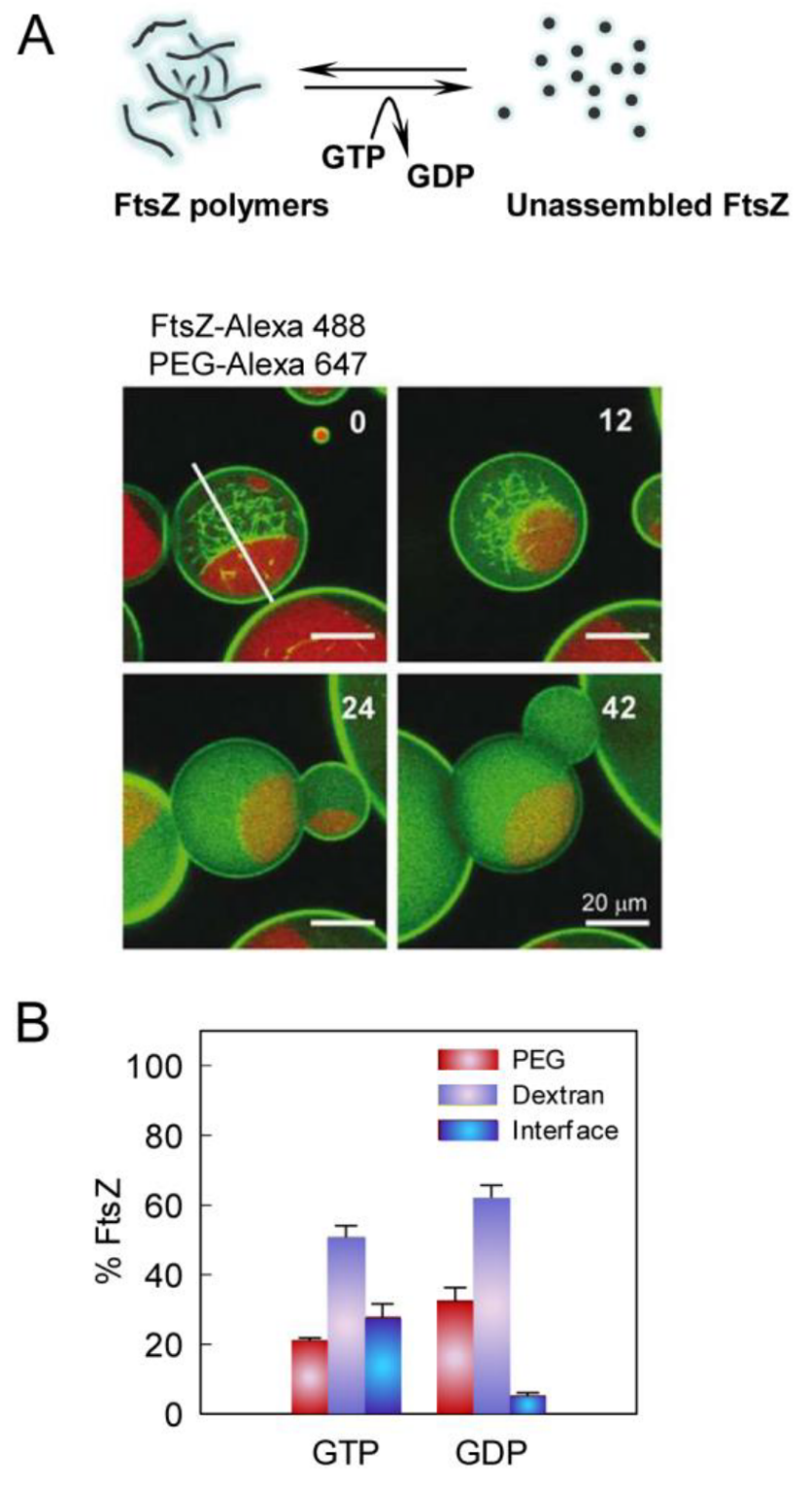
**Dynamic relocation of the bacterial division protein FtsZ as a function of its polymerization state in two-phase systems encapsulated inside lipid-stabilized microdroplets.** (A) FtsZ filaments preferentially locate in the dextran phase and at the interface of the dextran/PEG system. Upon GTP depletion the filaments disassemble and the protein partitions principally into the dextran phase with no obvious accumulation at the interface. Numbers in the confocal images correspond to time in minutes. A scheme of the association reactions of FtsZ is shown above. (B) Relative amount of FtsZ in each of the phases and at the interface obtained from fluorescence measurements. Reprinted in part from ref (401). Copyright 2016 the Authors. Published by Springer Nature under a Creative Commons Attribution 4.0 International License [CC BY 4.0 Deed | Attribution 4.0 International | Creative Commons].

The possible impact of the membraneless microenvironments inherent to all kinds of cells, including bacteria, on the reactivity and distribution of FtsZ has been analyzed in binary mixtures of PEG and a second crowder^401^ (Figure 19). Determinations by fluorescence of the partition coefficient (*K*) of FtsZ used the following equation:
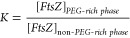
8where the fraction terms are the protein concentrations in both phases. The results show that FtsZ unevenly distributes in systems with two crowders at concentrations at which they demix, forming two compartments with distinct physicochemical properties. Confocal images of the samples are also in line with this observation (Figure 20). *K*-values < 1 are always obtained, meaning that FtsZ species are generally excluded from the more hydrophobic PEG phase, in which denatured proteins usually partition because of the exposure of their hydrophobic amino acid residues.^402^ FtsZ strongly accumulates in Ficoll 70 or DNA phases, reflected by *K*-values < 0.2, while *K*-values around 0.5 were obtained in LLPS systems with dextran 500, indicating a lower preference for this phase. Being both inert crowders, differences in FtsZ partition between Ficoll 70 and dextran 500 could be ascribed to their different properties rather than their size, as similar partitions are found in dextrans 500 and T40. The asymmetric distribution of FtsZ suggests that microenvironments could contribute to the spatial regulation of FtsZ assembly, facilitated in areas where the protein accumulates above the *Cc* and hindered in regions of insufficient protein concentration.

A significant fraction of the protein locates at the interface of the dextran/PEG compartments when polymers are triggered by GTP.^401^ This interfacial localization, often observed for large particles because of the concomitant reduction of interfacial tension,^403^ might serve to concentrate the FtsZ polymers within a defined region and to organize them in two dimensions, perhaps rendering a relative orientation more suitable for constriction than the arrangements in three dimensions. Moreover, the distribution of FtsZ in these systems seems to respond dynamically to the self-association state of the protein, which shifts from one location to another in response to GTP addition and depletion. This has been verified by encapsulation of the LLPS system within water-in-oil microdroplets stabilized by lipid membranes, which provide a more stable platform than the bulk phases (Figure 20). These cell mimics can be generated by manual emulsion, rendering a multiplicity of containers of different sizes^401^ which may be advantageous in some instances, as with size-associated phenotypes,^404^ or in a more controlled manner by microfluidics microdroplets with the exact same size and composition.^405^

**Figure 21:**
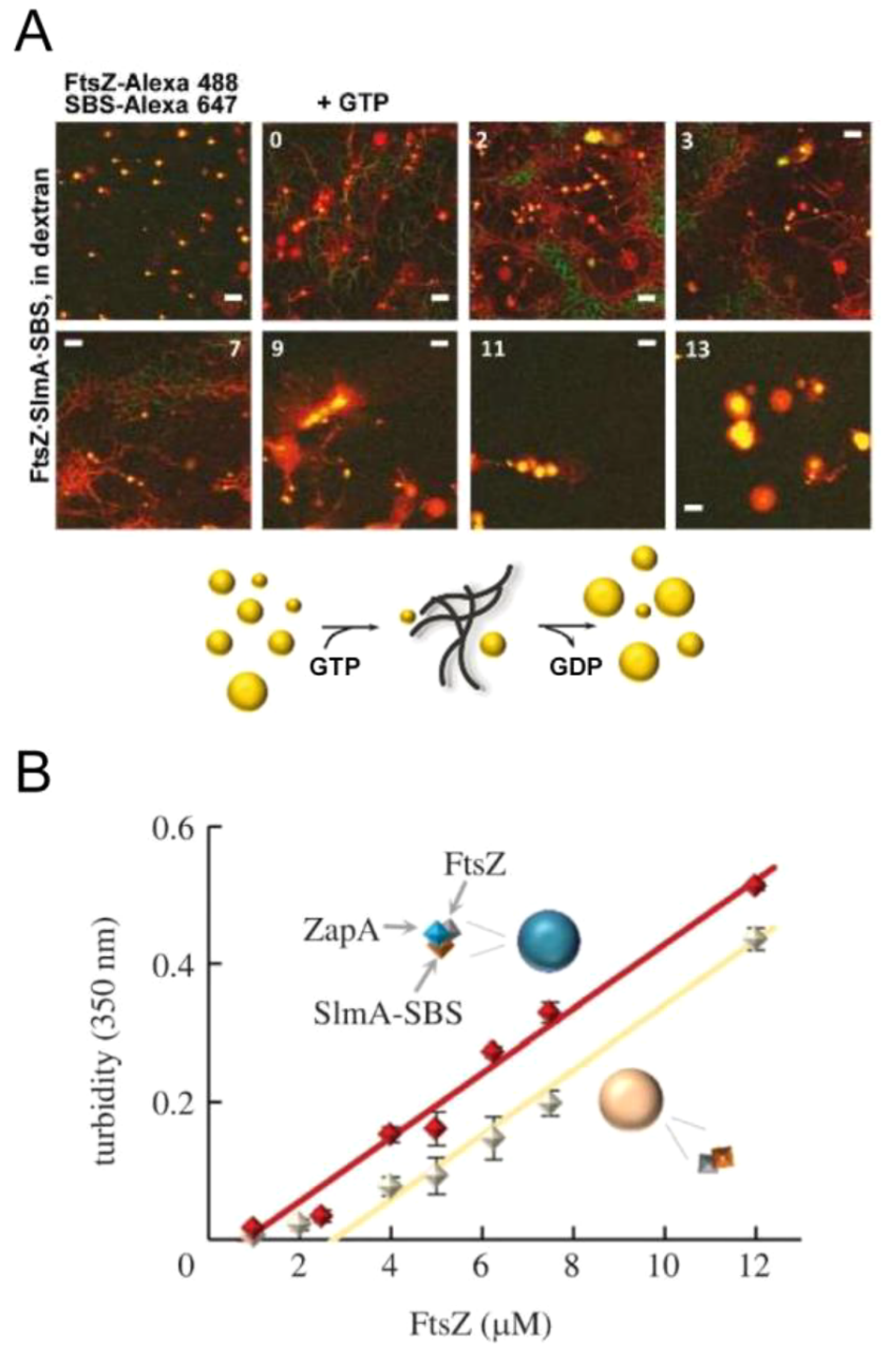
**Dynamic FtsZ-SlmA-SBS condensates in crowded media.** (A) Assembly of GTP-triggered FtsZ polymers after addition of nucleotide to FtsZ-SlmA-SBS condensates. The number of condensates decreases with polymer formation. After disassembly of the polymers due to GTP exhaustion, condensates reassemble. Times are in minutes (time zero, GTP addition). Scale bars: 5 μm. A scheme of the dynamic process is shown below. Reprinted in part with permission from ref (248). Copyright 2018 the Authors. (B) Incorporation of ZapA slightly decreases the *c*_sat_ of condensation of FtsZ-SlmA-SBS, monitored using turbidity. Reprinted in part from ref (247). Copyright 2023 the Authors. Published by the Royal Society under the terms of the Creative Commons Attribution License [http://creativecommons.org/licenses/by/4.0/].

FtsZ was later found to self-assemble into biomolecular condensates arising from phase separation facilitated by crowding^248,255^ (Figure 19). Indeed, FtsZ is a good candidate for LLPS because it contains an IDR that flexibly links the globular core polymerization domain and the CCTD^406,407^ and exhibits homo- and heteroassociations that confer multivalency.^375,380^ Addition of SlmA/SBS to FtsZ in the absence of GTP results in dynamic structures enriched in both proteins and SBS DNA that display characteristics of liquid-like condensates^248^ (Figure 21). Biomolecular condensation is favored by the additional multivalency conferred by the FtsZ/SlmA/SBS system. Notably, SlmA dimerizes and forms SlmA-SBS complexes with a 4:1 stoichiometry under conditions at which FtsZ oligomerization is insufficient to drive its own phase separation. Homotypic FtsZ condensates can be detected only at lower salt, higher Mg^2+^ and crowder concentrations.^255^ The intrinsically disordered linker sequence of FtsZ is not essential for condensation in this case, because its removal still permits condensation with similar *c*_sat_ (protein concentration at which condensates start assembling) to that for the full-length protein, as measured by turbidity. Nonetheless, the linker does play a role in condensate assembly kinetics.^255^

Homotypic FtsZ condensates and heterotypic FtsZ/SlmA/SBS condensates recruit the division ring regulator ZapA, an agonist of Z-ring formation that does not display condensation on its own and, contrary to SlmA/SBS, does not promote condensation of FtsZ under conditions disfavoring its oligomerization.^247^ This regulator shows a minimal reduction in the apparent *c*_sat_ of formation of the FtsZ/SlmA/SBS condensates (Figure 21). Determination of *c*_sat_ for condensates involving more than one macromolecule is not straightforward^408^ and, in this case, an apparent value is obtained from turbidity measurements in which the ratio between the three elements is kept constant and the total concentration increased.^247^

Perhaps the most noteworthy feature of FtsZ condensates *in vitro* is their ability to interconvert with FtsZ polymers when GTP is added, followed by condensate reassembly after GTP depletion due to FtsZ’s GTPase activity. The prevalence of condensates or polymers depends, therefore, on the nucleotide present and is also subject to regulation, with SlmA/SBS strongly favoring condensates vs polymers, and ZapA favoring polymers.^247^ This suggests that, *in vivo*, condensates may prevent FtsZ assembly into the bundles that are normally competent for Z-ring assembly at noncentral areas of the cell or under nongrowing conditions, when GTP levels are low. Similarly, accumulation of positive regulators at the cell center could rescue FtsZ from the condensates, favoring the assembly into polymers and, hence, Z-ring formation at midcell.

**Figure 22:**
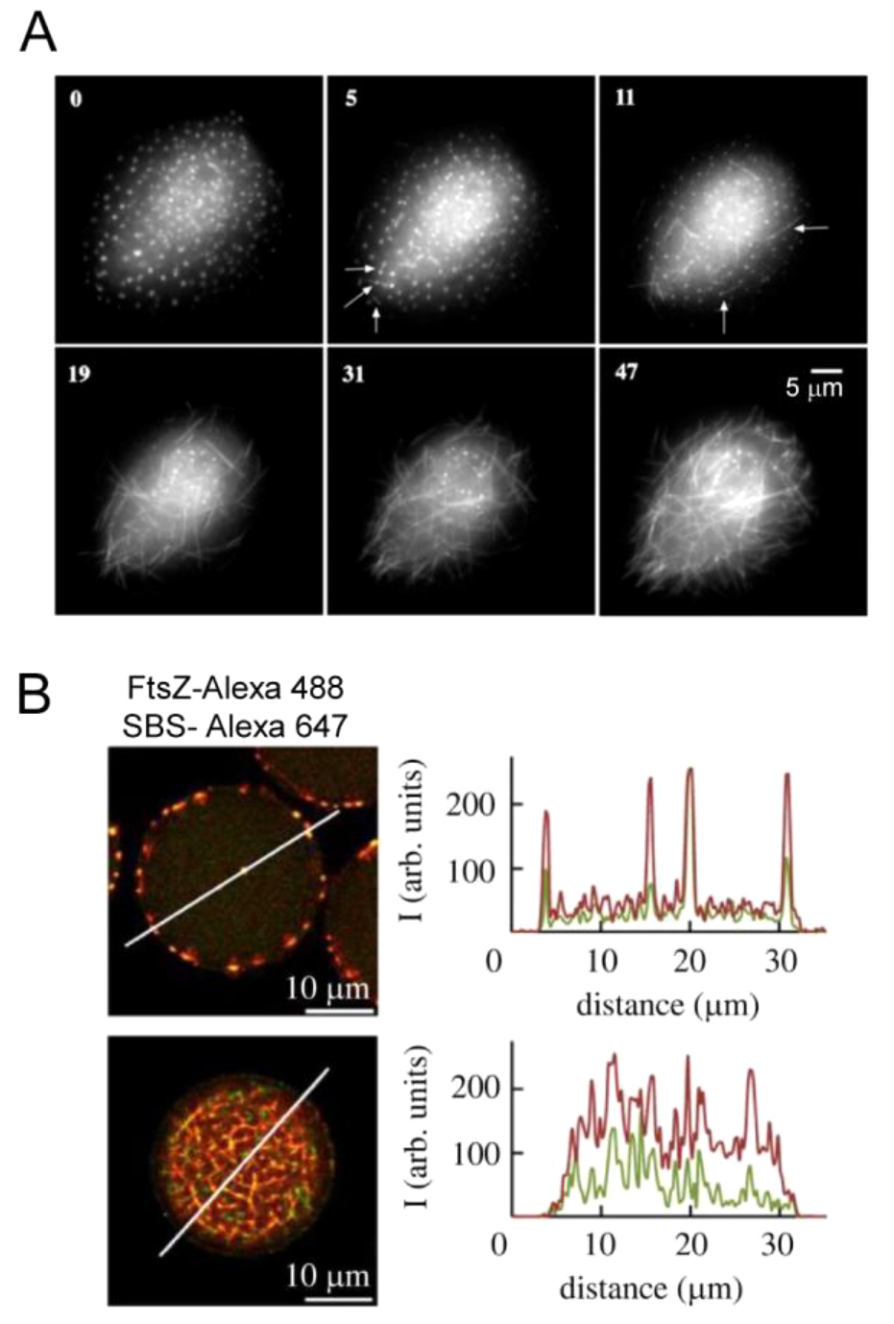
***E. coli*****FtsZ foci suggestive of condensates.** (A) FtsZ-GFP foci and filament formation in Chinese hamster ovary cells, after treatment with vinblastine. FtsZ-GFP localization is shown in the same living cell at various times after addition of the drug, in minutes. Arrows indicate growth of filaments from the foci at random locations in the cytoplasm. With time, filaments grow longer, forming a network of filaments, while foci disappear except in the nucleus. Reprinted with permission from ref (409). Copyright 1999 The Company of Biologists Ltd. (B) Representative confocal images of FtsZ-SlmA-SBS condensates (top) and GTP-triggered polymers (bottom) in lipid-stabilized microfluidics-based microdroplets. Also shown are the intensity profiles of the green and red channels, obtained along the line drawn in the images. Reprinted in part from ref (247). Copyright 2023 the Authors. Published by the Royal Society under the terms of the Creative Commons Attribution License [http://creativecommons.org/licenses/by/4.0/].

Interestingly, heterotypic FtsZ/SlmA/SBS condensates preferentially locate at the membrane when reconstituted inside microdroplets generated by microfluidics that display crowding and compartmentalization as cell mimics^248^ (see section 4; Figure 22). This behavior is consistent with the tendency of SlmA to bind to membranes^252^ and is also in line with the known enhancement of condensation by surface effects.^51^ The influence of lipid membranes on the formation of these biomolecular condensates has been further analyzed using supported lipid bilayers as minimal membrane systems in buffers containing glutamate, the most abundant anion in *E. coli*, which favors formation of condensates of large size.^254^

In light of the current body of knowledge about LLPS behavior, it is likely that the formation of FtsZ condensates has been previously overlooked in *in vitro* and *in vivo* studies.^247^ Indeed, structures compatible with condensates can be observed in images taken upon disassembly of FtsZ polymers reconstituted alongside SlmA/SBS in GUVs, long before they were described as such.^391^ Similarly, expression of *E. coli* FtsZ in mammalian cells resulted in formation of dozens of round foci throughout the cytosol that disassembled upon addition of vinblastine, an antitubulin drug, leading to FtsZ polymer assembly^409^ (Figure 22). FtsZ condensates in bacterial cells have not yet been confirmed, but *E. coli* cells under long-term nutritional stress form polar foci containing FtsZ (and other divisome proteins) that convert back to polymers upon nutrient addition.^410^ These reversible foci, along with reversible foci of FtsZ during the nondividing portion of the *C. crescentus* cell cycle that convert to polymers prior to cell division,^411^ require further study but are suggestive of condensates.

##### 6.3.2.2 LLPS and *B. subtilis* Noc Protein

Like SlmA in *E. coli*, the Noc protein mediates nucleoid occlusion in the Gram-positive species *B. subtilis.*(412) Unlike SlmA, Noc does not seem to interact with FtsZ directly and instead inhibits FtsZ migration away from the midcell FtsZ ring.^413^ Intriguingly, however, Noc shares with SlmA the ability to form biomolecular condensates, which have been characterized through reconstitution in GUVs and supported lipid bilayers.^147^ Phase separation of Noc scales with its concentration, and it is sensitive to the type of salt present in the solution, being favored by potassium glutamate and inhibited by KCl and NaCl. In addition, as observed for other proteins in the DNA-binding ParB family, Noc condensates are strongly promoted by the nucleotide CTP, also known to regulate its membrane binding activity.^414^ Indeed, these condensates bind to the lipid membrane of water-in-oil microdroplets and GUVs, where they form either film-like structures or round 3D-condensates depending on the protein concentration. Noc condensates induce membrane deformations and preferentially bind to the liquid-disordered phase domains in GUVs exhibiting different membrane domains. Deformation of lipid membranes has also been found in other phase separated systems (see section 4). One interesting observation is that Noc condensates recruit FtsZ, whether the latter is membrane-bound through a membrane targeting sequence or not, despite the lack of any known direct interaction between these proteins. This is probably because of the enhanced concentration of Noc within these condensates, which might potentiate possible weak interactions with FtsZ. Round structures resembling condensates are observed in images taken *in vivo* in prior work where the interaction of Noc with the membrane was revealed.^251^

##### 6.3.2.3 LLPS and *C. crescentus* Cell Division Proteins

**Figure 23:**
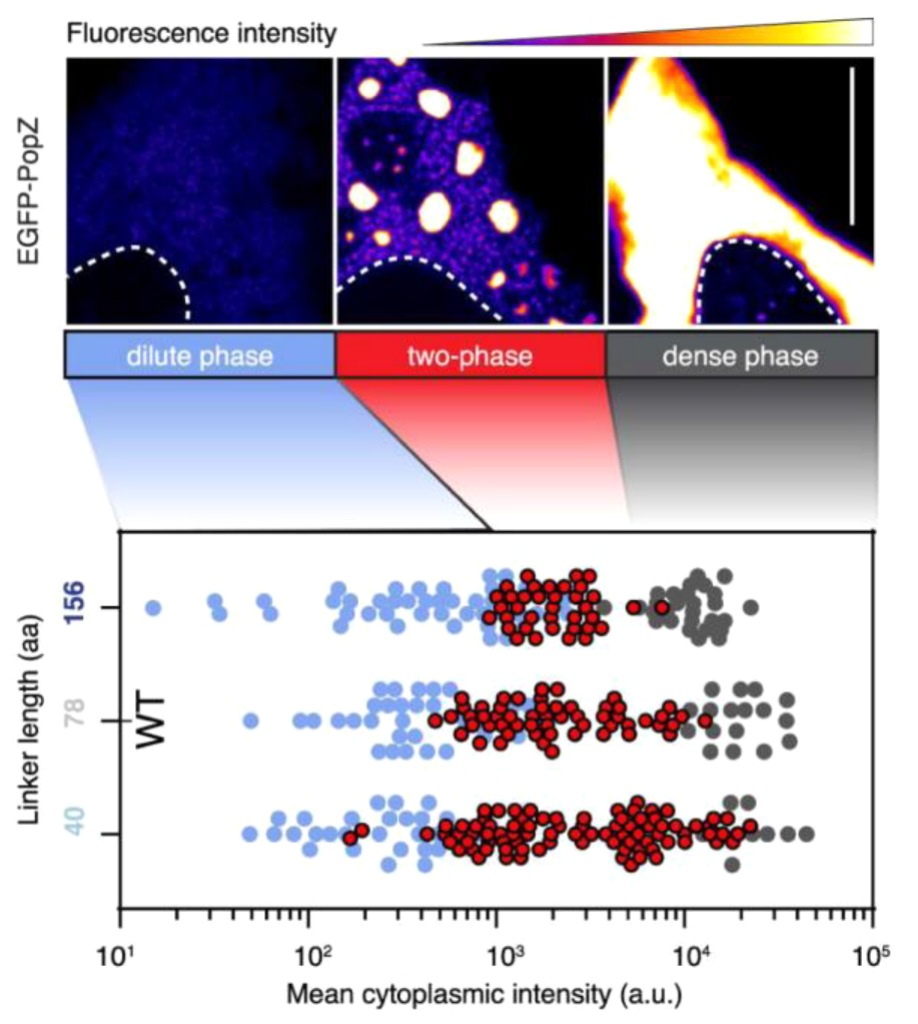
**PopZ condensates are regulated by PopZ structural features.** Shown at the top are phase diagrams of PopZ expressed in mammalian cells, with PopZ in a dilute phase, two phases, or a dense phase. The nucleoid boundary is represented as a white dotted line. Scale bar, 10 μm. Shown below are phase diagrams of EGFP fused to three PopZ variants with different linker lengths. Each dot represents data from a single cell, and dot color indicates phase. Figure reprinted in part from ref (146). Copyright 2022 the Authors. Published by Springer Nature under a Creative Commons Attribution 4.0 International License [http://creativecommons.org/licenses/by/4.0/].

PopZ, an intrinsically disordered, oligomerizing protein involved in the cell division of the model Gram-negative species *C. crescentus*, forms a large biomolecular condensate in the cytoplasm at one cell pole. In addition to their characterization in *C. crescentus* cells, PopZ condensates have been analyzed *in vitro* in the presence of divalent cations and after expression in mammalian cells.^146^ As in other instances, e.g. the eukaryotic stress sensor Pab1^415^ and *E. coli* FtsZ,^255^ condensation is not driven by IDRs of PopZ but by folded regions within the oligomerization domain. Nevertheless, as with FtsZ, the IDRs of PopZ contribute to the regulation of the process. The *c*_sat_ and *c*_D_ thresholds for the two-phase and the single-dense-phase regimes, respectively,^36^ and the dynamics of the condensates depend on the length of the unstructured sequence (Figure 23). These key parameters defining condensation behavior could also be tuned by the degree of multivalency of the C-terminal helical region. Interestingly, intrinsically disordered proteins are less common in bacteria compared with eukaryotes (see section 5.3).^416,417^

Alterations in the fluidity of the natural PopZ condensates change their cellular localization and ability to recruit regulatory proteins, implying that modified condensates are often unable to fulfill their role in the orchestration of asymmetric division, which compromises cellular fitness. Notably, not only solid-like but also PopZ condensates that are too liquid are not perfectly suited for their function. From a synthetic biology standpoint, a recent study^146^ nicely illustrates how synthetic condensates can be rationally designed by dissection of the molecular grammar driving their formation, enabling applications of these structures in biotechnology and biomedicine. It is also proposed that thorough analysis of the material properties of condensates could help us to understand their role in pathologies such as neurodegeneration, mediated by the formation of solid aggregates of proteins like FUS. Another interesting aspect of this study is that the PopZ condensates assembled within mammalian cells are larger than those occurring in their native bacterial cells. These larger condensates retain their intrinsic properties such as the specific partitioning of bacterial proteins and their dynamics, as measured by FRAP. This suggests that mammalian cells, like the microdroplets or GUVs used in other studies, may serve as convenient platforms to reconstitute biomolecular condensates of bacterial origin in order to facilitate their analysis. This also brings up the question of whether components within the crowded cytoplasm of bacteria, such as ribosomes, limit the size of these condensates in the cytoplasm of bacterial cells compared with the eukaryotic cell cytosol.

**Figure 24:**
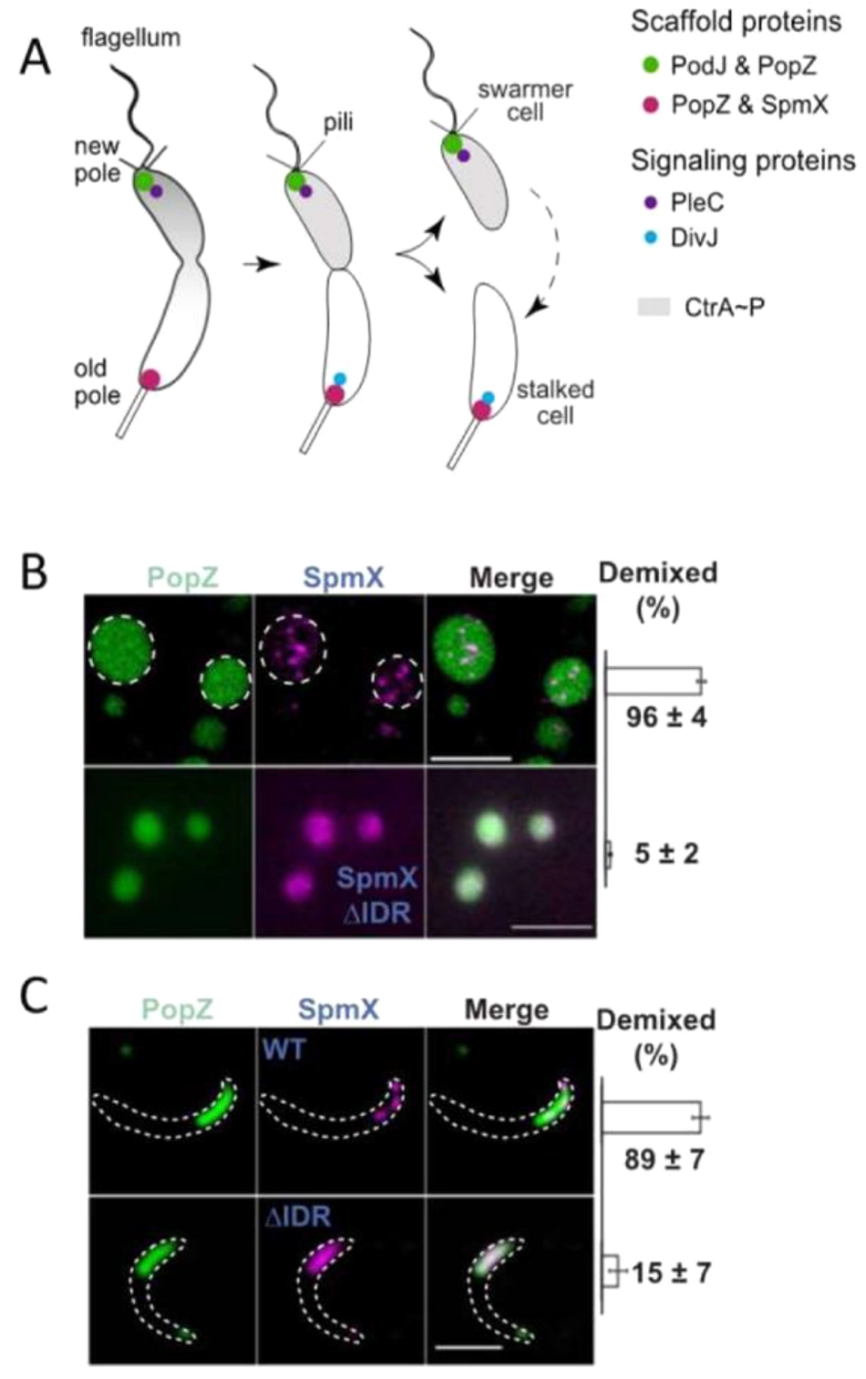
**Biomolecular condensates of the proteins SpmX and PopZ from *C. crescentus*.** (A) Localization of PopZ and its associated scaffold proteins PodJ and SpmX, and signaling proteins PleC and DivJ, at specific cell poles of *C. crescentus* before and after cell division. SpmX recruits DivJ to condensates at the old pole and stimulates the latter’s kinase activity. CtrA-phosphate is a master transcriptional regulator that controls expression of multiple *C. crescentus* genes and is selectively enriched at the new cell pole. Figure reproduced from ref (249). Copyright 2022 the Authors. Published by Springer Nature under a Creative Commons Attribution 4.0 International License [http://creativecommons.org/licenses/by/4.0/]. (B) Super-resolution images of purified PopZ (labeled with Atto488) and SpmX (ΔTM, labeled with Cy3) with (top) or without its IDR (bottom), showing demixing of the condensates of SpmX within the condensates of PopZ *in vitro*, driven by the IDR. (C) False-colored images of *C. crescentus* cells expressing mCherry-PopZ (green) and SpmX-dL5 (magenta) with (top) or without the SpmX IDR (bottom), suggesting that wild-type SpmX forms multiple condensates in the PopZ microdomain *in vivo*, also promoted by the IDR. The percentage of PopZ condensates enclosing more than one SpmX condensate (B) or cells with more than one SpmX cluster in the PopZ microdomain (C) is indicated on the right. Scale bars, 5 μm. Figure adapted from ref (150). Copyright 2022 the Authors. Published by American Association for the Advancement of Science under a Creative Commons Attribution License 4.0 (CC BY) [https://creativecommons.org/licenses/by/4.0/].

Other proteins involved in the asymmetric division of *C. crescentus* have also been described to form biomolecular condensates. For example, SpmX, an integral membrane protein, directly interacts with PopZ at the pole of *C. crescentus* cells opposite from the FtsZ focus mentioned above. This PopZ-SpmX condensate recruits the cell division protein DivJ to the polar microdomain, stimulating its kinase activity.^150^ Although SpmX and PopZ form condensates at the same cellular location, they are demixed, forming distinct zones within the condensate (Figure 24). Multivalent interactions between these two proteins are modulated by protein concentrations, temperature, salt, and nutrients. Interestingly, ATP concentrations in the low millimolar range, which occur when nutrients are plentiful, dissolve the condensates of SpmX or PopZ. This behavior is consistent with the previously described role of this nucleotide as a hydrotrope.^198^ Surprisingly, while SpmX condensation is inhibited by 1,6-hexanediol (1,6-HD), that of PopZ is promoted, despite its demonstrated biomolecular condensation properties.^146^ This constitutes a good example of the limitations of 1,6-HD to assess biomolecular condensation behavior, as previously discussed.^36^ In contrast to the dissolution of condensates with ATP, depletion of ATP promotes condensation of the SpmX disordered domain. This leads to compartments with DivJ at higher concentrations, which in turn enhances its activity when its substrate is scarce, for example under low glucose conditions.

Interestingly, SpmX acts as a negative regulator of phase separation by PodJ, another membrane protein involved in the regulation of cell division,^249^ and this behavior could have profound implications for the regulation of the cell cycle of *C. crescentus*. *In vitro*, biomolecular condensates of PodJ, driven by its disordered domains and coiled-coils, are assembled at relatively low protein concentrations, and they are highly regulated by salt. Below 100 mM NaCl, irreversible structures are observed, whereas above this concentration, liquid droplets form and high salt concentrations dissolve them. Biomolecular condensates of PodJ have also been detected *in vivo*, and they are less fluid compared with those assembled *in vitro*. Macromolecular crowding and the cell membrane to which PodJ is tethered are among the factors invoked to explain this difference in fluidity. In fact, as mentioned, macromolecular crowding has been described to play a key role in the assembly of biomolecular condensates,^35^ including those of the bacterial cell division proteins from *E. coli.* Hence, it would not be surprising that the condensates from *C. crescentus* proteins are also affected by crowding.

The inhibition of PodJ phase separation and cell pole targeting by SpmX could have a role in the clearance of PodJ at the old cell pole, since SpmX is expressed after the formation of the PodJ condensates. Moreover, these condensates act as hubs that accumulate client-signaling factors through interaction with different regions of the protein, such as the histidine kinase PleC. Recruitment of PleC by PodJ condensates inhibits PleC activity, suggesting another way that phase separation, in conjunction with allosteric mechanisms, could contribute to the regulation of enzymatic activity in bacteria.^418^ Some of these studies were conducted by heterologous expression of the proteins of interest in *E. coli*, exploiting the lack of homologues of *C. crescentus* polarity proteins in this organism.

## 7 Connections between Phase Separation and Bacterial Fitness

Since the discovery that bacterial proteins are also able to assemble into biomolecular condensates arising from phase separation, it has become clear that one potential role of these structures is to protect bacterial cells from stressful conditions. For example, the first bacterial condensates identified *in vivo*, the bacterial ribonucleoprotein bodies (BR-bodies) from *C. crescentus* assembled by RNase E^276^ and mRNA-dense bodies at the poles of *L. lactis,*(185) are thought to be analogous to eukaryotic P-bodies and stress granules. RNase E is crucial for mRNA degradation, and it has been hypothesized that its phase separation, promoted by RNA and reverted by its cleavage, might accelerate mRNA degradation. Typical of many condensate-forming proteins, RNase E harbors an IDR that is necessary and sufficient for its LLPS. *In vitro*, phase diagrams show that this condensation depends on protein concentration and ionic strength. Cells respond to cellular stress (EDTA or ethanol treatment, or heat shock) by forming BR-bodies, which are subsequently dissolved upon removal of the stress. The BR-bodies increase stress tolerance and overall fitness, as disruption of the RNase E disordered region and inhibition of condensate formation lead to higher susceptibility to stresses.^276^ Such effects are seemingly not related to the ability of the BR-bodies to recruit degradosome components. The presence of the aberrant polar mRNA foci in *L. lactis* correlates with cessation of cell division, a heat shock response and loss of nucleoid-occluded ribosomes. The mRNA dense bodies accumulate when transcripts are formed that encode poorly produced membrane proteins, suggesting defects in the coupling of transcription, translation, and membrane insertion.

More recently, it has been proposed that biomolecular condensates called aggresomes increase bacterial fitness, enabling cells to survive stresses such as antibiotic treatment, starvation, oxidative stress, heat shock, or phage infection.^149,185^ These structures can be found in *E. coli* but also in other Gram-negatives, and they accumulate proteins such as HslU, a component of the HslVU protease, Kbl, an enzyme that degrades threonine as part of the serine biosynthetic pathway, and AcnB, a *cis*-aconitase involved in central metabolism. Cells with aggresomes are more resistant to stress because these structures sequester proteins vital for cellular function, thereby shutting down their associated processes and forcing the cell into a dormant state.^419,420^ This state correlates with a marked change in the physical properties of the cytoplasm, which changes from a fluid state to a more glass-like state.^155^ Biomolecular condensation is indeed emerging as one of the possible mechanisms behind the intriguing formation of dormant and persister cells in bacterial populations,^421^ which are able to withstand stresses such as antibiotic treatment, hence representing a threat to human health. Cellular ATP levels decrease markedly upon entry into the stationary phase as a result of the decrease in cellular energy levels. This ATP depletion favors the formation of aggresomes, consistent with ATP acting as a hydrotrope that dissolves aggregates and biomolecular condensates.^198^ These condensates are heterogeneous in composition and physical properties, and it is hypothesized that these properties might be tuned to respond to stresses of different intensities and durations. Similar to BR-bodies and many other dynamic biomolecular condensates,^276^ aggresomes form under stress conditions and disassemble when the stress is over. In a recent study of bacterial dormancy in response to antibiotic exposure, faster rates of aggresome disassembly correlated with shorter lag times for cells to exit the dormant state and regrow.^422^

LLPS also seems to play a role specifically in the protection of bacterial DNA from damage due to stressful conditions such as exposure to UV light. For example, biomolecular condensates enriched in the (ss)DNA-binding protein SSB serve to sequester excess levels of this protein alongside its interacting partners near the membrane.^253^ Early reports show that SSB levels largely exceed those required to cover the ssDNA sites during replication.^423^ Storage of this excess in phase-separated compartments would facilitate rapid mobilization of the SSB protein pool when necessary to protect the exposed ssDNA and repair damaged genome loci, as the increase in ssDNA sites dissolves the condensates. In support to this model, cells with mutant SSB unable to efficiently phase separate but still able to bind ssDNA are viable in stress-free conditions but more sensitive to UV light damage than wild-type cells.^424^

**Figure 25:**
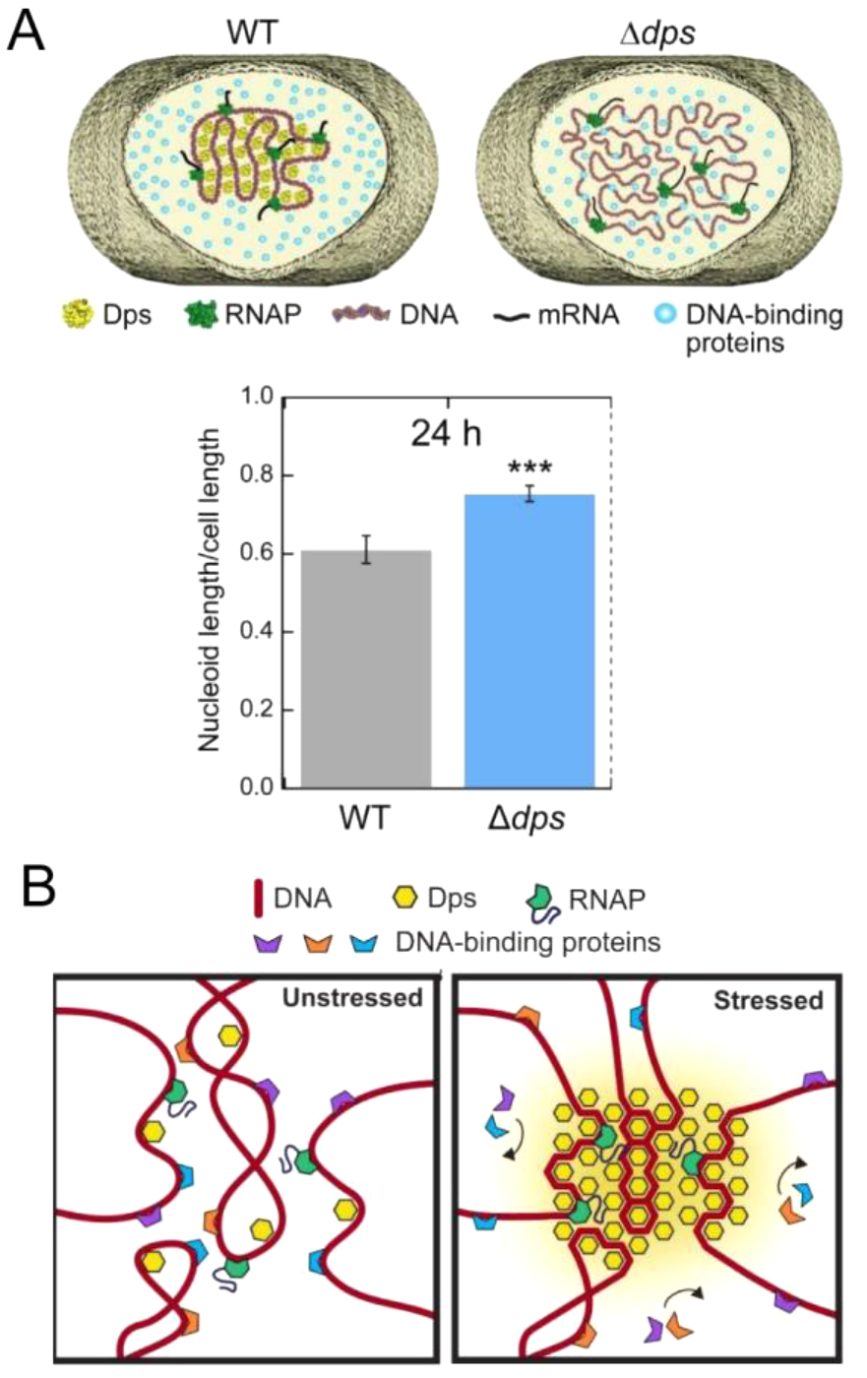
**Protection of the DNA by Dps under stress conditions.** (A) In wild-type cells, Dps condenses the DNA during the stationary phase (left). This condensation does not take place in the absence of Dps (right). Below, ratios of nucleoid length to cell length in cells with and without Dps. (B) Schematic representation of the model proposed for the protection of DNA by Dps. In the absence of stress conditions, Dps binds to DNA but no major condensation of the nucleoid occurs (left). Under stress conditions, Dps forms biomolecular condensates on a large part of the nucleoid into which RNAP can freely diffuse while other proteins are excluded, which blocks their access to the DNA (right). Figure adapted with permission from ref (425). Copyright 2018 Elsevier Inc.

Biomolecular condensation of the DNA protection protein Dps shields DNA under stress conditions by its compaction into a dense complex, also acting as a global regulator of transcription^226,425^ (Figure 25). Dps condensates do not prevent binding of RNAP, which has access to buried genes, but exclude some other DNA-binding proteins like restriction enzymes, the activity of which decreases with increasing Dps. Upregulation of Dps may also ensure that transcription can continue under conditions of extreme stress. Indeed, Dps deletion reduces survival rates over a diverse range of stress conditions (e.g., heat shock, osmotic shock, starvation, UV exposure, antibiotics, and oxidative stress). Intracellular Dps levels are specifically regulated by the selective ATP-dependent protease ClpXP, which hydrolyzes Dps in the presence of glucose.^426^ Finally, other NAPs that can form phase separated condensates on DNA are the HU proteins^226^ (see section 3), implicated in stress response pathways such as the SOS and the osmolarity/supercoiling responses and in the environmental programming of the cellular response during aerobic and acid stress.^427^ Some NAPs provide an efficient response to various stress conditions, regulating transcription through condensation of the nucleoid.^207^

In contrast to eukaryotic cells, bacteria often have to survive in environments with highly varying nutrient availabilities and types. Biomolecular condensation has been proposed to concentrate enzymes present at low copy numbers, thus enhancing their activity under starving conditions. This is the case of the above-described condensation of *C. crescentus* SpmX, which at low ATP levels recruits the DivJ kinase as a client to the condensates.^150^ This recruitment of DivJ concentrates it and results in more efficient kinase activity when ATP levels are low, which is crucial in this aquatic species that often encounters low nutrient densities. Phase separation is also used by the commensal bacterium *Bacteroides thetaiotaomicron* to maintain fitness in the mammalian gut, a hostile environment with highly variable nutrient levels, multiple competitors, and threats posed by the host immune system.^428^ Nutrient starvation in this bacterium triggers phase separation of the transcription termination protein Rho, which is driven by its IDR and regulated by protein concentration, salt concentration, and RNA binding. The sequestration of Rho molecules into these membraneless compartments increases Rho transcription termination activity, which in turn modifies the RNA abundance of hundreds of genes, including several required for gut colonization, ultimately promoting bacterial fitness. Finally, photosynthetic cyanobacteria regulate the availability of metabolic enzymes during light–dark cycles by sequestering them in puncta at night and releasing them in a soluble form during the day.^429^

Other phase-separation related defenses against starvation involve nucleotides and polyphosphate (polyP). PolyP granules are constitutively assembled in some bacteria but also are often formed in response to nutrient limitation.^430^ In *Pseudomonas aeruginosa,* in conjunction with the universal starvation alarmone (p)ppGpp, polyP has an additive effect on the nucleoid dynamics and organization, protecting the chromosome during starvation, increasing fitness, and helping cells to survive stresses such as antibiotics. Signaling by (p)ppGpp downregulates enzymes involved in GTP biosynthesis in both *B. subtilis* and *E. coli*,^431,432^ and connections have been established between this signaling and persister cell formation.^433^ Persister cells display slow or arrested growth,^420^ and this may be related with their low GTP levels that would shift the equilibrium of FtsZ away from polymers and toward biomolecular condensates.^248^ Along these lines, foci containing folded FtsZ localize to cell poles in nongrowing late stationary phase *E. coli*, *Salmonella typhimurium*, and *Shigella flexneri* cells and are related to multidrug tolerance.^410^

## 8 Conclusions and Future Perspectives

Macromolecular crowding is a key element of the intracellular complexity that potentially modulates the protein–protein, protein–nucleic acid, and protein–lipid interactions in cells. Complexes or assemblies of molecules occurring in bacteria are particularly exposed to crowding effects, because the total concentration of macromolecules in the cytoplasm of these microorganisms is higher than in the cytosol of eukaryotic cells. Crowding in bacteria has been shown to promote the assembly of proteins into larger complexes, to facilitate the binding of proteins to nucleic acids, to stabilize the structure of macromolecules, and to modulate their activity. Macromolecular crowding also elicits the formation of distinct compartments by phase separation, which appears most relevant in the case of bacteria, as they generally lack the membrane-bound organelles that are so crucial for organizing the eukaryotic cytoplasm.

Evaluation of crowding effects on biomolecular interactions and the characterization of biomolecular condensates are technically challenging and further aggravated *in vivo* by the small size of bacterial cells. Fluorescence methods are among the most useful tools, because of their ability to specifically monitor the molecules of interest in the presence of crowding agents together with the temporal and spatial resolution they provide. The rapid development of fluorescence super-resolution imaging methods and the application of fluorescence microspectroscopy such as fluctuation approaches partially overcome the hurdles resulting from the small size of bacteria, allowing identification of biomolecular condensates and assessment of the dynamics and function of their hallmark components. An alternative to the cellular studies is the reconstitution of the macromolecules in model crowded systems, in bulk solution, or encapsulated within a lipid monolayer or bilayer. Compared with the studies in cells, this strategy allows evaluation of the system in more controlled conditions and a more straightforward interpretation of the results. However, performing quantitative measurements of interactions is still more complex in these reconstituted systems than in the typical dilute solutions.

The rapidly growing number of studies reporting bacterial biomolecular condensates emphasize their importance, but their precise role in bacterial physiology remains elusive. Nevertheless, they seem to potentially participate in the regulation of cell cycle processes, as some factors engaged in cell division, nucleoid replication, and segregation have been shown to undergo phase separation. Biomolecular condensates may therefore be part of a mechanism to provide spatial control of these and other essential processes, a role traditionally attributed principally to the membrane. Moreover, such subtle mechanisms would be particularly appropriate for bacterial cells, which need to rapidly adapt to changes in environmental conditions. There is solid evidence supporting the implications of biomolecular condensates in cellular fitness and protection against adverse environmental conditions.

Despite intensive research during the last years, there are still many unsolved questions concerning the structure, function, and regulation of biomolecular condensates in general and of those assembled by bacterial proteins in particular. Some of the outstanding questions are summarized in Box 1. An interesting aspect is the regulation of biomolecular condensate formation by nucleotides, with CTP often having an enhancing effect and ATP and GTP usually a negative impact, along with other physicochemical factors such as pH, ionic strength, and cosolvents (e.g., compatible solutes). Bacterial condensates are also regulated by supramolecular structures such as DNA, RNA, and the membrane, but the underlying mechanisms are far from well understood. Particularly interesting would be elucidating the role of the nucleoid surface in the modulation of protein phase separation in bacteria. There are studies pointing to post-translational modifications as possible regulators of phase separation, by analogy with eukaryotic condensates, but further studies are needed to evaluate the generality of these observations. The ultrastructure of condensates and the precise arrangement of the components within the condensates, especially in the case of heterotypic ones, is still enigmatic. It also needs to be defined if the stoichiometry of the complexes in dilute solution is maintained when they phase separate to form the condensates. Super-resolution imaging methods together with single-molecule diffusion to probe the dynamics of molecules within the subcompartments of the cell will surely help to answer these questions. In addition, the factors determining the size of these condensates, which in bacterial cells are necessarily smaller than in eukaryotic ones, remain elusive. We hypothesize that the nucleoid-free space of the cell may be a major determinant of condensate size. The cellular amount of the protein forming the condensates and the relative amounts of the different components, in the case of multicomponent condensates, will likely influence their final size.

Box 1 **Outstanding Questions**

- What are the functions of biomolecular condensates in prokaryotes?
- Do ribosomes influence the mobility of native proteins in the cytoplasm of bacteria, archaea, and endosymbionts?
- What is the relation between reaction rates, diffusion coefficient, and protein concentrations in the cytoplasm?
- What is the mechanistic basis for the fluidization of the cytoplasm under different metabolic conditions?
- What is the molecular basis for the differences in protein mobility at the old and new pole of the cell?
- Do bacteria (and archaea) age?
- What determines the compaction of the nucleoid, given the large differences in the amount of DNA per volume of cytoplasm?
- How do the physicochemical characteristics such as crowding and confinement in bacteria affect phase separation differently than in mammalian cells, and does this give unique functional opportunities to bacteria?
- What are the factors that determine the size of bacterial biomolecular condensates?
- How is condensate assembly regulated by nucleotides in bacteria?
- How are the different components arranged within heterotypic bacterial biomolecular condensates?
- Is the stoichiometry of heterocomplexes maintained when they phase separate to form condensates in bacteria?
- How does crowding affect bacterial biomolecular condensates triggered by other factors?

It is still puzzling why the material properties of condensates such as fluidity need to be maintained within a narrow range to ensure functionality, with deviations toward either lower or higher fluidity seemingly detrimental in the few examples thoroughly analyzed. For natively disordered protein domains, the length of the disordered regions appears to control important material properties of the condensates, but generic physicochemical factors likely also play a role. Although some bacterial biomolecular condensates seem to be driven by macromolecular crowding, the crowding effects in other cases, where condensation is triggered by factors such as ionic strength changes or membrane surfaces, remain to be addressed. It is likely that in many of these cases, crowding will decrease the concentrations of the proteins at which phase separation occurs. Finally, we predict that many structures previously described as foci, bodies, diffusion barriers, or clusters that participate in cell division, SOS response, volume regulation, toxin-antitoxin systems, development of persister cells, and many other cellular processes finally turn out to be biomolecular condensates when viewed in light of the current body of phase separation research.

## Terminology

**Amphitropic proteins**: bind weakly (reversibly) to membrane lipids, and this process regulates their function.
**Biomolecular condensates**: are membraneless organelles that form dynamic and compartmentalized structures inside cells and can be described as physical gels. They form through phase separation and can have a wide range of viscoelastic properties. Condensates can bring molecules together that need to interact or segregate molecules from others.
**Chaotropes**: are molecules such as urea, guanidinium, and iodide that disrupt the structure of water and biomolecules. They weaken the hydrogen bonding network, leading to increased disorder and reduced structure in the surrounding water molecules.
**Colloids**: are mixtures of two or more phases where one substance is dispersed evenly throughout another one. The nucleoid behaves as a colloidal system within the bacterial cytoplasm.
**Cytoplasm**: is the intracellular fluid plus membrane-bounded compartments surrounded by the plasma membrane.
**Cytosol**: is the aqueous portion of the cytoplasm without organelles. We use the term cytosol in the context of eukaryotic cells and the term cytoplasm to describe the intracellular fluid of bacteria and archaea, which typically do not have organelles.
**Density**: is the macromolecule weight per volume, the number density (macromolecule concentration), or volume density (volume fraction). This is not the same as macromolecular crowding, which is the increase in chemical activity due to the colloidal osmotic pressure difference.
**Hydrotropes**: increase the solubility of poorly soluble compounds by reducing the surface tension of water.
**Hyperstructures**: refer to organized assemblies of cellular macromolecules such as the replication machinery, ribosomes, cytoskeleton, and divisome.
**Inclusion bodies**: are intracellular (irreversible) aggregates and typically result from overexpression and misfolding of proteins.
**Intracellular bodies**: are specialized structures such as ribosomes or compartments such as the nucleoid or protein-bounded cages in the cytoplasm.
**Kosmotropes**: are small molecules such as betaine and K^+^ and Na^+^ ions that have a stabilizing effect on the structure of water and biomolecules. They promote the formation of hydrogen bonds and tend to increase the order and structure of the surrounding water molecules.
**Macromolecular crowding**: refers to the effects of excluded volume on the energetics and transport properties of macromolecules within a solution containing a high total volume fraction of macromolecules.
**Metabolons**: are multienzyme/protein complexes that work in close proximity to enhance the efficiency of metabolic reactions. Examples are the glycolysis and fatty acid synthase metabolons and the pyruvate dehydrogenase complex.
**Micro- and nanocompartments**: are protein-bounded structures that serve to sequester specific metabolic processes or enzymatic reactions. An example is the carboxysome that contains enzymes for CO_2_ fixation.
**Osmoprotectants**: (also known as compatible solutes) are small organic molecules that accumulate in cells to high levels (up to molar concentrations) without interfering negatively with metabolic activity. The high and regulated levels of osmoprotectants help maintain osmotic balance by increasing the internal osmotic pressure.
**Plasmolysis**: refers to the loss of water from cells in a hypertonic environment (hyperosmotic stress). At high osmotic stress (when the turgor has become zero) the cytoplasmic membrane will shrink away from the cell wall, which is known as plasmolysis.
**Polysomes**: are mRNAs loaded with multiple ribosomes, also known as polyribosomes.
**Turgor or turgor pressure**: is the hydrostatic pressure difference that balances the difference in internal and external osmolyte concentration (osmolarity).

## References

[ref1] Dewachter L.; Verstraeten N.; Fauvart M.; Michiels J. An Integrative View of Cell Cycle Control in Escherichia Coli. FEMS Microbiol. Rev. 2018, 42 (2), 116–136. 10.1093/femsre/fuy005. 29365084

[ref2] Luby-Phelps K. The Physical Chemistry of Cytoplasm and Its Influence on Cell Function: An Update. Mol. Biol. Cell 2013, 24 (17), 2593–2596. 10.1091/mbc.e12-08-0617. 23989722 PMC3756912

[ref3] Spitzer J.; Poolman B. The Role of Biomacromolecular Crowding, Ionic Strength, and Physicochemical Gradients in the Complexities of Life’s Emergence. Microbiol. Mol. Biol. Rev. 2009, 73 (2), 371–388. 10.1128/MMBR.00010-09. 19487732 PMC2698416

[ref4] Rivas G.; Minton A. P. Toward an Understanding of Biochemical Equilibria within Living Cells. Biophys. Rev. 2018, 10 (2), 241–253. 10.1007/s12551-017-0347-6. 29235084 PMC5899707

[ref5] Rivas G.; Minton A. P. Influence of Nonspecific Interactions on Protein Associations: Implications for Biochemistry In Vivo. Annu. Rev. Biochem. 2022, 91 (1), 321–351. 10.1146/annurev-biochem-040320-104151. 35287477

[ref6] Bonucci M.; Shu T.; Holt L. J. How It Feels in a Cell. Trends Cell Biol. 2023, 33, 924 10.1016/j.tcb.2023.05.002. 37286396 PMC10592589

[ref7] Zimmerman S. B.; Trach S. O. Estimation of Macromolecule Concentrations and Excluded Volume Effects for the Cytoplasm of Escherichia Coli. J. Mol. Biol. 1991, 222 (3), 599–620. 10.1016/0022-2836(91)90499-V. 1748995

[ref8] Vendeville A.; Larivière D.; Fourmentin E. An Inventory of the Bacterial Macromolecular Components and Their Spatial Organization. FEMS Microbiol. Rev. 2011, 35 (2), 395–414. 10.1111/j.1574-6976.2010.00254.x. 20969605

[ref9] Rivas G.; Minton A. P. Macromolecular Crowding In Vitro, In Vivo, and In Between. Trends Biochem. Sci. 2016, 41 (11), 970–981. 10.1016/j.tibs.2016.08.013. 27669651 PMC5804487

[ref10] Ellis R. J. Macromolecular Crowding: Obvious but Underappreciated. Trends Biochem. Sci. 2001, 26 (10), 597–604. 10.1016/S0968-0004(01)01938-7. 11590012

[ref11] Rivas G.; Ferrone F.; Herzfeld J. Life in a Crowded World: Workshop on the Biological Implications of Macromolecular Crowding. EMBO Rep. 2004, 5 (1), 23–27. 10.1038/sj.embor.7400056. 14710181 PMC1298967

[ref12] Minton A. P. [7] Molecular Crowding: Analysis of Effects of High Concentrations of Inert Cosolutes on Biochemical Equilibria and Rates in Terms of Vol. Exclusion. In Methods in Enzymology; Elsevier, 1998; Vol. 295, pp 127–149. 10.1016/S0076-6879(98)95038-8. 9750217

[ref13] Guin D.; Gruebele M. Weak Chemical Interactions That Drive Protein Evolution: Crowding, Sticking, and Quinary Structure in Folding and Function. Chem. Rev. 2019, 119 (18), 10691–10717. 10.1021/acs.chemrev.8b00753. 31356058

[ref14] Van Den Berg J.; Boersma A. J.; Poolman B. Microorganisms Maintain Crowding Homeostasis. Nat. Rev. Microbiol. 2017, 15 (5), 309–318. 10.1038/nrmicro.2017.17. 28344349

[ref15] Zhou H. X.; Rivas G.; Minton A. P. Macromolecular Crowding and Confinement: Biochemical, Biophysical, and Potential Physiological Consequences. Annu. Rev. Biophys 2008, 37, 375–397. 10.1146/annurev.biophys.37.032807.125817. 18573087 PMC2826134

[ref16] Ellis R. J.; Minton A. P. Protein Aggregation in Crowded Environments. Biol. Chem. 2006, 387 ( (5), ), 10.1515/BC.2006.064. 16740119

[ref17] Boersma A. J.; Zuhorn I. S.; Poolman B. A Sensor for Quantification of Macromolecular Crowding in Living Cells. Nat. Methods 2015, 12 (3), 227–229. 10.1038/nmeth.3257. 25643150

[ref18] Liu B.; Poolman B.; Boersma A. J. Ionic Strength Sensing in Living Cells. ACS Chem. Biol. 2017, 12 (10), 2510–2514. 10.1021/acschembio.7b00348. 28853549 PMC5653947

[ref19] Gnutt D.; Gao M.; Brylski O.; Heyden M.; Ebbinghaus S. Excluded-Volume Effects in Living Cells. Angew. Chem., Int. Ed. 2015, 54 (8), 2548–2551. 10.1002/anie.201409847. PMC4506553 25557778

[ref20] Minton A. P. The Influence of Macromolecular Crowding and Macromolecular Confinement on Biochemical Reactions in Physiological Media. J. Biol. Chem. 2001, 276 (14), 10577–10580. 10.1074/jbc.R100005200. 11279227

[ref21] Drenckhahn D.; Pollard T. D. Elongation of Actin Filaments Is a Diffusion-Limited Reaction at the Barbed End and Is Accelerated by Inert Macromolecules. J. Biol. Chem. 1986, 261 (27), 12754–12758. 10.1016/S0021-9258(18)67157-1. 3745211

[ref22] Lindner R. A.; Ralston G. B. Macromolecular Crowding: Effects on Actin Polymerisation. Biophys. Chem. 1997, 66 (1), 57–66. 10.1016/S0301-4622(97)00011-2. 9203331

[ref23] González J. M.; Jiménez M.; Vélez M.; Mingorance J.; Andreu J. M.; Vicente M.; Rivas G. Essential Cell Division Protein FtsZ Assembles into One Monomer-Thick Ribbons under Conditions Resembling the Crowded Intracellular Environment. J. Biol. Chem. 2003, 278 (39), 37664–37671. 10.1074/jbc.M305230200. 12807907

[ref24] Ferrone F. A. Polymerization and Sickle Cell Disease: A Molecular View. Microcirculation 2004, 11 (2), 115–128. 10.1080/10739680490278312. 15280087

[ref25] Herzfeld J. Crowding-Induced Organization in Cells: Spontaneous Alignment and Sorting of Filaments with Physiological Control Points. J. Mol. Recognit. 2004, 17 (5), 376–381. 10.1002/jmr.703. 15362095

[ref26] Braun M.; Lansky Z.; Hilitski F.; Dogic Z.; Diez S. Entropic Forces Drive Contraction of Cytoskeletal Networks. BioEssays 2016, 38 (5), 474–481. 10.1002/bies.201500183. 26996935

[ref27] Ross P. D.; Briehl R. W.; Minton A. P. Temperature Dependence of Nonideality in Concentrated Solutions of Hemoglobin. Biopolymers 1978, 17 (9), 2285–2288. 10.1002/bip.1978.360170920. 698356

[ref28] Minton A. P. The Effect of Volume Occupancy upon the Thermodynamic Activity of Proteins: Some Biochemical Consequences. Mol. Cell. Biochem. 1983, 55 (2), 119–140. 10.1007/BF00673707. 6633513

[ref29] Jiao M.; Li H. T.; Chen J.; Minton A. P.; Liang Y. Attractive Protein-Polymer Interactions Markedly Alter the Effect of Macromolecular Crowding on Protein Association Equilibria. Biophys. J. 2010, 99 (3), 914–923. 10.1016/j.bpj.2010.05.013. 20682270 PMC2913179

[ref30] Fodeke A. A.; Minton A. P. Quantitative Characterization of Temperature-Independent and Temperature-Dependent Protein–Protein Interactions in Highly Nonideal Solutions. J. Phys. Chem. B 2011, 115 (38), 11261–11268. 10.1021/jp2049266. 21846103 PMC3488770

[ref31] Hoppe T.; Minton A. P. Non-Specific Interactions Between Macromolecular Solutes in Concentrated Solution: Physico-Chemical Manifestations and Biochemical Consequences. Front. Mol. Biosci. 2019, 6, 10 10.3389/fmolb.2019.00010. 30918892 PMC6424865

[ref32] Speer S. L.; Stewart C. J.; Sapir L.; Harries D.; Pielak G. J. Macromolecular Crowding Is More than Hard-Core Repulsions. Annu. Rev. Biophys. 2022, 51 (1), 267–300. 10.1146/annurev-biophys-091321-071829. 35239418

[ref33] Zimmerman S. B.; Minton A. P. Macromolecular Crowding: Biochemical, Biophysical, and Physiological Consequences. Annu. Rev. Biophys. Biomol. Struct. 1993, 22 (1), 27–65. 10.1146/annurev.bb.22.060193.000331. 7688609

[ref34] Shin Y.; Brangwynne C. P. Liquid Phase Condensation in Cell Physiology and Disease. Science 2017, 357 ( (6357), ), eaaf4382 10.1126/science.aaf4382. 28935776

[ref35] Banani S. F.; Lee H. O.; Hyman A. A.; Rosen M. K. Biomolecular Condensates: Organizers of Cellular Biochemistry. Nat. Rev. Mol. Cell Biol. 2017, 18 (5), 285–298. 10.1038/nrm.2017.7. 28225081 PMC7434221

[ref36] Alberti S.; Gladfelter A.; Mittag T. Considerations and Challenges in Studying Liquid-Liquid Phase Separation and Biomolecular Condensates. Cell 2019, 176 (3), 419–434. 10.1016/j.cell.2018.12.035. 30682370 PMC6445271

[ref37] Azaldegui C. A.; Vecchiarelli A. G.; Biteen J. S. The Emergence of Phase Separation as an Organizing Principle in Bacteria. Biophys. J. 2021, 120, 1123 10.1016/j.bpj.2020.09.023. 33186556 PMC8059088

[ref38] Pappu R. V.; Cohen S. R.; Dar F.; Farag M.; Kar M. Phase Transitions of Associative Biomacromolecules. Chem. Rev. 2023, 123 (14), 8945–8987. 10.1021/acs.chemrev.2c00814. 36881934 PMC11513790

[ref39] Ditlev J. A.; Case L. B.; Rosen M. K. Who’s In and Who’s Out—Compositional Control of Biomolecular Condensates. J. Mol. Biol. 2018, 430 (23), 4666–4684. 10.1016/j.jmb.2018.08.003. 30099028 PMC6204295

[ref40] Qian Z.-G.; Huang S.-C.; Xia X.-X. Synthetic Protein Condensates for Cellular and Metabolic Engineering. Nat. Chem. Biol. 2022, 18 (12), 1330–1340. 10.1038/s41589-022-01203-3. 36400990

[ref41] Dignon G. L.; Best R. B.; Mittal J. Biomolecular Phase Separation: From Molecular Driving Forces to Macroscopic Properties. Annu. Rev. Phys. Chem. 2020, 71 (1), 53–75. 10.1146/annurev-physchem-071819-113553. 32312191 PMC7469089

[ref42] Alberti S. Phase Separation in Biology. Curr. Biol. 2017, 27 (20), R1097–R1102. 10.1016/j.cub.2017.08.069. 29065286

[ref43] Minton A. P. Simple Calculation of Phase Diagrams for Liquid–Liquid Phase Separation in Solutions of Two Macromolecular Solute Species. J. Phys. Chem. B 2020, 124 (12), 2363–2370. 10.1021/acs.jpcb.0c00402. 32118433 PMC7104237

[ref44] André A. A. M.; Spruijt E. Liquid–Liquid Phase Separation in Crowded Environments. Int. J. Mol. Sci. 2020, 21 (16), 5908 10.3390/ijms21165908. 32824618 PMC7460619

[ref45] Martin N. Dynamic Synthetic Cells Based on Liquid–Liquid Phase Separation. ChemBioChem. 2019, 20 (20), 2553–2568. 10.1002/cbic.201900183. 31039282

[ref46] Alberti S.; Hyman A. A. Biomolecular Condensates at the Nexus of Cellular Stress, Protein Aggregation Disease and Ageing. Nat. Rev. Mol. Cell Biol. 2021, 22 (3), 196–213. 10.1038/s41580-020-00326-6. 33510441

[ref47] Musacchio A. On the Role of Phase Separation in the Biogenesis of Membraneless Compartments. EMBO J. 2022, 41 (5), e109952 10.15252/embj.2021109952. 35107832 PMC8886532

[ref48] Spruijt E. Open Questions on Liquid–Liquid Phase Separation. Commun. Chem. 2023, 6 (1), 23 10.1038/s42004-023-00823-7. 36737456 PMC9898555

[ref49] Löwe M.; Kalacheva M.; Boersma A. J.; Kedrov A. The More the Merrier: Effects of Macromolecular Crowding on the Structure and Dynamics of Biological Membranes. FEBS J. 2020, 287 (23), 5039–5067. 10.1111/febs.15429. 32463979

[ref50] Leonard T. A.; Loose M.; Martens S. The Membrane Surface as a Platform That Organizes Cellular and Biochemical Processes. Dev. Cell 2023, 58 (15), 1315–1332. 10.1016/j.devcel.2023.06.001. 37419118

[ref51] Snead W. T.; Gladfelter A. S. The Control Centers of Biomolecular Phase Separation: How Membrane Surfaces, PTMs, and Active Processes Regulate Condensation. Mol. Cell 2019, 76 (2), 295–305. 10.1016/j.molcel.2019.09.016. 31604601 PMC7173186

[ref52] Mitchison T. J. Beyond Langmuir: Surface-Bound Macromolecule Condensates. Mol. Biol. Cell 2020, 31 (23), 2502–2508. 10.1091/mbc.E20-06-0393. 33119461 PMC7851878

[ref53] Ditlev J. A. Membrane-Associated Phase Separation: Organization and Function Emerge from a Two-Dimensional Milieu. J. Mol. Cell Biol. 2021, 13 (4), 319–324. 10.1093/jmcb/mjab010. 33532844 PMC8339363

[ref54] Olivi L.; Berger M.; Creyghton R. N. P.; De Franceschi N.; Dekker C.; Mulder B. M.; Claassens N. J.; Ten Wolde P. R.; Van Der Oost J. Towards a Synthetic Cell Cycle. Nat. Commun. 2021, 12 (1), 4531 10.1038/s41467-021-24772-8. 34312383 PMC8313558

[ref55] Schwille P.; Spatz J.; Landfester K.; Bodenschatz E.; Herminghaus S.; Sourjik V.; Erb T. J.; Bastiaens P.; Lipowsky R.; Hyman A.; et al. MaxSynBio: Avenues Towards Creating Cells from the Bottom Up. Angew. Chem., Int. Ed. 2018, 57 (41), 13382–13392. 10.1002/anie.201802288. 29749673

[ref56] Poolman B. Physicochemical Homeostasis in Bacteria. FEMS Microbiol. Rev. 2023, 47 (4), fuad033 10.1093/femsre/fuad033. 37336577 PMC10368375

[ref57] Booth I. R. Bacterial Mechanosensitive Channels: Progress towards an Understanding of Their Roles in Cell Physiology. Curr. Opin. Microbiol. 2014, 18, 16–22. 10.1016/j.mib.2014.01.005. 24607989 PMC4005912

[ref58] Bremer E.; Krämer R. Responses of Microorganisms to Osmotic Stress. Annu. Rev. Microbiol. 2019, 73 (1), 313–334. 10.1146/annurev-micro-020518-115504. 31180805

[ref59] Cox C. D.; Bavi N.; Martinac B. Bacterial Mechanosensors. Annu. Rev. Physiol. 2018, 80 (1), 71–93. 10.1146/annurev-physiol-021317-121351. 29195054

[ref60] Drew D.; Boudker O. Shared Molecular Mechanisms of Membrane Transporters. Annu. Rev. Biochem. 2016, 85 (1), 543–572. 10.1146/annurev-biochem-060815-014520. 27023848

[ref61] Poolman B.; Spitzer J. J.; Wood J. M. Bacterial Osmosensing: Roles of Membrane Structure and Electrostatics in Lipid–Protein and Protein–Protein Interactions.. Biochim. Biophys. Acta BBA - Biomembr. 2004, 1666 (1–2), 88–104. 10.1016/j.bbamem.2004.06.013. 15519310

[ref62] Wood J. M. Bacterial Osmoregulation: A Paradigm for the Study of Cellular Homeostasis. Annu. Rev. Microbiol. 2011, 65 (1), 215–238. 10.1146/annurev-micro-090110-102815. 21663439

[ref63] Milo R.; Phillips R. Cell Biology by the Numbers, 0 ed.; Garland Science, 2015. 10.1201/9780429258770.

[ref64] Cayley S.; Record M. T. Large Changes in Cytoplasmic Biopolymer Concentration with Osmolality Indicate That Macromolecular Crowding May Regulate Protein–DNA Interactions and Growth Rate in Osmotically stressedEscherichia Coli K-12. J. Mol. Recognit. 2004, 17 (5), 488–496. 10.1002/jmr.695. 15362109

[ref65] Konopka M. C.; Sochacki K. A.; Bratton B. P.; Shkel I. A.; Record M. T.; Weisshaar J. C. Cytoplasmic Protein Mobility in Osmotically Stressed *Escherichia Coli*. J. Bacteriol. 2009, 191 (1), 231–237. 10.1128/JB.00536-08. 18952804 PMC2612437

[ref66] Liu B.; Hasrat Z.; Poolman B.; Boersma A. J. Decreased Effective Macromolecular Crowding in Escherichia Coli Adapted to Hyperosmotic Stress. J. Bacteriol. 2019, 201 ( (10), ), 10.1128/JB.00708-18 PMC6482933 30833357

[ref67] Mika J. T.; Van Den Bogaart G.; Veenhoff L.; Krasnikov V.; Poolman B. Molecular Sieving Properties of the Cytoplasm of Escherichia Coli and Consequences of Osmotic Stress: Molecule Diffusion and Barriers in the Cytoplasm. Mol. Microbiol. 2010, 77 (1), 200–207. 10.1111/j.1365-2958.2010.07201.x. 20487282

[ref68] Schavemaker P. E.; Boersma A. J.; Poolman B. How Important Is Protein Diffusion in Prokaryotes? Front. Mol. Biosci. 2018, 5, 10.3389/fmolb.2018.00093. PMC6243074 30483513

[ref69] Pang T. Y.; Lercher M. J. Optimal Density of Bacterial Cells. PLOS Comput. Biol. 2023, 19 (6), e1011177 10.1371/journal.pcbi.1011177. 37307285 PMC10289677

[ref70] Sikkema H. R.; Gaastra B. F.; Pols T.; Poolman B. Cell Fuelling and Metabolic Energy Conservation in Synthetic Cells. ChemBioChem. 2019, 20 (20), 2581–2592. 10.1002/cbic.201900398. 31381223

[ref71] Krulwich T. A.; Sachs G.; Padan E. Molecular Aspects of Bacterial pH Sensing and Homeostasis. Nat. Rev. Microbiol. 2011, 9 (5), 330–343. 10.1038/nrmicro2549. 21464825 PMC3247762

[ref72] Levering J.; Musters M. W. J. M.; Bekker M.; Bellomo D.; Fiedler T.; De Vos W. M.; Hugenholtz J.; Kreikemeyer B.; Kummer U.; Teusink B. Role of Phosphate in the Central Metabolism of Two Lactic Acid Bacteria - a Comparative Systems Biology Approach: Modelling Glycolysis of Lactic Acid Bacteria. FEBS J. 2012, 279 (7), 1274–1290. 10.1111/j.1742-4658.2012.08523.x. 22325620

[ref73] Booth I. R. Regulation of Cytoplasmic pH in Bacteria. Microbiol. Rev. 1985, 49 (4), 359–378. 10.1128/mr.49.4.359-378.1985. 3912654 PMC373043

[ref74] Krulwich T. A.; Agus R.; Schneier M.; Guffanti A. A. Buffering Capacity of Bacilli That Grow at Different pH Ranges. J. Bacteriol. 1985, 162 (2), 768–772. 10.1128/jb.162.2.768-772.1985. 3886633 PMC218917

[ref75] Alexeeva S.; Hellingwerf K. J.; Teixeira De Mattos M. J. Quantitative Assessment of Oxygen Availability: Perceived Aerobiosis and Its Effect on Flux Distribution in the Respiratory Chain of *Escherichia Coli*. J. Bacteriol. 2002, 184 (5), 1402–1406. 10.1128/JB.184.5.1402-1406.2002. 11844770 PMC134846

[ref76] Healy J.; Ekkerman S.; Pliotas C.; Richard M.; Bartlett W.; Grayer S. C.; Morris G. M.; Miller S.; Booth I. R.; Conway S. J.; Rasmussen T. Understanding the Structural Requirements for Activators of the Kef Bacterial Potassium Efflux System. Biochemistry 2014, 53 (12), 1982–1992. 10.1021/bi5001118. 24601535 PMC4004266

[ref77] Pliotas C.; Grayer S. C.; Ekkerman S.; Chan A. K. N.; Healy J.; Marius P.; Bartlett W.; Khan A.; Cortopassi W. A.; Chandler S. A.; et al. Adenosine Monophosphate Binding Stabilizes the KTN Domain of the *Shewanella Denitrificans* Kef Potassium Efflux System. Biochemistry 2017, 56 (32), 4219–4234. 10.1021/acs.biochem.7b00300. 28656748 PMC5645763

[ref78] Taglicht D.; Padan E.; Schuldiner S. Overproduction and Purification of a Functional Na+/H+ Antiporter Coded by nhaA (Ant) from Escherichia Coli. J. Biol. Chem. 1991, 266 (17), 11289–11294. 10.1016/S0021-9258(18)99161-1. 1645730

[ref79] Winkelmann I.; Uzdavinys P.; Kenney I. M.; Brock J.; Meier P. F.; Wagner L.-M.; Gabriel F.; Jung S.; Matsuoka R.; Von Ballmoos C.; et al. Crystal Structure of the Na+/H+ Antiporter NhaA at Active pH Reveals the Mechanistic Basis for pH Sensing. Nat. Commun. 2022, 13 (1), 6383 10.1038/s41467-022-34120-z. 36289233 PMC9606361

[ref80] Chavan T. S.; Cheng R. C.; Jiang T.; Mathews I. I.; Stein R. A.; Koehl A.; Mchaourab H. S.; Tajkhorshid E.; Maduke M. A CLC-Ec1Mutant Reveals Global Conformational Change and Suggests a Unifying Mechanism for the CLC Cl–/H+ Transport Cycle. eLife 2020, 9, e53479 10.7554/eLife.53479. 32310757 PMC7253180

[ref81] Anantharam V.; Allison M. J.; Maloney P. C. Oxalate:Formate Exchange. J. Biol. Chem. 1989, 264 (13), 7244–7250. 10.1016/S0021-9258(18)83227-6. 2708365

[ref82] Poolman B.; Molenaar D.; Smid E. J.; Ubbink T.; Abee T.; Renault P. P.; Konings W. N. Malolactic Fermentation: Electrogenic Malate Uptake and Malate/Lactate Antiport Generate Metabolic Energy. J. Bacteriol. 1991, 173 (19), 6030–6037. 10.1128/jb.173.19.6030-6037.1991. 1917837 PMC208348

[ref83] Romano A.; Trip H.; Lolkema J. S.; Lucas P. M. Three-Component Lysine/Ornithine Decarboxylation System in Lactobacillus Saerimneri 30a. J. Bacteriol. 2013, 195 (6), 1249–1254. 10.1128/JB.02070-12. 23316036 PMC3592000

[ref84] Salema M.; Poolman B.; Lolkema J. S.; Dias M. C. L.; Konings W. N. Uniport of Monoanionic L-Malate in Membrane Vesicles from Leuconostoc Oenos. Eur. J. Biochem. 1994, 225 (1), 289–295. 10.1111/j.1432-1033.1994.00289.x. 7925448

[ref85] Sa H. D.; Park J. Y.; Jeong S.-J.; Lee K. W.; Kim J. H. Characterization of Glutamate Decarboxylase (GAD) from Lactobacillus Sakei A156 Isolated from Jeot-Gal. J. Microbiol. Biotechnol. 2015, 25 (5), 696–703. 10.4014/jmb.1412.12075. 25791853

[ref86] Gale E. F. Estimation of l(+)-Arginine in Protein Hydrolysates by the Use of l(+)-Arginine Decarboxylase. Nature 1946, 157 (3983), 265–265. 10.1038/157265a0. 21016868

[ref87] Suzuki T.; Kobayashi H. Regulation of the Cytoplasmic pH by a Proton-Translocating ATPase in Streptococcus Faecalis (Faecium). A Computer Simulation. Eur. J. Biochem. 1989, 180 (2), 467–471. 10.1111/j.1432-1033.1989.tb14669.x. 2522391

[ref88] Schavemaker P. E.; Śmigiel W. M.; Poolman B. Ribosome Surface Properties May Impose Limits on the Nature of the Cytoplasmic Proteome. eLife 2017, 6, e30084 10.7554/eLife.30084. 29154755 PMC5726854

[ref89] König I.; Zarrine-Afsar A.; Aznauryan M.; Soranno A.; Wunderlich B.; Dingfelder F.; Stüber J. C.; Plückthun A.; Nettels D.; Schuler B. Single-Molecule Spectroscopy of Protein Conformational Dynamics in Live Eukaryotic Cells. Nat. Methods 2015, 12 (8), 773–779. 10.1038/nmeth.3475. 26147918

[ref90] Nørby J. G.; Esmann M. The Effect of Ionic Strength and Specific Anions on Substrate Binding and Hydrolytic Activities of Na,K-ATPase. J. Gen. Physiol. 1997, 109 (5), 555–570. 10.1085/jgp.109.5.555. 9154904 PMC2217059

[ref91] Syeda R.; Qiu Z.; Dubin A. E.; Murthy S. E.; Florendo M. N.; Mason D. E.; Mathur J.; Cahalan S. M.; Peters E. C.; Montal M.; Patapoutian A. LRRC8 Proteins Form Volume-Regulated Anion Channels That Sense Ionic Strength. Cell 2016, 164 (3), 499–511. 10.1016/j.cell.2015.12.031. 26824658 PMC4733249

[ref92] Biemans-Oldehinkel E.; Mahmood N. A. B. N.; Poolman B. A Sensor for Intracellular Ionic Strength. Proc. Natl. Acad. Sci. U. S. A. 2006, 103 (28), 10624–10629. 10.1073/pnas.0603871103. 16815971 PMC1502282

[ref93] Marek P. J.; Patsalo V.; Green D. F.; Raleigh D. P. Ionic Strength Effects on Amyloid Formation by Amylin Are a Complicated Interplay among Debye Screening, Ion Selectivity, and Hofmeister Effects. Biochemistry 2012, 51 (43), 8478–8490. 10.1021/bi300574r. 23016872 PMC3753197

[ref94] Elbaum-Garfinkle S.; Kim Y.; Szczepaniak K.; Chen C. C.-H.; Eckmann C. R.; Myong S.; Brangwynne C. P. The Disordered P Granule Protein LAF-1 Drives Phase Separation into Droplets with Tunable Viscosity and Dynamics. Proc. Natl. Acad. Sci. U. S. A. 2015, 112 (23), 7189–7194. 10.1073/pnas.1504822112. 26015579 PMC4466716

[ref95] Record M. T.; Anderson C. F.; Lohman T. M. Thermodynamic Analysis of Ion Effects on the Binding and Conformational Equilibria of Proteins and Nucleic Acids: The Roles of Ion Association or Release, Screening, and Ion Effects on Water Activity. Q. Rev. Biophys. 1978, 11 (2), 103–178. 10.1017/S003358350000202X. 353875

[ref96] Booth I. R.; Higgins C. F. Enteric Bacteria and Osmotic Stress: Intracellular Potassium Glutamate as a Secondary Signal of Osmotic Stress?. FEMS Microbiol. Lett. 1990, 75 (2–3), 239–246. 10.1111/j.1574-6968.1990.tb04097.x. 1974769

[ref97] Dinnbier U.; Limpinsel E.; Schmid R.; Bakker E. P. Transient Accumulation of Potassium Glutamate and Its Replacement by Trehalose during Adaptation of Growing Cells of Escherichia Coli K-12 to Elevated Sodium Chloride Concentrations. Arch. Microbiol. 1988, 150 (4), 348–357. 10.1007/BF00408306. 3060036

[ref98] Buda R.; Liu Y.; Yang J.; Hegde S.; Stevenson K.; Bai F.; Pilizota T. Dynamics of *Escherichia Coli* ’s Passive Response to a Sudden Decrease in External Osmolarity. Proc. Natl. Acad. Sci. U. S. A. 2016, 113 ( (40), ), E5838–E5846 10.1073/pnas.1522185113. 27647888 PMC5056102

[ref99] Culham D. E.; Shkel I. A.; Record M. T.; Wood J. M. Contributions of Coulombic and Hofmeister Effects to the Osmotic Activation of *Escherichia Coli* Transporter ProP. Biochemistry 2016, 55 (9), 1301–1313. 10.1021/acs.biochem.5b01169. 26871755 PMC4850843

[ref100] Ziegler C.; Bremer E.; Krämer R. The BCCT Family of Carriers: From Physiology to Crystal Structure: BCCT Carriers. Mol. Microbiol. 2010, 78 (1), 13–34. 10.1111/j.1365-2958.2010.07332.x. 20923416

[ref101] Spitzer J. J.; Poolman B. Electrochemical Structure of the Crowded Cytoplasm. Trends Biochem. Sci. 2005, 30 (10), 536–541. 10.1016/j.tibs.2005.08.002. 16125938

[ref102] Sundararaman R.; Vigil-Fowler D.; Schwarz K. Improving the Accuracy of Atomistic Simulations of the Electrochemical Interface. Chem. Rev. 2022, 122 (12), 10651–10674. 10.1021/acs.chemrev.1c00800. 35522135 PMC10127457

[ref103] Sikkema H. R.; Van Den Noort M.; Rheinberger J.; De Boer M.; Krepel S. T.; Schuurman-Wolters G. K.; Paulino C.; Poolman B. Gating by Ionic Strength and Safety Check by Cyclic-Di-AMP in the ABC Transporter OpuA. Sci. Adv. 2020, 6 (47), eabd7697 10.1126/sciadv.abd7697. 33208376 PMC7673798

[ref104] García-Heredia A. Plasma Membrane-Cell Wall Feedback in Bacteria. J. Bacteriol. 2023, 205 (3), e00433–22. 10.1128/jb.00433-22. 36794934 PMC10029715

[ref105] Tran B. M.; Prabha H.; Iyer A.; O’Byrne C.; Abee T.; Poolman B. Measurement of Protein Mobility in Listeria Monocytogenes Reveals a Unique Tolerance to Osmotic Stress and Temperature Dependence of Diffusion. Front. Microbiol. 2021, 12, 640149 10.3389/fmicb.2021.640149. 33679676 PMC7925416

[ref106] Whatmore A. M.; Reed R. H. Determination of Turgor Pressure in Bacillus Subtilis: A Possible Role for K+ in Turgor Regulation. J. Gen. Microbiol. 1990, 136 (12), 2521–2526. 10.1099/00221287-136-12-2521. 2127801

[ref107] Oldewurtel E. R.; Kitahara Y.; van Teeffelen S. Robust Surface-to-Mass Coupling and Turgor-Dependent Cell Width Determine Bacterial Dry-Mass Density. Proc. Natl. Acad. Sci. U. S. A. 2021, 118 (32), e2021416118 10.1073/pnas.2021416118. 34341116 PMC8364103

[ref108] Scott Cayley D.; Guttman H. J.; Thomas Record M. Biophysical Characterization of Changes in Amounts and Activity of Escherichia Coli Cell and Compartment Water and Turgor Pressure in Response to Osmotic Stress. Biophys. J. 2000, 78 (4), 1748–1764. 10.1016/S0006-3495(00)76726-9. 10733957 PMC1300771

[ref109] Koch A. L. Shrinkage of Growing Escherichia Coli Cells by Osmotic Challenge. J. Bacteriol. 1984, 159 (3), 919–924. 10.1128/jb.159.3.919-924.1984. 6384186 PMC215747

[ref110] Pasquina-Lemonche L.; Burns J.; Turner R. D.; Kumar S.; Tank R.; Mullin N.; Wilson J. S.; Chakrabarti B.; Bullough P. A.; Foster S. J.; Hobbs J. K. The Architecture of the Gram-Positive Bacterial Cell Wall. Nature 2020, 582 (7811), 294–297. 10.1038/s41586-020-2236-6. 32523118 PMC7308169

[ref111] Wood J. M.; Bremer E.; Csonka L. N.; Kraemer R.; Poolman B.; Van Der Heide T.; Smith L. T. Osmosensing and Osmoregulatory Compatible Solute Accumulation by Bacteria. Comp. Biochem. Physiol. A. Mol. Integr. Physiol. 2001, 130 (3), 437–460. 10.1016/S1095-6433(01)00442-1. 11913457

[ref112] Yancey P. H.; Clark M. E.; Hand S. C.; Bowlus R. D.; Somero G. N. Living with Water Stress: Evolution of Osmolyte Systems. Science 1982, 217 (4566), 1214–1222. 10.1126/science.7112124. 7112124

[ref113] Wargo M. J.; Meadows J. A. Carnitine in Bacterial Physiology and Metabolism. Microbiology 2015, 161 (6), 1161–1174. 10.1099/mic.0.000080. 25787873 PMC4635513

[ref114] Goodsell D. S. Escherichia Coli Bacterium. RCSB Protein Data Bank 2021. 10.2210/rcsb_pdb/goodsell-gallery-028.

[ref115] Van Der Heide T. On the Osmotic Signal and Osmosensing Mechanism of an ABC Transport System for Glycine Betaine. EMBO J. 2001, 20 (24), 7022–7032. 10.1093/emboj/20.24.7022. 11742979 PMC125795

[ref116] Karasawa A.; Swier L. J. Y. M.; Stuart M. C. A.; Brouwers J.; Helms B.; Poolman B. Physicochemical Factors Controlling the Activity and Energy Coupling of an Ionic Strength-Gated ATP-Binding Cassette (ABC) Transporter. J. Biol. Chem. 2013, 288 (41), 29862–29871. 10.1074/jbc.M113.499327. 23979139 PMC3795284

[ref117] Fuss M. F.; Wieferig J.-P.; Corey R. A.; Hellmich Y.; Tascón I.; Sousa J. S.; Stansfeld P. J.; Vonck J.; Hänelt I. Cyclic Di-AMP Traps Proton-Coupled K+ Transporters of the KUP Family in an Inward-Occluded Conformation. Nat. Commun. 2023, 14 (1), 3683 10.1038/s41467-023-38944-1. 37344476 PMC10284832

[ref118] Gibhardt J.; Hoffmann G.; Turdiev A.; Wang M.; Lee V. T.; Commichau F. M. C-Di-AMP Assists Osmoadaptation by Regulating the Listeria Monocytogenes Potassium Transporters KimA and KtrCD. J. Biol. Chem. 2019, 294 (44), 16020–16033. 10.1074/jbc.RA119.010046. 31506295 PMC6827311

[ref119] Gundlach J.; Krüger L.; Herzberg C.; Turdiev A.; Poehlein A.; Tascón I.; Weiss M.; Hertel D.; Daniel R.; Hänelt I.; Lee V. T.; Stülke J. Sustained Sensing in Potassium Homeostasis: Cyclic Di-AMP Controls Potassium Uptake by KimA at the Levels of Expression and Activity. J. Biol. Chem. 2019, 294 (24), 9605–9614. 10.1074/jbc.RA119.008774. 31061098 PMC6579464

[ref120] Stülke J.; Krüger L. Cyclic Di-AMP Signaling in Bacteria. Annu. Rev. Microbiol. 2020, 74 (1), 159–179. 10.1146/annurev-micro-020518-115943. 32603625

[ref121] Handbook of Electroporation; Miklavčič D., Ed.; Springer International Publishing: Cham, 2017. 10.1007/978-3-319-32886-7.

[ref122] Edwards M. D.; Black S.; Rasmussen T.; Rasmussen A.; Stokes N. R.; Stephen T.-L.; Miller S.; Booth I. R. Characterization of Three Novel Mechanosensitive Channel Activities in *Escherichia Coli*. Channels 2012, 6 (4), 272–281. 10.4161/chan.20998. 22874652 PMC3508906

[ref123] Sukharev S. I.; Sigurdson W. J.; Kung C.; Sachs F. Energetic and Spatial Parameters for Gating of the Bacterial Large Conductance Mechanosensitive Channel. MscL. J. Gen. Physiol. 1999, 113 (4), 525–540. 10.1085/jgp.113.4.525. 10102934 PMC2217166

[ref124] Edwards M. D.; Li Y.; Kim S.; Miller S.; Bartlett W.; Black S.; Dennison S.; Iscla I.; Blount P.; Bowie J. U.; Booth I. R. Pivotal Role of the Glycine-Rich TM3 Helix in Gating the MscS Mechanosensitive Channel. Nat. Struct. Mol. Biol. 2005, 12 (2), 113–119. 10.1038/nsmb895. 15665866

[ref125] Rasmussen T.; Rasmussen A.; Singh S.; Galbiati H.; Edwards M. D.; Miller S.; Booth I. R. Properties of the Mechanosensitive Channel MscS Pore Revealed by Tryptophan Scanning Mutagenesis. Biochemistry 2015, 54 (29), 4519–4530. 10.1021/acs.biochem.5b00294. 26126964 PMC4519979

[ref126] Boer M.; Anishkin A.; Sukharev S. Adaptive MscS Gating in the Osmotic Permeability Response in *E. Coli*: The Question of Time. Biochemistry 2011, 50 (19), 4087–4096. 10.1021/bi1019435. 21456519 PMC3170927

[ref127] Moe P.; Blount P. Assessment of Potential Stimuli for Mechano-Dependent Gating of MscL: Effects of Pressure, Tension, and Lipid Headgroups. Biochemistry 2005, 44 (36), 12239–12244. 10.1021/bi0509649. 16142922

[ref128] O’Connell J. D.; Zhao A.; Ellington A. D.; Marcotte E. M. Dynamic Reorganization of Metabolic Enzymes into Intracellular Bodies. Annu. Rev. Cell Dev. Biol. 2012, 28 (1), 89–111. 10.1146/annurev-cellbio-101011-155841. 23057741 PMC4089986

[ref129] Petrovska I.; Nüske E.; Munder M. C.; Kulasegaran G.; Malinovska L.; Kroschwald S.; Richter D.; Fahmy K.; Gibson K.; Verbavatz J.-M.; Alberti S. Filament Formation by Metabolic Enzymes Is a Specific Adaptation to an Advanced State of Cellular Starvation. eLife 2014, 3, e02409 10.7554/eLife.02409. 24771766 PMC4011332

[ref130] Grant C. R.; Wan J.; Komeili A. Organelle Formation in Bacteria and Archaea. Annu. Rev. Cell Dev. Biol. 2018, 34 (1), 217–238. 10.1146/annurev-cellbio-100616-060908. 30113887

[ref131] Giessen T. W. Encapsulins. Annu. Rev. Biochem. 2022, 91 (1), 353–380. 10.1146/annurev-biochem-040320-102858. PMC9944552 35303791

[ref132] Greening C.; Lithgow T. Formation and Function of Bacterial Organelles. Nat. Rev. Microbiol. 2020, 18 (12), 677–689. 10.1038/s41579-020-0413-0. 32710089

[ref133] Chai Q.; Singh B.; Peisker K.; Metzendorf N.; Ge X.; Dasgupta S.; Sanyal S. Organization of Ribosomes and Nucleoids in Escherichia Coli Cells during Growth and in Quiescence. J. Biol. Chem. 2014, 289 (16), 11342–11352. 10.1074/jbc.M114.557348. 24599955 PMC4036271

[ref134] Wu F.; Swain P.; Kuijpers L.; Zheng X.; Felter K.; Guurink M.; Solari J.; Jun S.; Shimizu T. S.; Chaudhuri D.; Mulder B.; Dekker C. Cell Boundary Confinement Sets the Size and Position of the E. Coli Chromosome. Curr. Biol. 2019, 29 (13), 2131–2144.e4. 10.1016/j.cub.2019.05.015. 31155353 PMC7050463

[ref135] Zimmerman S. B.; Murphy L. D. Macromolecular Crowding and the Mandatory Condensation of DNA in Bacteria. FEBS Lett. 1996, 390 (3), 245–248. 10.1016/0014-5793(96)00725-9. 8706869

[ref136] Bakshi S.; Choi H.; Weisshaar J. C. The Spatial Biology of Transcription and Translation in Rapidly Growing Escherichia Coli. Front. Microbiol. 2015, 6, 10.3389/fmicb.2015.00636. PMC4488752 26191045

[ref137] Bakshi S.; Siryaporn A.; Goulian M.; Weisshaar J. C. Superresolution Imaging of Ribosomes and RNA Polymerase in Live Escherichia Coli Cells. Mol. Microbiol. 2012, 85 (1), 21–38. 10.1111/j.1365-2958.2012.08081.x. 22624875 PMC3383343

[ref138] Winkler J.; Seybert A.; König L.; Pruggnaller S.; Haselmann U.; Sourjik V.; Weiss M.; Frangakis A. S.; Mogk A.; Bukau B. Quantitative and Spatio-Temporal Features of Protein Aggregation in Escherichia Coli and Consequences on Protein Quality Control and Cellular Ageing. EMBO J. 2010, 29 (5), 910–923. 10.1038/emboj.2009.412. 20094032 PMC2837176

[ref139] Coquel A.-S.; Jacob J.-P.; Primet M.; Demarez A.; Dimiccoli M.; Julou T.; Moisan L.; Lindner A. B.; Berry H. Localization of Protein Aggregation in Escherichia Coli Is Governed by Diffusion and Nucleoid Macromolecular Crowding Effect. PLOS Comput. Biol. 2013, 9 (4), e1003038 10.1371/journal.pcbi.1003038. 23633942 PMC3636022

[ref140] Schramm F. D.; Schroeder K.; Jonas K. Protein Aggregation in Bacteria. FEMS Microbiol. Rev. 2020, 44 (1), 54–72. 10.1093/femsre/fuz026. 31633151 PMC7053576

[ref141] Śmigiel W. M.; Mantovanelli L.; Linnik D. S.; Punter M.; Silberberg J.; Xiang L.; Xu K.; Poolman B. Protein Diffusion in *Escherichia Coli* Cytoplasm Scales with the Mass of the Complexes and Is Location Dependent. Sci. Adv. 2022, 8 (32), eabo5387 10.1126/sciadv.abo5387. 35960807 PMC9374337

[ref142] Xiang L.; Chen K.; Yan R.; Li W.; Xu K. Single-Molecule Displacement Mapping Unveils Nanoscale Heterogeneities in Intracellular Diffusivity. Nat. Methods 2020, 17 (5), 524–530. 10.1038/s41592-020-0793-0. 32203387 PMC7205592

[ref143] Mantovanelli L.; Linnik D. S.; Punter M.; Kojakhmetov H. J.; Śmigiel W. M.; Poolman B. Simulation-Based Reconstructed Diffusion Unveils the Effect of Aging on Protein Diffusion in Escherichia Coli. PLOS Comput. Biol. 2023, 19 (9), e1011093 10.1371/journal.pcbi.1011093. 37695774 PMC10513214

[ref144] Dendooven T.; Paris G.; Shkumatov A. V.; Islam Md. S.; Burt A.; Kubańska M. A.; Yang T. Y.; Hardwick S. W.; Luisi B. F. Multi-scale Ensemble Properties of the Escherichia Coli RNA Degradosome. Mol. Microbiol. 2022, 117 (1), 102–120. 10.1111/mmi.14800. 34415624 PMC7613265

[ref145] Ladouceur A. M.; Parmar B. S.; Biedzinski S.; Wall J.; Tope S. G.; Cohn D.; Kim A.; Soubry N.; Reyes-Lamothe R.; Weber S. C. Clusters of Bacterial RNA Polymerase Are Biomolecular Condensates That Assemble through Liquid-Liquid Phase Separation. Proc. Natl. Acad. Sci. U A 2020, 117 (31), 18540–18549. 10.1073/pnas.2005019117. PMC7414142 32675239

[ref146] Lasker K.; Boeynaems S.; Lam V.; Scholl D.; Stainton E.; Briner A.; Jacquemyn M.; Daelemans D.; Deniz A.; Villa E.; Holehouse A. S.; Gitler A. D.; Shapiro L. The Material Properties of a Bacterial-Derived Biomolecular Condensate Tune Biological Function in Natural and Synthetic Systems. Nat. Commun. 2022, 13 (1), 5643 10.1038/s41467-022-33221-z. 36163138 PMC9512792

[ref147] Babl L.; Merino-Salomón A.; Kanwa N.; Schwille P. Membrane Mediated Phase Separation of the Bacterial Nucleoid Occlusion Protein Noc. Sci. Rep. 2022, 12 (1), 17949 10.1038/s41598-022-22680-5. 36289351 PMC9606368

[ref148] Guilhas B.; Walter J.-C.; Rech J.; David G.; Walliser N. O.; Palmeri J.; Mathieu-Demaziere C.; Parmeggiani A.; Bouet J.-Y.; Le Gall A.; Nollmann M. ATP-Driven Separation of Liquid Phase Condensates in Bacteria. Mol. Cell 2020, 79 (2), 293–303.e4. 10.1016/j.molcel.2020.06.034. 32679076

[ref149] Jin X.; Lee J.-E.; Schaefer C.; Luo X.; Wollman A. J. M.; Payne-Dwyer A. L.; Tian T.; Zhang X.; Chen X.; Li Y.; McLeish T. C. B.; Leake M. C.; Bai F. Membraneless Organelles Formed by Liquid-Liquid Phase Separation Increase Bacterial Fitness. Sci. Adv. 2021, 7 (43), eabh2929 10.1126/sciadv.abh2929. 34669478 PMC8528417

[ref150] Saurabh S.; Chong T. N.; Bayas C.; Dahlberg P. D.; Cartwright H. N.; Moerner W. E.; Shapiro L. ATP-Responsive Biomolecular Condensates Tune Bacterial Kinase Signaling. Sci. Adv. 2022, 8 (7), eabm6570 10.1126/sciadv.abm6570. 35171683 PMC8849385

[ref151] Collins M. J.; Tomares D. T.; Nandana V.; Schrader J. M.; Childers W. S. RNase E Biomolecular Condensates Stimulate PNPase Activity. Sci. Rep. 2023, 13 (1), 12937 10.1038/s41598-023-39565-w. 37558691 PMC10412687

[ref152] Norris V.; Blaauwen T. D.; Doi R. H.; Harshey R. M.; Janniere L.; Jiménez-Sánchez A.; Jin D. J.; Levin P. A.; Mileykovskaya E.; Minsky A.; et al. Toward a Hyperstructure Taxonomy. Annu. Rev. Microbiol. 2007, 61 (1), 309–329. 10.1146/annurev.micro.61.081606.103348. 17896876

[ref153] Srere P. A. COMPLEXES OF SEQUENTIAL METABOLIC ENZYMES. Annu. Rev. Biochem. 1987, 56 (1), 89–124. 10.1146/annurev.bi.56.070187.000513. 2441660

[ref154] Hoang Y.; Azaldegui C. A.; Ghalmi M.; Biteen J. S.; Vecchiarelli A. G. An Experimental Framework to Assess Biomolecular Condensates in Bacteria. bioRxiv 2023, 10.1101/2023.03.22.533878. PMC11018776 38622124

[ref155] Parry B. R.; Surovtsev I. V.; Cabeen M. T.; O’Hern C. S.; Dufresne E. R.; Jacobs-Wagner C. The Bacterial Cytoplasm Has Glass-like Properties and Is Fluidized by Metabolic Activity. Cell 2014, 156 (1–2), 183–194. 10.1016/j.cell.2013.11.028. 24361104 PMC3956598

[ref156] Konopka M. C.; Shkel I. A.; Cayley S.; Record M. T.; Weisshaar J. C. Crowding and Confinement Effects on Protein Diffusion In Vivo. J. Bacteriol. 2006, 188 (17), 6115–6123. 10.1128/JB.01982-05. 16923878 PMC1595386

[ref157] Van Den Bogaart G.; Hermans N.; Krasnikov V.; Poolman B. Protein Mobility and Diffusive Barriers in *Escherichia Coli*: Consequences of Osmotic Stress: Protein Diffusion in *E*. Coli. Mol. Microbiol. 2007, 64 (3), 858–871. 10.1111/j.1365-2958.2007.05705.x. 17462029

[ref158] Joyner R. P.; Tang J. H.; Helenius J.; Dultz E.; Brune C.; Holt L. J.; Huet S.; Müller D. J.; Weis K. A Glucose-Starvation Response Regulates the Diffusion of Macromolecules. eLife 2016, 5, e09376 10.7554/eLife.09376. 27003290 PMC4811765

[ref159] Xie Y.; Gresham D.; Holt L. J. Increased Mesoscale Diffusivity in Response to Acute Glucose Starvation. MicroPublication Biol. 2023, 2023, 10.17912/micropub.biology.000729. PMC9996311 36908311

[ref160] Munder M. C.; Midtvedt D.; Franzmann T.; Nuske E.; Otto O.; Herbig M.; Ulbricht E.; Muller P.; Taubenberger A.; Maharana S.; Malinovska L.; Richter D.; Guck J.; Zaburdaev V.; Alberti S. A pH-Driven Transition of the Cytoplasm from a Fluid- to a Solid-like State Promotes Entry into Dormancy. Elife 2016, 5, 10.7554/eLife.09347. PMC4850707 27003292

[ref161] Åberg C.; Poolman B. Glass-like Characteristics of Intracellular Motion in Human Cells. Biophys. J. 2021, 120 (11), 2355–2366. 10.1016/j.bpj.2021.04.011. 33887228 PMC8390805

[ref162] Delarue M.; Brittingham G. P.; Pfeffer S.; Surovtsev I. V.; Pinglay S.; Kennedy K. J.; Schaffer M.; Gutierrez J. I.; Sang D.; Poterewicz G.; et al. J. mTORC1 Controls Phase Separation and the Biophysical Properties of the Cytoplasm by Tuning Crowding. Cell 2018, 174 (2), 338–349.e20. 10.1016/j.cell.2018.05.042. 29937223 PMC10080728

[ref163] Losa J.; Leupold S.; Alonso-Martinez D.; Vainikka P.; Thallmair S.; Tych K. M.; Marrink S. J.; Heinemann M. Perspective: A Stirring Role for Metabolism in Cells. Mol. Syst. Biol. 2022, 18 (4), e10822 10.15252/msb.202110822. 35362256 PMC8972047

[ref164] Persson L. B.; Ambati V. S.; Brandman O. Cellular Control of Viscosity Counters Changes in Temperature and Energy Availability. Cell 2020, 183 (6), 1572–1585.e16. 10.1016/j.cell.2020.10.017. 33157040 PMC7736452

[ref165] Leuenberger P.; Ganscha S.; Kahraman A.; Cappelletti V.; Boersema P. J.; Von Mering C.; Claassen M.; Picotti P. Cell-Wide Analysis of Protein Thermal Unfolding Reveals Determinants of Thermostability. Science 2017, 355 (6327), eaai7825 10.1126/science.aai7825. 28232526

[ref166] Di Bari D.; Timr S.; Guiral M.; Giudici-Orticoni M.-T.; Seydel T.; Beck C.; Petrillo C.; Derreumaux P.; Melchionna S.; Sterpone F.; Peters J.; Paciaroni A. Diffusive Dynamics of Bacterial Proteome as a Proxy of Cell Death. ACS Cent. Sci. 2023, 9 (1), 93–102. 10.1021/acscentsci.2c01078. 36712493 PMC9881203

[ref167] Einstein A. Uber Die von Der, 1905.

[ref168] Bellotto N.; Agudo-Canalejo J.; Colin R.; Golestanian R.; Malengo G.; Sourjik V. Dependence of Diffusion in Escherichia Coli Cytoplasm on Protein Size, Environmental Conditions, and Cell Growth. eLife 2022, 11, e82654. 10.7554/eLife.82654 36468683 PMC9810338

[ref169] Elowitz M. B.; Surette M. G.; Wolf P.-E.; Stock J. B.; Leibler S. Protein Mobility in the Cytoplasm of *Escherichia Coli*. J. Bacteriol. 1999, 181 (1), 197–203. 10.1128/JB.181.1.197-203.1999. 9864330 PMC103549

[ref170] Kumar M.; Mommer M. S.; Sourjik V. Mobility of Cytoplasmic, Membrane, and DNA-Binding Proteins in Escherichia Coli. Biophys. J. 2010, 98 (4), 552–559. 10.1016/j.bpj.2009.11.002. 20159151 PMC2820653

[ref171] Mullineaux C. W.; Nenninger A.; Ray N.; Robinson C. Diffusion of Green Fluorescent Protein in Three Cell Environments in *Escherichia Coli*. J. Bacteriol. 2006, 188 (10), 3442–3448. 10.1128/JB.188.10.3442-3448.2006. 16672597 PMC1482841

[ref172] Mika J. T.; Poolman B. Macromolecule Diffusion and Confinement in Prokaryotic Cells. Curr. Opin. Biotechnol. 2011, 22 (1), 117–126. 10.1016/j.copbio.2010.09.009. 20952181

[ref173] Xiang Y.; Surovtsev I. V.; Chang Y.; Govers S. K.; Parry B. R.; Liu J.; Jacobs-Wagner C. Interconnecting Solvent Quality, Transcription, and Chromosome Folding in Escherichia coli. Cell 2021, 184 (14), 3626–3642.e14. 10.1016/j.cell.2021.05.037. 34186018

[ref174] Tran B. M.; Linnik D. S.; Punter C. M.; Śmigiel W. M.; Mantovanelli L.; Iyer A.; O’Byrne C.; Abee T.; Johansson J.; Poolman B. Super-Resolving Microscopy Reveals the Localizations and Movement Dynamics of Stressosome Proteins in Listeria Monocytogenes. Commun. Biol. 2023, 6 (1), 51 10.1038/s42003-023-04423-y. 36641529 PMC9840623

[ref175] Florea M. Aging and Immortality in Unicellular Species. Mech. Ageing Dev. 2017, 167, 5–15. 10.1016/j.mad.2017.08.006. 28844968

[ref176] Mika J. T.; Schavemaker P. E.; Krasnikov V.; Poolman B. Impact of Osmotic Stress on Protein Diffusion in *L Actococcus Lactis*: Protein Diffusion in *L*. Lactis. Mol. Microbiol. 2014, 94 (4), 857–870. 10.1111/mmi.12800. 25244659

[ref177] Alsallaq R.; Zhou H.-X. Electrostatic Rate Enhancement and Transient Complex of Protein–Protein Association. Proteins Struct. Funct. Bioinforma. 2008, 71 (1), 320–335. 10.1002/prot.21679. PMC3526769 17932929

[ref178] Schreiber G.; Fersht A. R. Interaction of Barnase with Its Polypeptide Inhibitor Barstar Studied by Protein Engineering. Biochemistry 1993, 32 (19), 5145–5150. 10.1021/bi00070a025. 8494892

[ref179] Wallis R.; Moore G. R.; James R.; Kleanthous C. Protein-Protein Interactions in Colicin E9 DNase-Immunity Protein Complexes. 1. Diffusion-Controlled Association and Femtomolar Binding for the Cognate Complex. Biochemistry 1995, 34 (42), 13743–13750. 10.1021/bi00042a004. 7577966

[ref180] Klumpp S.; Scott M.; Pedersen S.; Hwa T. Molecular Crowding Limits Translation and Cell Growth. Proc. Natl. Acad. Sci. U. S. A. 2013, 110 (42), 16754–16759. 10.1073/pnas.1310377110. 24082144 PMC3801028

[ref181] Zhang G.; Fedyunin I.; Miekley O.; Valleriani A.; Moura A.; Ignatova Z. Global and Local Depletion of Ternary Complex Limits Translational Elongation. Nucleic Acids Res. 2010, 38 (14), 4778–4787. 10.1093/nar/gkq196. 20360046 PMC2919707

[ref182] Loose M.; Kruse K.; Schwille P. Protein Self-Organization: Lessons from the Min System. Annu. Rev. Biophys. 2011, 40 (1), 315–336. 10.1146/annurev-biophys-042910-155332. 21545286

[ref183] McGuffee S. R.; Elcock A. H. Diffusion, Crowding & Protein Stability in a Dynamic Molecular Model of the Bacterial Cytoplasm. PLoS Comput. Biol. 2010, 6 (3), e1000694 10.1371/journal.pcbi.1000694. 20221255 PMC2832674

[ref184] Lewis P. J.; Thaker S. D.; Errington J. Compartmentalization of Transcription and Translation in Bacillus Subtilis. EMBO J. 2000, 19 (4), 710–718. 10.1093/emboj/19.4.710. 10675340 PMC305609

[ref185] Van Gijtenbeek L. A.; Robinson A.; Van Oijen A. M.; Poolman B.; Kok J. On the Spatial Organization of mRNA, Plasmids, and Ribosomes in a Bacterial Host Overexpressing Membrane Proteins. PLOS Genet. 2016, 12 (12), e1006523 10.1371/journal.pgen.1006523. 27977669 PMC5201305

[ref186] Odijk T. Osmotic Compaction of Supercoiled DNA into a Bacterial Nucleoid. Biophys. Chem. 1998, 73 (1–2), 23–29. 10.1016/S0301-4622(98)00115-X. 9697298

[ref187] Zimmerman S. B. Shape and Compaction of Escherichia Coli Nucleoids. J. Struct. Biol. 2006, 156 (2), 255–261. 10.1016/j.jsb.2006.03.022. 16697220

[ref188] Murphy L. D.; Zimmerman S. B. Condensation and Cohesion of λ DNA in Cell Extracts and Other Media: Implications for the Structure and Function of DNA in Prokaryotes. Biophys. Chem. 1995, 57 (1), 71–92. 10.1016/0301-4622(95)00047-2. 8534838

[ref189] Bailoni E.; Partipilo M.; Coenradij J.; Grundel D. A. J.; Slotboom D. J.; Poolman B. Minimal Out-of-Equilibrium Metabolism for Synthetic Cells: A Membrane Perspective. ACS Synth. Biol. 2023, 12 (4), 922–946. 10.1021/acssynbio.3c00062. 37027340 PMC10127287

[ref190] Lynch M.; Marinov G. K. The Bioenergetic Costs of a Gene. Proc. Natl. Acad. Sci. U. S. A. 2015, 112 (51), 15690–15695. 10.1073/pnas.1514974112. 26575626 PMC4697398

[ref191] Kaljević J.; Saaki T. N. V.; Govers S. K.; Remy O.; Van Raaphorst R.; Lamot T.; Laloux G. Chromosome Choreography during the Non-Binary Cell Cycle of a Predatory Bacterium. Curr. Biol. 2021, 31 (17), 3707–3720.e5. 10.1016/j.cub.2021.06.024. 34256020 PMC8445325

[ref192] Munder M. C.; Midtvedt D.; Franzmann T.; Nüske E.; Otto O.; Herbig M.; Ulbricht E.; Müller P.; Taubenberger A.; Maharana S.; et al. A pH-Driven Transition of the Cytoplasm from a Fluid- to a Solid-like State Promotes Entry into Dormancy. eLife 2016, 5, e09347 10.7554/eLife.09347. 27003292 PMC4850707

[ref193] Bakshi S.; Choi H.; Mondal J.; Weisshaar J. C. Time-Dependent Effects of Transcription- and Translation-Halting Drugs on the Spatial Distributions of the *E Scherichia Coli* Chromosome and Ribosomes: Time-Dependent Drug Effects on *E. Coli* Chromosome Spatial Distribution. Mol. Microbiol. 2014, 94 (4), 871–887. 10.1111/mmi.12805. 25250841 PMC4227943

[ref194] Pittas T.; Zuo W.; Boersma A. J. Cell Wall Damage Increases Macromolecular Crowding Effects in the Escherichia Coli Cytoplasm. iScience 2023, 26 (4), 106367 10.1016/j.isci.2023.106367. 37009215 PMC10064245

[ref195] Wlodarski M.; Mancini L.; Raciti B.; Sclavi B.; Lagomarsino M. C.; Cicuta P. Cytosolic Crowding Drives the Dynamics of Both Genome and Cytosol in Escherichia Coli Challenged with Sub-Lethal Antibiotic Treatments. iScience 2020, 23 (10), 101560 10.1016/j.isci.2020.101560. 33083729 PMC7522891

[ref196] Zhu Y.; Mohapatra S.; Weisshaar J. C. Rigidification of the *Escherichia Coli* Cytoplasm by the Human Antimicrobial Peptide LL-37 Revealed by Superresolution Fluorescence Microscopy. Proc. Natl. Acad. Sci. U. S. A. 2019, 116 (3), 1017–1026. 10.1073/pnas.1814924116. 30598442 PMC6338858

[ref197] Mikhailov A. S.; Kapral R. Hydrodynamic Collective Effects of Active Protein Machines in Solution and Lipid Bilayers. Proc. Natl. Acad. Sci. U. S. A. 2015, 112 ( (28), ), E3639–E3644 10.1073/pnas.1506825112. 26124140 PMC4507222

[ref198] Patel A.; Malinovska L.; Saha S.; Wang J.; Alberti S.; Krishnan Y.; Hyman A. A. ATP as a Biological Hydrotrope. Science 2017, 356 (6339), 753–756. 10.1126/science.aaf6846. 28522535

[ref199] He Y.; Kang J.; Song J. ATP Antagonizes the Crowding-Induced Destabilization of the Human Eye-Lens Protein γS-Crystallin. Biochem. Biophys. Res. Commun. 2020, 526 (4), 1112–1117. 10.1016/j.bbrc.2020.04.014. 32307080

[ref200] Pandey M. P.; Sasidharan S.; Raghunathan V. A.; Khandelia H. Molecular Mechanism of Hydrotropic Properties of GTP and ATP. J. Phys. Chem. B 2022, 126 (42), 8486–8494. 10.1021/acs.jpcb.2c06077. 36251789

[ref201] Nishizawa M.; Walinda E.; Morimoto D.; Kohn B.; Scheler U.; Shirakawa M.; Sugase K. Effects of Weak Nonspecific Interactions with ATP on Proteins. J. Am. Chem. Soc. 2021, 143 (31), 11982–11993. 10.1021/jacs.0c13118. 34338526

[ref202] Golestanian R. Enhanced Diffusion of Enzymes That Catalyze Exothermic Reactions. Phys. Rev. Lett. 2015, 115 (10), 108102 10.1103/PhysRevLett.115.108102. 26382704

[ref203] Riedel C.; Gabizon R.; Wilson C. A. M.; Hamadani K.; Tsekouras K.; Marqusee S.; Pressé S.; Bustamante C. The Heat Released during Catalytic Turnover Enhances the Diffusion of an Enzyme. Nature 2015, 517 (7533), 227–230. 10.1038/nature14043. 25487146 PMC4363105

[ref204] Sengupta S.; Dey K. K.; Muddana H. S.; Tabouillot T.; Ibele M. E.; Butler P. J.; Sen A. Enzyme Molecules as Nanomotors. J. Am. Chem. Soc. 2013, 135 (4), 1406–1414. 10.1021/ja3091615. 23308365

[ref205] Chen Z.; Shaw A.; Wilson H.; Woringer M.; Darzacq X.; Marqusee S.; Wang Q.; Bustamante C. Single-Molecule Diffusometry Reveals No Catalysis-Induced Diffusion Enhancement of Alkaline Phosphatase as Proposed by FCS Experiments. Proc. Natl. Acad. Sci. U. S. A. 2020, 117 (35), 21328–21335. 10.1073/pnas.2006900117. 32817484 PMC7474647

[ref206] Wang L.; Guo P.; Jin D.; Peng Y.; Sun X.; Chen Y.; Liu X.; Chen W.; Wang W.; Yan X.; Ma X. Enzyme-Powered Tubular Microrobotic Jets as Bioinspired Micropumps for Active Transmembrane Drug Transport. ACS Nano 2023, 17 (5), 5095–5107. 10.1021/acsnano.3c00291. 36861648

[ref207] Hołówka J.; Zakrzewska-Czerwińska J. Nucleoid Associated Proteins: The Small Organizers That Help to Cope With Stress. Front. Microbiol. 2020, 11, 590 10.3389/fmicb.2020.00590. 32373086 PMC7177045

[ref208] Joyeux M. Organization of the Bacterial Nucleoid by DNA-Bridging Proteins and Globular Crowders. Front. Microbiol. 2023, 14, 1116776 10.3389/fmicb.2023.1116776. 36925468 PMC10011147

[ref209] Worcel A.; Burgi E. On the Structure of the Folded Chromosome of Escherichia Coli. J. Mol. Biol. 1972, 71 (2), 127–147. 10.1016/0022-2836(72)90342-7. 4564477

[ref210] Kavenoff R.; Bowen B. C. Electron Microscopy of Membrane-Free Folded Chromosomes from Escherichia Coli. Chromosoma 1976, 59 (2), 89–101. 10.1007/BF00328479. 795620

[ref211] Cunha S.; Woldringh C. L.; Odijk T. Polymer-Mediated Compaction and Internal Dynamics of Isolated Escherichia Coli Nucleoids. J. Struct. Biol. 2001, 136 (1), 53–66. 10.1006/jsbi.2001.4420. 11858707

[ref212] De Vries R. DNA Condensation in Bacteria: Interplay between Macromolecular Crowding and Nucleoid Proteins. Biochimie 2010, 92 (12), 1715–1721. 10.1016/j.biochi.2010.06.024. 20615449

[ref213] Pelletier J.; Halvorsen K.; Ha B.-Y.; Paparcone R.; Sandler S. J.; Woldringh C. L.; Wong W. P.; Jun S. Physical Manipulation of the *Escherichia Coli* Chromosome Reveals Its Soft Nature. Proc. Natl. Acad. Sci. U. S. A. 2012, 109 ( (40), ), E2649–E2656 10.1073/pnas.1208689109. 22984156 PMC3479577

[ref214] Wang X.; Llopis P. M.; Rudner D. Z. Organization and Segregation of Bacterial Chromosomes. Nat. Rev. Genet. 2013, 14 (3), 191–203. 10.1038/nrg3375. 23400100 PMC3869393

[ref215] Gray W. T.; Govers S. K.; Xiang Y.; Parry B. R.; Campos M.; Kim S.; Jacobs-Wagner C. Nucleoid Size Scaling and Intracellular Organization of Translation across Bacteria. Cell 2019, 177 (6), 1632–1648.e20. 10.1016/j.cell.2019.05.017. 31150626 PMC6629263

[ref216] Benedetti F.; Japaridze A.; Dorier J.; Racko D.; Kwapich R.; Burnier Y.; Dietler G.; Stasiak A. Effects of Physiological Self-Crowding of DNA on Shape and Biological Properties of DNA Molecules with Various Levels of Supercoiling. Nucleic Acids Res. 2015, 43 (4), 2390–2399. 10.1093/nar/gkv055. 25653164 PMC4344501

[ref217] Nonejuie P.; Burkart M.; Pogliano K.; Pogliano J. Bacterial Cytological Profiling Rapidly Identifies the Cellular Pathways Targeted by Antibacterial Molecules. Proc. Natl. Acad. Sci. U. S. A. 2013, 110 (40), 16169–16174. 10.1073/pnas.1311066110. 24046367 PMC3791758

[ref218] Trojanowski D.; Kołodziej M.; Hołówka J.; Müller R.; Zakrzewska-Czerwińska J. Watching DNA Replication Inhibitors in Action: Exploiting Time-Lapse Microfluidic Microscopy as a Tool for Target-Drug Interaction Studies in *Mycobacterium*. Antimicrob. Agents Chemother. 2019, 63 (10), e00739–19. 10.1128/AAC.00739-19. 31383667 PMC6761567

[ref219] Kaval K. G.; Chimalapati S.; Siegel S. D.; Garcia N.; Jaishankar J.; Dalia A. B.; Orth K. Membrane-Localized Expression, Production and Assembly of Vibrio Parahaemolyticus T3SS2 Provides Evidence for Transertion. Nat. Commun. 2023, 14 (1), 1178 10.1038/s41467-023-36762-z. 36859532 PMC9977878

[ref220] Trojanowski D.; Hołówka J.; Zakrzewska-Czerwińska J. Where and When Bacterial Chromosome Replication Starts: A Single Cell Perspective. Front. Microbiol. 2018, 9, 2819 10.3389/fmicb.2018.02819. 30534115 PMC6275241

[ref221] Feric M.; Misteli T. Phase Separation in Genome Organization across Evolution. Trends Cell Biol. 2021, 31 (8), 671–685. 10.1016/j.tcb.2021.03.001. 33771451 PMC8286288

[ref222] Joyeux M. A Segregative Phase Separation Scenario of the Formation of the Bacterial Nucleoid. Soft Matter 2018, 14 (36), 7368–7381. 10.1039/C8SM01205A. 30204212

[ref223] Finkelstein I. J.; Greene E. C. Molecular Traffic Jams on DNA. Annu. Rev. Biophys. 2013, 42 (1), 241–263. 10.1146/annurev-biophys-083012-130304. 23451891 PMC3651777

[ref224] Tabaka M.; Kalwarczyk T.; Holyst R. Quantitative Influence of Macromolecular Crowding on Gene Regulation Kinetics. Nucleic Acids Res. 2014, 42 (2), 727–738. 10.1093/nar/gkt907. 24121687 PMC3902910

[ref225] Stojkova P.; Spidlova P.; Stulik J. Nucleoid-Associated Protein HU: A Lilliputian in Gene Regulation of Bacterial Virulence. Front. Cell. Infect. Microbiol. 2019, 9, 159 10.3389/fcimb.2019.00159. 31134164 PMC6523023

[ref226] Gupta A.; Joshi A.; Arora K.; Mukhopadhyay S.; Guptasarma P. The Bacterial Nucleoid-Associated Proteins, HU and Dps, Condense DNA into Context-Dependent Biphasic or Multiphasic Complex Coacervates. J. Biol. Chem. 2023, 299 (5), 104637 10.1016/j.jbc.2023.104637. 36963493 PMC10141540

[ref227] Ali Azam T.; Iwata A.; Nishimura A.; Ueda S.; Ishihama A. Growth Phase-Dependent Variation in Protein Composition of the *Escherichia Coli* Nucleoid. J. Bacteriol. 1999, 181 (20), 6361–6370. 10.1128/JB.181.20.6361-6370.1999. 10515926 PMC103771

[ref228] Portz B.; Lu F.; Gibbs E. B.; Mayfield J. E.; Rachel Mehaffey M.; Zhang Y. J.; Brodbelt J. S.; Showalter S. A.; Gilmour D. S. Structural Heterogeneity in the Intrinsically Disordered RNA Polymerase II C-Terminal Domain. Nat. Commun. 2017, 8 (1), 15231 10.1038/ncomms15231. 28497792 PMC5437306

[ref229] Stracy M.; Lesterlin C.; Garza De Leon F.; Uphoff S.; Zawadzki P.; Kapanidis A. N. Live-Cell Superresolution Microscopy Reveals the Organization of RNA Polymerase in the Bacterial Nucleoid. Proc. Natl. Acad. Sci. U. S. A. 2015, 112 ( (32), ), 10.1073/pnas.1507592112. PMC4538611 26224838

[ref230] Bakshi S.; Dalrymple R. M.; Li W.; Choi H.; Weisshaar J. C. Partitioning of RNA Polymerase Activity in Live Escherichia Coli from Analysis of Single-Molecule Diffusive Trajectories. Biophys. J. 2013, 105 (12), 2676–2686. 10.1016/j.bpj.2013.10.024. 24359739 PMC3882475

[ref231] Giacometti S. I.; MacRae M. R.; Dancel-Manning K.; Bhabha G.; Ekiert D. C. Lipid Transport Across Bacterial Membranes. Annu. Rev. Cell Dev. Biol. 2022, 38 (1), 125–153. 10.1146/annurev-cellbio-120420-022914. 35850151 PMC12981311

[ref232] Rojas E. R.; Billings G.; Odermatt P. D.; Auer G. K.; Zhu L.; Miguel A.; Chang F.; Weibel D. B.; Theriot J. A.; Huang K. C. The Outer Membrane Is an Essential Load-Bearing Element in Gram-Negative Bacteria. Nature 2018, 559 (7715), 617–621. 10.1038/s41586-018-0344-3. 30022160 PMC6089221

[ref233] Sun J.; Rutherford S. T.; Silhavy T. J.; Huang K. C. Physical Properties of the Bacterial Outer Membrane. Nat. Rev. Microbiol. 2022, 20 (4), 236–248. 10.1038/s41579-021-00638-0. 34732874 PMC8934262

[ref234] Bramkamp M. Fluidity Is the Way to Life: Lipid Phase Separation in Bacterial Membranes. EMBO J. 2022, 41 (5), e110737 10.15252/embj.2022110737. 35143047 PMC8886535

[ref235] Sezgin E.; Levental I.; Mayor S.; Eggeling C. The Mystery of Membrane Organization: Composition, Regulation and Roles of Lipid Rafts. Nat. Rev. Mol. Cell Biol. 2017, 18 (6), 361–374. 10.1038/nrm.2017.16. 28356571 PMC5500228

[ref236] Nickels J. D.; Chatterjee S.; Stanley C. B.; Qian S.; Cheng X.; Myles D. A. A.; Standaert R. F.; Elkins J. G.; Katsaras J. The in Vivo Structure of Biological Membranes and Evidence for Lipid Domains. PLOS Biol. 2017, 15 (5), e2002214 10.1371/journal.pbio.2002214. 28542493 PMC5441578

[ref237] Pajerski W.; Ochonska D.; Brzychczy-Wloch M.; Indyka P.; Jarosz M.; Golda-Cepa M.; Sojka Z.; Kotarba A. Attachment Efficiency of Gold Nanoparticles by Gram-Positive and Gram-Negative Bacterial Strains Governed by Surface Charges. J. Nanoparticle Res. 2019, 21 (8), 186 10.1007/s11051-019-4617-z.

[ref238] Gohrbandt M.; Lipski A.; Grimshaw J. W.; Buttress J. A.; Baig Z.; Herkenhoff B.; Walter S.; Kurre R.; Deckers-Hebestreit G.; Strahl H. Low Membrane Fluidity Triggers Lipid Phase Separation and Protein Segregation in Living Bacteria. EMBO J. 2022, 41 (5), e109800 10.15252/embj.2021109800. 35037270 PMC8886542

[ref239] Mukhopadhyay R.; Huang K. C.; Wingreen N. S. Lipid Localization in Bacterial Cells through Curvature-Mediated Microphase Separation. Biophys. J. 2008, 95 (3), 1034–1049. 10.1529/biophysj.107.126920. 18390605 PMC2479595

[ref240] Kawai F.; Shoda M.; Harashima R.; Sadaie Y.; Hara H.; Matsumoto K. Cardiolipin Domains in *Bacillus Subtilis* Marburg Membranes. J. Bacteriol. 2004, 186 (5), 1475–1483. 10.1128/JB.186.5.1475-1483.2004. 14973018 PMC344405

[ref241] Romantsov T.; Helbig S.; Culham D. E.; Gill C.; Stalker L.; Wood J. M. Cardiolipin Promotes Polar Localization of Osmosensory Transporter ProP in *Escherichia Coli*: Cardiolipin and Osmoregulation in *Escherichia Coli*. Mol. Microbiol. 2007, 64 (6), 1455–1465. 10.1111/j.1365-2958.2007.05727.x. 17504273

[ref242] Benn G.; Mikheyeva I. V.; Inns P. G.; Forster J. C.; Ojkic N.; Bortolini C.; Ryadnov M. G.; Kleanthous C.; Silhavy T. J.; Hoogenboom B. W. Phase Separation in the Outer Membrane of *Escherichia Coli*. Proc. Natl. Acad. Sci. U. S. A. 2021, 118 (44), e2112237118 10.1073/pnas.2112237118. 34716276 PMC8612244

[ref243] Vaara M. Antibiotic-Supersusceptible Mutants of Escherichia Coli and Salmonella Typhimurium. Antimicrob. Agents Chemother. 1993, 37 (11), 2255–2260. 10.1128/AAC.37.11.2255. 8285603 PMC192375

[ref244] Fu L.; Li X.; Zhang S.; Dong Y.; Fang W.; Gao L. Polymyxins Induce Lipid Scrambling and Disrupt the Homeostasis of Gram-Negative Bacteria Membrane. Biophys. J. 2022, 121 (18), 3486–3498. 10.1016/j.bpj.2022.08.007. 35964158 PMC9515121

[ref245] Banjade S.; Rosen M. K. Phase Transitions of Multivalent Proteins Can Promote Clustering of Membrane Receptors. eLife 2014, 3, e04123 10.7554/eLife.04123. 25321392 PMC4238058

[ref246] Heinkel F.; Abraham L.; Ko M.; Chao J.; Bach H.; Hui L. T.; Li H.; Zhu M.; Ling Y. M.; Rogalski J. C.; Scurll J.; Bui J. M.; Mayor T.; Gold M. R.; Chou K. C.; Av-Gay Y.; McIntosh L. P.; Gsponer J. Phase Separation and Clustering of an ABC Transporter in Mycobacterium Tuberculosis. Proc. Natl. Acad. Sci. U A 2019, 116 (33), 16326–16331. 10.1073/pnas.1820683116. PMC6697873 31366629

[ref247] Monterroso B.; Robles-Ramos M. Á.; Sobrinos-Sanguino M.; Luque-Ortega J. R.; Alfonso C.; Margolin W.; Rivas G.; Zorrilla S. Bacterial Division Ring Stabilizing ZapA versus Destabilizing SlmA Modulate FtsZ Switching between Biomolecular Condensates and Polymers. Open Biol. 2023, 13 (3), 220324 10.1098/rsob.220324. 36854378 PMC9974302

[ref248] Monterroso B.; Zorrilla S.; Sobrinos-Sanguino M.; Robles-Ramos M. A.; Lopez-Alvarez M.; Margolin W.; Keating C. D.; Rivas G. Bacterial FtsZ Protein Forms Phase-Separated Condensates with Its Nucleoid-Associated Inhibitor SlmA. EMBO Rep 2019, 20 (1), e45946 10.15252/embr.201845946. 30523075 PMC6322363

[ref249] Tan W.; Cheng S.; Li Y.; Li X.-Y.; Lu N.; Sun J.; Tang G.; Yang Y.; Cai K.; Li X.; et al. Phase Separation Modulates the Assembly and Dynamics of a Polarity-Related Scaffold-Signaling Hub. Nat. Commun. 2022, 13 (1), 7181 10.1038/s41467-022-35000-2. 36418326 PMC9684454

[ref250] Zhao T.; Liu Y.; Wang Z.; He R.; Xiang Zhang J.; Xu F.; Lei M.; Deci M. B.; Nguyen J.; Bianco P. R. Super-resolution Imaging Reveals Changes in *Escherichia Coli* SSB Localization in Response to DNA Damage. Genes Cells 2019, 24 (12), 814–826. 10.1111/gtc.12729. 31638317 PMC7065570

[ref251] Adams D. W.; Wu L. J.; Errington J. Nucleoid Occlusion Protein N Oc Recruits DNA to the Bacterial Cell Membrane. EMBO J. 2015, 34 (4), 491–501. 10.15252/embj.201490177. 25568309 PMC4331003

[ref252] Robles-Ramos M. A.; Margolin W.; Sobrinos-Sanguino M.; Alfonso C.; Rivas G.; Monterroso B.; Zorrilla S. The Nucleoid Occlusion Protein SlmA Binds to Lipid Membranes. mBio 2020, 11, e02094–20. 10.1128/mBio.02094-20. 32873767 PMC7468209

[ref253] Harami G. M.; Kovács Z. J.; Pancsa R.; Pálinkás J.; Baráth V.; Tárnok K.; Málnási-Csizmadia A.; Kovács M. Phase Separation by ssDNA Binding Protein Controlled via Protein–protein and protein–DNA Interactions. Proc. Natl. Acad. Sci. U. S. A. 2020, 117 (42), 26206–26217. 10.1073/pnas.2000761117. 33020264 PMC7584906

[ref254] Paccione G.; Robles-Ramos M. Á.; Alfonso C.; Sobrinos-Sanguino M.; Margolin W.; Zorrilla S.; Monterroso B.; Rivas G. Lipid Surfaces and Glutamate Anions Enhance Formation of Dynamic Biomolecular Condensates Containing Bacterial Cell Division Protein FtsZ and Its DNA-Bound Regulator SlmA. Biochemistry 2022, 61 (22), 2482–2489. 10.1021/acs.biochem.2c00424. 36315857 PMC9670838

[ref255] Robles-Ramos M. A.; Zorrilla S.; Alfonso C.; Margolin W.; Rivas G.; Monterroso B. Assembly of Bacterial Cell Division Protein FtsZ into Dynamic Biomolecular Condensates. Biochim Biophys Acta Mol. Cell Res. 2021, 1868 (5), 118986 10.1016/j.bbamcr.2021.118986. 33581219 PMC8529516

[ref256] Erickson J. L.; Prautsch J.; Reynvoet F.; Niemeyer F.; Hause G.; Johnston I. G.; Schattat M. H. Stromule Geometry Allows Optimal Spatial Regulation of Organelle Interactions in the Quasi-2D Cytoplasm. Plant Cell Physiol. 2023, pcad098 10.1093/pcp/pcad098. 37658689 PMC11094753

[ref257] Ignatova Z.; Gierasch L. M. Inhibition of Protein Aggregation in Vitro and in Vivo by a Natural Osmoprotectant. Proc. Natl. Acad. Sci. U. S. A. 2006, 103 (36), 13357–13361. 10.1073/pnas.0603772103. 16899544 PMC1569168

[ref258] Wan Q.; Mouton S. N.; Veenhoff L. M.; Boersma A. J. A FRET-Based Method for Monitoring Structural Transitions in Protein Self-Organization. Cell Rep. Methods 2022, 2 (3), 100184 10.1016/j.crmeth.2022.100184. 35475219 PMC8960284

[ref259] Brangwynne C. P. Phase Transitions and Size Scaling of Membrane-Less Organelles. J. Cell Biol. 2013, 203 (6), 875–881. 10.1083/jcb.201308087. 24368804 PMC3871435

[ref260] Snead W. T.; Jalihal A. P.; Gerbich T. M.; Seim I.; Hu Z.; Gladfelter A. S. Membrane Surfaces Regulate Assembly of Ribonucleoprotein Condensates. Nat. Cell Biol. 2022, 24 (4), 461–470. 10.1038/s41556-022-00882-3. 35411085 PMC9035128

[ref261] Case L. B.; Zhang X.; Ditlev J. A.; Rosen M. K. Stoichiometry Controls Activity of Phase-Separated Clusters of Actin Signaling Proteins. Science 2019, 363 (6431), 1093–1097. 10.1126/science.aau6313. 30846599 PMC6784323

[ref262] Vale R. D. The Molecular Motor Toolbox for Intracellular Transport. Cell 2003, 112 (4), 467–480. 10.1016/S0092-8674(03)00111-9. 12600311

[ref263] Sittewelle M.; Royle S. J. Passive Diffusion Accounts for the Majority of Intracellular Nanovesicle Transport. Life Sci. Alliance 2024, 7 (1), e202302406 10.26508/lsa.202302406. 37857498 PMC10587482

[ref264] Ditlev J. A.; Vega A. R.; Köster D. V.; Su X.; Tani T.; Lakoduk A. M.; Vale R. D.; Mayor S.; Jaqaman K.; Rosen M. K. A Composition-Dependent Molecular Clutch between T Cell Signaling Condensates and Actin. eLife 2019, 8, e42695 10.7554/eLife.42695. 31268421 PMC6624021

[ref265] Uversky V. N. Intrinsically Disordered Proteins in Overcrowded Milieu: Membrane-Less Organelles, Phase Separation, and Intrinsic Disorder. Curr. Opin. Struct. Biol. 2017, 44, 18–30. 10.1016/j.sbi.2016.10.015. 27838525

[ref266] Sridharan S.; Hernandez-Armendariz A.; Kurzawa N.; Potel C. M.; Memon D.; Beltrao P.; Bantscheff M.; Huber W.; Cuylen-Haering S.; Savitski M. M. Systematic Discovery of Biomolecular Condensate-Specific Protein Phosphorylation. Nat. Chem. Biol. 2022, 18 (10), 1104–1114. 10.1038/s41589-022-01062-y. 35864335 PMC9512703

[ref267] Lin C.-W.; Nocka L. M.; Stinger B. L.; DeGrandchamp J. B.; Lew L. J. N.; Alvarez S.; Phan H. T.; Kondo Y.; Kuriyan J.; Groves J. T. A Two-Component Protein Condensate of the EGFR Cytoplasmic Tail and Grb2 Regulates Ras Activation by SOS at the Membrane. Proc. Natl. Acad. Sci. U. S. A. 2022, 119 (19), e2122531119 10.1073/pnas.2122531119. 35507881 PMC9181613

[ref268] López-Palacios T. P.; Andersen J. L. Kinase Regulation by Liquid–Liquid Phase Separation. Trends Cell Biol. 2023, 33, 649 10.1016/j.tcb.2022.11.009. 36528418 PMC10267292

[ref269] Lasker K.; von Diezmann L.; Zhou X.; Ahrens D. G.; Mann T. H.; Moerner W. E.; Shapiro L. Selective Sequestration of Signalling Proteins in a Membraneless Organelle Reinforces the Spatial Regulation of Asymmetry in Caulobacter Crescentus. Nat. Microbiol. 2020, 5 (3), 418–429. 10.1038/s41564-019-0647-7. 31959967 PMC7549192

[ref270] Nguemaha V.; Zhou H.-X. Liquid-Liquid Phase Separation of Patchy Particles Illuminates Diverse Effects of Regulatory Components on Protein Droplet Formation. Sci. Rep. 2018, 8 (1), 6728 10.1038/s41598-018-25132-1. 29712961 PMC5928213

[ref271] He S.; Chou H.-T.; Matthies D.; Wunder T.; Meyer M. T.; Atkinson N.; Martinez-Sanchez A.; Jeffrey P. D.; Port S. A.; Patena W.; et al. The Structural Basis of Rubisco Phase Separation in the Pyrenoid. Nat. Plants 2020, 6 (12), 1480–1490. 10.1038/s41477-020-00811-y. 33230314 PMC7736253

[ref272] Oltrogge L. M.; Chaijarasphong T.; Chen A. W.; Bolin E. R.; Marqusee S.; Savage D. F. Multivalent Interactions between CsoS2 and Rubisco Mediate α-Carboxysome Formation. Nat. Struct. Mol. Biol. 2020, 27 (3), 281–287. 10.1038/s41594-020-0387-7. 32123388 PMC7337323

[ref273] Wang H.; Yan X.; Aigner H.; Bracher A.; Nguyen N. D.; Hee W. Y.; Long B. M.; Price G. D.; Hartl F. U.; Hayer-Hartl M. Rubisco Condensate Formation by CcmM in β-Carboxysome Biogenesis. Nature 2019, 566 (7742), 131–135. 10.1038/s41586-019-0880-5. 30675061

[ref274] Basalla J. L.; Mak C. A.; Byrne J. A.; Ghalmi M.; Hoang Y.; Vecchiarelli A. G. Dissecting the Phase Separation and Oligomerization Activities of the Carboxysome Positioning Protein McdB. eLife 2023, 12, e81362 10.7554/eLife.81362. 37668016 PMC10554743

[ref275] Xing W.; Muhlrad D.; Parker R.; Rosen M. K. A Quantitative Inventory of Yeast P Body Proteins Reveals Principles of Composition and Specificity. eLife 2020, 9, e56525 10.7554/eLife.56525. 32553117 PMC7373430

[ref276] Al-Husini N.; Tomares D. T.; Bitar O.; Childers W. S.; Schrader J. M. Alpha-Proteobacterial RNA Degradosomes Assemble Liquid-Liquid Phase-Separated RNP Bodies. Mol. Cell 2018, 71 (6), 1027–1039 e14. 10.1016/j.molcel.2018.08.003. 30197298 PMC6151146

[ref277] Peskett T. R.; Rau F.; O’Driscoll J.; Patani R.; Lowe A. R.; Saibil H. R. A Liquid to Solid Phase Transition Underlying Pathological Huntingtin Exon1 Aggregation. Mol. Cell 2018, 70 (4), 588–601.e6. 10.1016/j.molcel.2018.04.007. 29754822 PMC5971205

[ref278] Ambadipudi S.; Biernat J.; Riedel D.; Mandelkow E.; Zweckstetter M. Liquid–Liquid Phase Separation of the Microtubule-Binding Repeats of the Alzheimer-Related Protein Tau. Nat. Commun. 2017, 8 (1), 275 10.1038/s41467-017-00480-0. 28819146 PMC5561136

[ref279] Patel A.; Lee H. O.; Jawerth L.; Maharana S.; Jahnel M.; Hein M. Y.; Stoynov S.; Mahamid J.; Saha S.; Franzmann T. M.; et al. Liquid-to-Solid Phase Transition of the ALS Protein FUS Accelerated by Disease Mutation. Cell 2015, 162 (5), 1066–1077. 10.1016/j.cell.2015.07.047. 26317470

[ref280] Lipiński W. P.; Visser B. S.; Robu I.; Fakhree M. A. A.; Lindhoud S.; Claessens M. M. A. E.; Spruijt E. Biomolecular Condensates Can Both Accelerate and Suppress Aggregation of α-Synuclein. Sci. Adv. 2022, 8 ( (48), ), eabq6495. 10.1126/sciadv.abq6495. 36459561 PMC10942789

[ref281] Hollembeak M. A. M. J. E.; Kurokawa M. Macromolecular Crowding: A Hidden Link between Cell Volume and Everything Else. Cell Physiol Biochem 2021, 55 (S1), 25–40. 10.33594/000000319. 33385320

[ref282] Oh S.; Lee C.; Yang W.; Li A.; Mukherjee A.; Basan M.; Ran C.; Yin W.; Tabin C. J.; Fu D.; Xie X. S.; Kirschner M. W. Protein and Lipid Mass Concentration Measurement in Tissues by Stimulated Raman Scattering Microscopy. Proc. Natl. Acad. Sci. U. S. A. 2022, 119 (17), e2117938119 10.1073/pnas.2117938119. 35452314 PMC9169924

[ref283] Cayley S.; Lewis B. A.; Guttman H. J.; Record M. T. Characterization of the Cytoplasm of Escherichia Coli K-12 as a Function of External Osmolarity: Implications for Protein-DNA Interactions in Vivo. J. Mol. Biol. 1991, 222 (2), 281–300. 10.1016/0022-2836(91)90212-O. 1960728

[ref284] Odermatt P. D.; Miettinen T. P.; Lemière J.; Kang J. H.; Bostan E.; Manalis S. R.; Huang K. C.; Chang F. Variations of Intracellular Density during the Cell Cycle Arise from Tip-Growth Regulation in Fission Yeast. Elife 2021, 10, e64901 10.7554/eLife.64901. 34100714 PMC8221806

[ref285] Liu X.; Oh S.; Kirschner M. W. The Uniformity and Stability of Cellular Mass Density in Mammalian Cell Culture. Front. Cell Dev. Biol. 2022, 10, 10.3389/fcell.2022.1017499. PMC9597509 36313562

[ref286] Li Y.; Chen M.; Hu J.; Sheng R.; Lin Q.; He X.; Guo M. Volumetric Compression Induces Intracellular Crowding to Control Intestinal Organoid Growth via Wnt/β-Catenin Signaling. Cell Stem Cell 2021, 28 (1), 63–78.e7. 10.1016/j.stem.2020.09.012. 33053374 PMC7796961

[ref287] Pittas T.; Boersma A. J. Self-Association of a Nucleoid-Binding Protein Increases with Macromolecular Crowding in *Escherichia Coli*. bioRxiv 2023, 10.1101/2023.02.23.529735.

[ref288] Burg M. B.; Ferraris J. D.; Dmitrieva N. I. Cellular Response to Hyperosmotic Stresses. Physiol. Rev. 2007, 87 (4), 1441–1474. 10.1152/physrev.00056.2006. 17928589

[ref289] Hoffmann E. K.; Lambert I. H.; Pedersen S. F. Physiology of Cell Volume Regulation in Vertebrates. Physiol. Rev. 2009, 89 (1), 193–277. 10.1152/physrev.00037.2007. 19126758

[ref290] Boyd-Shiwarski C. R.; Shiwarski D. J.; Griffiths S. E.; Beacham R. T.; Norrell L.; Morrison D. E.; Wang J.; Mann J.; Tennant W.; Anderson E. N.; et al. Kinases Sense Molecular Crowding and Rescue Cell Volume via Phase Separation. Cell 2022, 185 (24), 4488–4506.e20. 10.1016/j.cell.2022.09.042. 36318922 PMC9699283

[ref291] Rafiei N.; Cordova M.; Navarre W. W.; Milstein J. N. Growth Phase-Dependent Chromosome Condensation and Heat-Stable Nucleoid-Structuring Protein Redistribution in Escherichia Coli under Osmotic Stress. J. Bacteriol. 2019, 201 ( (23), ), 10.1128/JB.00469-19. PMC6832063 31481544

[ref292] Wirth A. J.; Gruebele M. Quinary Protein Structure and the Consequences of Crowding in Living Cells: Leaving the Test-tube Behind. BioEssays 2013, 35 (11), 984–993. 10.1002/bies.201300080. 23943406

[ref293] Danielsson J.; Mu X.; Lang L.; Wang H.; Binolfi A.; Theillet F.-X.; Bekei B.; Logan D. T.; Selenko P.; Wennerström H.; Oliveberg M. Thermodynamics of Protein Destabilization in Live Cells. Proc. Natl. Acad. Sci. U. S. A. 2015, 112 (40), 12402–12407. 10.1073/pnas.1511308112. 26392565 PMC4603463

[ref294] Dayel M. J.; Hom E. F.; Verkman A. S. Diffusion of Green Fluorescent Protein in the Aqueous-Phase Lumen of Endoplasmic Reticulum. Biophys. J. 1999, 76 (5), 2843–2851. 10.1016/S0006-3495(99)77438-2. 10233100 PMC1300255

[ref295] Leeb S.; Sörensen T.; Yang F.; Mu X.; Oliveberg M.; Danielsson J. Diffusive Protein Interactions in Human versus Bacterial Cells. Curr. Res. Struct. Biol. 2020, 2, 68–78. 10.1016/j.crstbi.2020.04.002. 34235470 PMC8244477

[ref296] Mu X.; Choi S.; Lang L.; Mowray D.; Dokholyan N. V.; Danielsson J.; Oliveberg M. Physicochemical Code for Quinary Protein Interactions in Escherichia Coli. Proc. Natl. Acad. Sci. U. S. A. 2017, 114 (23), E4556–E4563. 10.1073/pnas.1621227114. 28536196 PMC5468600

[ref297] Schweke H.; Levin T.; Pacesa M.; Goverde C. A.; Kumar P.; Duhoo Y.; Dornfeld L. J.; Dubreuil B.; Georgeon S.; et al. An Atlas of Protein Homo-Oligomerization across Domains of Life. bioRxiv 2023, 10.1101/2023.06.09.544317. 38325366

[ref298] Holland I. B. Rise and Rise of the ABC Transporter Families. Res. Microbiol. 2019, 170 (8), 304–320. 10.1016/j.resmic.2019.08.004. 31442613

[ref299] Thomas C.; Aller S. G.; Beis K.; Carpenter E. P.; Chang G.; Chen L.; Dassa E.; Dean M.; Duong Van Hoa F.; Ekiert D.; et al. Structural and Functional Diversity Calls for a New Classification of ABC Transporters. FEBS Lett. 2020, 594 (23), 3767–3775. 10.1002/1873-3468.13935. 32978974 PMC8386196

[ref300] Rospert S.; Dubaquié Y.; Gautschi M. Nascent-Polypeptide-Associated Complex. Cell. Mol. Life Sci. CMLS 2002, 59 (10), 1632–1639. 10.1007/PL00012490. 12475173 PMC11337418

[ref301] Xie Y.; Liu T.; Gresham D.; Holt L. J.. mRNA Condensation Fluidizes the Cytoplasm. bioRxiv 2023, 10.1101/2023.05.30.542963.

[ref302] Marini G.; Nüske E.; Leng W.; Alberti S.; Pigino G. Reorganization of Budding Yeast Cytoplasm upon Energy Depletion. Mol. Biol. Cell 2020, 31 (12), 1232–1245. 10.1091/mbc.E20-02-0125. 32293990 PMC7353153

[ref303] Mouton S. N.; Thaller D. J.; Crane M. M.; Rempel I. L.; Terpstra O. T.; Steen A.; Kaeberlein M.; Lusk C. P.; Boersma A. J.; Veenhoff L. M. A Physicochemical Perspective of Aging from Single-Cell Analysis of pH, Macromolecular and Organellar Crowding in Yeast. eLife 2020, 9, e54707 10.7554/eLife.54707. 32990592 PMC7556870

[ref304] Zuo W.; Huang M.-R.; Schmitz F.; Boersma A. J.. Genetically-Encoded Probes to Determine Nonspecific Hydrophobic and Electrostatic Binding in Cells. bioRxiv 2023, 10.1101/2023.06.27.546658.

[ref305] Sengupta R.; Pantel A.; Cheng X.; Shkel I.; Peran I.; Stenzoski N.; Raleigh D. P.; Record M. T. J. Positioning the Intracellular Salt Potassium Glutamate in the Hofmeister Series by Chemical Unfolding Studies of NTL9. Biochemistry 2016, 55 (15), 2251–2259. 10.1021/acs.biochem.6b00173. 27054379 PMC4837102

[ref306] Kozlov A. G.; Cheng X.; Zhang H.; Shinn M. K.; Weiland E.; Nguyen B.; Shkel I. A.; Zytkiewicz E.; Finkelstein I. J.; Record M. T.; Lohman T. M. How Glutamate Promotes Liquid-Liquid Phase Separation and DNA Binding Cooperativity of E. Coli SSB Protein. J. Mol. Biol. 2022, 434 (9), 167562 10.1016/j.jmb.2022.167562. 35351518 PMC9400470

[ref307] Murthy A. C.; Dignon G. L.; Kan Y.; Zerze G. H.; Parekh S. H.; Mittal J.; Fawzi N. L. Molecular Interactions Underlying Liquid-Liquid Phase Separation of the FUS Low-Complexity Domain. Nat. Struct. Mol. Biol. 2019, 26 (7), 637–648. 10.1038/s41594-019-0250-x. 31270472 PMC6613800

[ref308] Timasheff S. N. Protein-Solvent Preferential Interactions, Protein Hydration, and the Modulation of Biochemical Reactions by Solvent Components. Proc. Natl. Acad. Sci. U. S. A. 2002, 99 (15), 9721–9726. 10.1073/pnas.122225399. 12097640 PMC124992

[ref309] Yancey P. H. Organic Osmolytes as Compatible, Metabolic and Counteracting Cytoprotectants in High Osmolarity and Other Stresses. J. Exp. Biol. 2005, 208 (15), 2819–2830. 10.1242/jeb.01730. 16043587

[ref310] Sachs G.; Kraut J. A.; Wen Y.; Feng J.; Scott D. R. Urea Transport in Bacteria: Acid Acclimation by Gastric Helicobacter Spp. J. Membr. Biol. 2006, 212 (2), 71–82. 10.1007/s00232-006-0867-7. 17264989

[ref311] Cinar H.; Winter R. The Effects of Cosolutes and Crowding on the Kinetics of Protein Condensate Formation Based on Liquid–Liquid Phase Separation: A Pressure-Jump Relaxation Study. Sci. Rep. 2020, 10 (1), 17245 10.1038/s41598-020-74271-x. 33057154 PMC7566631

[ref312] Youngren B.; Nielsen H. J.; Jun S.; Austin S. The Multifork *Escherichia Coli* Chromosome Is a Self-Duplicating and Self-Segregating Thermodynamic Ring Polymer. Genes Dev. 2014, 28 (1), 71–84. 10.1101/gad.231050.113. 24395248 PMC3894414

[ref313] Marczynski G. T.; Petit K.; Patel P. Crosstalk Regulation Between Bacterial Chromosome Replication and Chromosome Partitioning. Front. Microbiol. 2019, 10, 279 10.3389/fmicb.2019.00279. 30863373 PMC6399470

[ref314] Soler-Bistué A.; Aguilar-Pierlé S.; Garcia-Garcerá M.; Val M.-E.; Sismeiro O.; Varet H.; Sieira R.; Krin E.; Skovgaard O.; Comerci D. J.; Rocha E. P. C.; Mazel D. Macromolecular Crowding Links Ribosomal Protein Gene Dosage to Growth Rate in Vibrio Cholerae. BMC Biol. 2020, 18 (1), 43 10.1186/s12915-020-00777-5. 32349767 PMC7191768

[ref315] Akabayov B.; Akabayov S. R.; Lee S.-J.; Wagner G.; Richardson C. C. Impact of Macromolecular Crowding on DNA Replication. Nat. Commun. 2013, 4 (1), 1615 10.1038/ncomms2620. 23511479 PMC3666333

[ref316] Fuller R. S.; Kaguni J. M.; Kornberg A. Enzymatic Replication of the Origin of the Escherichia Coli Chromosome. Proc. Natl. Acad. Sci. U. S. A. 1981, 78 (12), 7370–7374. 10.1073/pnas.78.12.7370. 6278471 PMC349268

[ref317] Zimmerman S. B.; Harrison B. Macromolecular Crowding Increases Binding of DNA Polymerase to DNA: An Adaptive Effect. Proc. Natl. Acad. Sci. U. S. A. 1987, 84 (7), 1871–1875. 10.1073/pnas.84.7.1871. 3550799 PMC304543

[ref318] Aranovich A.; Gdalevsky G. Y.; Cohen-Luria R.; Fishov I.; Parola A. H. Membrane-Catalyzed Nucleotide Exchange on DnaA. J. Biol. Chem. 2006, 281 (18), 12526–12534. 10.1074/jbc.M510266200. 16517983

[ref319] Aranovich A.; Braier-Marcovitz S.; Ansbacher E.; Granek R.; Parola A. H.; Fishov I. N-Terminal-Mediated Oligomerization of DnaA Drives the Occupancy-Dependent Rejuvenation of the Protein on the Membrane. Biosci. Rep. 2015, 35 (5), e00250 10.1042/BSR20150175. 26272946 PMC4721551

[ref320] Garner J.; Durrer P.; Kitchen J.; Brunner J.; Crooke E. Membrane-Mediated Release of Nucleotide from an Initiator of Chromosomal Replication, Escherichia Coli DnaA, Occurs with Insertion of a Distinct Region of the Protein into the Lipid Bilayer. J. Biol. Chem. 1998, 273 (9), 5167–5173. 10.1074/jbc.273.9.5167. 9478970

[ref321] Hou Y.; Kumar P.; Aggarwal M.; Sarkari F.; Wolcott K. M.; Chattoraj D. K.; Crooke E.; Saxena R. The Linker Domain of the Initiator DnaA Contributes to Its ATP Binding and Membrane Association in E. Coli Chromosomal Replication. Sci. Adv. 2022, 8 (40), eabq6657 10.1126/sciadv.abq6657. 36197974 PMC9534497

[ref322] Felczak M. M.; Simmons L. A.; Kaguni J. M. An Essential Tryptophan of Escherichia Coli DnaA Protein Functions in Oligomerization at the E. Coli Replication Origin. J. Biol. Chem. 2005, 280 (26), 24627–24633. 10.1074/jbc.M503684200. 15878847

[ref323] Mercier R.; Petit M.-A.; Schbath S.; Robin S.; El Karoui M.; Boccard F.; Espéli O. The MatP/matS Site-Specific System Organizes the Terminus Region of the E. Coli Chromosome into a Macrodomain. Cell 2008, 135 (3), 475–485. 10.1016/j.cell.2008.08.031. 18984159

[ref324] Gogou C.; Japaridze A.; Dekker C. Mechanisms for Chromosome Segregation in Bacteria. Front. Microbiol. 2021, 12, 685687 10.3389/fmicb.2021.685687. 34220773 PMC8242196

[ref325] Japaridze A.; Gogou C.; Kerssemakers J. W. J.; Nguyen H. M.; Dekker C. Direct Observation of Independently Moving Replisomes in Escherichia Coli. Nat. Commun. 2020, 11 (1), 3109 10.1038/s41467-020-16946-7. 32561741 PMC7305307

[ref326] Shintani M.; Sanchez Z. K.; Kimbara K. Genomics of Microbial Plasmids: Classification and Identification Based on Replication and Transfer Systems and Host Taxonomy. Front. Microbiol. 2015, 6, 10.3389/fmicb.2015.00242. PMC4379921 25873913

[ref327] Summers D. Timing, Self-control and a Sense of Direction Are the Secrets of Multicopy Plasmid Stability. Mol. Microbiol. 1998, 29 (5), 1137–1145. 10.1046/j.1365-2958.1998.01012.x. 9767582

[ref328] Garner E. C.; Campbell C. S.; Weibel D. B.; Mullins R. D. Reconstitution of DNA Segregation Driven by Assembly of a Prokaryotic Actin Homolog. Science 2007, 315 (5816), 1270–1274. 10.1126/science.1138527. 17332412 PMC2851738

[ref329] Havey J. C.; Vecchiarelli A. G.; Funnell B. E. ATP-Regulated Interactions between P1 ParA, ParB and Non-Specific DNA That Are Stabilized by the Plasmid Partition Site, parS. Nucleic Acids Res. 2012, 40 (2), 801–812. 10.1093/nar/gkr747. 21965538 PMC3258138

[ref330] Shin J.; Cherstvy A. G.; Metzler R. Mixing and Segregation of Ring Polymers: Spatial Confinement and Molecular Crowding Effects. New J. Phys. 2014, 16 (5), 053047 10.1088/1367-2630/16/5/053047.

[ref331] Jun S. Polymer Physics for Understanding Bacterial Chromosomes. In Bacterial Chromatin; Dame R. T., Dorman C. J., Eds.; Springer Netherlands: Dordrecht, 2010; pp 97–116. 10.1007/978-90-481-3473-1_6.

[ref332] Chen Y.; Yu W.; Wang J.; Luo K. Polymer Segregation under Confinement: Influences of Macromolecular Crowding and the Interaction between the Polymer and Crowders. J. Chem. Phys. 2015, 143 (13), 134904 10.1063/1.4932370. 26450331

[ref333] Di Ventura B.; Knecht B.; Andreas H.; Godinez W. J.; Fritsche M.; Rohr K.; Nickel W.; Heermann D. W.; Sourjik V. Chromosome Segregation by the *Escherichia Coli* Min System. Mol. Syst. Biol. 2013, 9 (1), 686 10.1038/msb.2013.44. 24022004 PMC3792344

[ref334] Popp D.; Robinson R. C. Many Ways to Build an Actin Filament: Actin Filament Systems. Mol. Microbiol. 2011, 80 (2), 300–308. 10.1111/j.1365-2958.2011.07599.x. 21362063

[ref335] Becker E.; Herrera N. C.; Gunderson F. Q.; Derman A. I.; Dance A. L.; Sims J.; Larsen R. A.; Pogliano J. DNA Segregation by the Bacterial Actin AlfA during Bacillus Subtilis Growth and Development. EMBO J. 2006, 25 (24), 5919–5931. 10.1038/sj.emboj.7601443. 17139259 PMC1698890

[ref336] Wong G. C. L.; Pollack L. Electrostatics of Strongly Charged Biological Polymers: Ion-Mediated Interactions and Self-Organization in Nucleic Acids and Proteins. Annu. Rev. Phys. Chem. 2010, 61 (1), 171–189. 10.1146/annurev.physchem.58.032806.104436. 20055668

[ref337] Popp D.; Gov N. S.; Iwasa M.; Maéda Y. Effect of Short-Range Forces on the Length Distribution of Fibrous Cytoskeletal Proteins. Biopolymers 2008, 89 (9), 711–721. 10.1002/bip.20999. 18412138

[ref338] Salje J.; Zuber B.; Löwe J. Electron Cryomicroscopy of *E. Coli* Reveals Filament Bundles Involved in Plasmid DNA Segregation. Science 2009, 323 (5913), 509–512. 10.1126/science.1164346. 19095899

[ref339] Popp D.; Narita A.; Iwasa M.; Maéda Y.; Robinson R. C. Molecular Mechanism of Bundle Formation by the Bacterial Actin ParM. Biochem. Biophys. Res. Commun. 2010, 391 (4), 1598–1603. 10.1016/j.bbrc.2009.12.078. 20026051

[ref340] Kueh H. Y.; Mitchison T. J. Structural Plasticity in Actin and Tubulin Polymer Dynamics. Science 2009, 325 (5943), 960–963. 10.1126/science.1168823. 19696342 PMC2864651

[ref341] Popp D.; Narita A.; Ghoshdastider U.; Maeda K.; Maéda Y.; Oda T.; Fujisawa T.; Onishi H.; Ito K.; Robinson R. C. Polymeric Structures and Dynamic Properties of the Bacterial Actin AlfA. J. Mol. Biol. 2010, 397 (4), 1031–1041. 10.1016/j.jmb.2010.02.010. 20156449

[ref342] Popp D.; Xu W.; Narita A.; Brzoska A. J.; Skurray R. A.; Firth N.; Goshdastider U.; Maéda Y.; Robinson R. C.; Schumacher M. A. Structure and Filament Dynamics of the pSK41 Actin-like ParM Protein. J. Biol. Chem. 2010, 285 (13), 10130–10140. 10.1074/jbc.M109.071613. 20106979 PMC2843175

[ref343] Tabor C. W.; Tabor H. Polyamines in Microorganisms. Microbiol. Rev. 1985, 49 (1), 81–99. 10.1128/mr.49.1.81-99.1985. 3157043 PMC373019

[ref344] Tabor C. W.; Tabor H. 1,4-Diaminobutane (Putrescine), Spermidine, and Spermine. Annu. Rev. Biochem. 1976, 45 (1), 285–306. 10.1146/annurev.bi.45.070176.001441. 786151

[ref345] Egan E. S.; Fogel M. A.; Waldor M. K. MicroReview: Divided Genomes: Negotiating the Cell Cycle in Prokaryotes with Multiple Chromosomes: Multiple Chromosomes in Prokaryotes. Mol. Microbiol. 2005, 56 (5), 1129–1138. 10.1111/j.1365-2958.2005.04622.x. 15882408

[ref346] Liu Z.; Capaldi X.; Zeng L.; Zhang Y.; Reyes-Lamothe R.; Reisner W. Confinement Anisotropy Drives Polar Organization of Two DNA Molecules Interacting in a Nanoscale Cavity. Nat. Commun. 2022, 13 (1), 4358 10.1038/s41467-022-31398-x. 35902565 PMC9334635

[ref347] Reyes-Lamothe R.; Tran T.; Meas D.; Lee L.; Li A. M.; Sherratt D. J.; Tolmasky M. E. High-Copy Bacterial Plasmids Diffuse in the Nucleoid-Free Space, Replicate Stochastically and Are Randomly Partitioned at Cell Division. Nucleic Acids Res. 2014, 42 (2), 1042–1051. 10.1093/nar/gkt918. 24137005 PMC3902917

[ref348] Dewachter L.; Bollen C.; Wilmaerts D.; Louwagie E.; Herpels P.; Matthay P.; Khodaparast L.; Khodaparast L.; Rousseau F.; Schymkowitz J.; et al. The Dynamic Transition of Persistence toward the Viable but Nonculturable State during Stationary Phase Is Driven by Protein Aggregation. mBio 2021, 12 (4), e00703-21 10.1128/mBio.00703-21. 34340538 PMC8406143

[ref349] Govers S. K.; Mortier J.; Adam A.; Aertsen A. Protein Aggregates Encode Epigenetic Memory of Stressful Encounters in Individual Escherichia Coli Cells. PLOS Biol. 2018, 16 (8), e2003853 10.1371/journal.pbio.2003853. 30153247 PMC6112618

[ref350] Gupta A.; Lloyd-Price J.; Neeli-Venkata R.; Oliveira S. M. D.; Ribeiro A. S. In Vivo Kinetics of Segregation and Polar Retention of MS2-GFP-RNA Complexes in Escherichia Coli. Biophys. J. 2014, 106 (9), 1928–1937. 10.1016/j.bpj.2014.03.035. 24806925 PMC4017294

[ref351] Verstraeten N.; Fauvart M.; Versées W.; Michiels J. The Universally Conserved Prokaryotic GTPases. Microbiol. Mol. Biol. Rev. 2011, 75 (3), 507–542. 10.1128/MMBR.00009-11. 21885683 PMC3165542

[ref352] Bourges A. C.; Lazarev A.; Declerck N.; Rogers K. L.; Royer C. A. Quantitative High-Resolution Imaging of Live Microbial Cells at High Hydrostatic Pressure. Biophys. J. 2020, 118 (11), 2670–2679. 10.1016/j.bpj.2020.04.017. 32402241 PMC7264842

[ref353] Babl L.; Giacomelli G.; Ramm B.; Gelmroth A.-K.; Bramkamp M.; Schwille P. CTP-Controlled Liquid–Liquid Phase Separation of ParB. J. Mol. Biol. 2022, 434 (2), 167401 10.1016/j.jmb.2021.167401. 34902429

[ref354] Ebersbach G.; Briegel A.; Jensen G. J.; Jacobs-Wagner C. A Self-Associating Protein Critical for Chromosome Attachment, Division, and Polar Organization in Caulobacter. Cell 2008, 134 (6), 956–968. 10.1016/j.cell.2008.07.016. 18805089 PMC2614312

[ref355] Bowman G. R.; Comolli L. R.; Zhu J.; Eckart M.; Koenig M.; Downing K. H.; Moerner W. E.; Earnest T.; Shapiro L. A Polymeric Protein Anchors the Chromosomal Origin/ParB Complex at a Bacterial Cell Pole. Cell 2008, 134 (6), 945–955. 10.1016/j.cell.2008.07.015. 18805088 PMC2745220

[ref356] Laloux G.; Jacobs-Wagner C. Spatiotemporal Control of PopZ Localization through Cell Cycle–Coupled Multimerization. J. Cell Biol. 2013, 201 (6), 827–841. 10.1083/jcb.201303036. 23751494 PMC3678156

[ref357] Graumann P. L. Chromosome Architecture and Segregation in Prokaryotic Cells. Microb. Physiol. 2015, 24 (5–6), 291–300. 10.1159/000369100. 25732333

[ref358] Gruber S.; Errington J. Recruitment of Condensin to Replication Origin Regions by ParB/SpoOJ Promotes Chromosome Segregation in B. Subtilis. Cell 2009, 137 (4), 685–696. 10.1016/j.cell.2009.02.035. 19450516

[ref359] Tran N. T.; Laub M. T.; Le T. B. K. SMC Progressively Aligns Chromosomal Arms in Caulobacter Crescentus but Is Antagonized by Convergent Transcription. Cell Rep. 2017, 20 (9), 2057–2071. 10.1016/j.celrep.2017.08.026. 28854358 PMC5583512

[ref360] Karaboja X.; Ren Z.; Brandão H. B.; Paul P.; Rudner D. Z.; Wang X. XerD Unloads Bacterial SMC Complexes at the Replication Terminus. Mol. Cell 2021, 81 (4), 756–766.e8. 10.1016/j.molcel.2020.12.027. 33472056 PMC7897262

[ref361] Mäkelä J.; Sherratt D. J. Organization of the Escherichia Coli Chromosome by a MukBEF Axial Core. Mol. Cell 2020, 78 (2), 250–260.e5. 10.1016/j.molcel.2020.02.003. 32097603 PMC7163298

[ref362] Nolivos S.; Upton A. L.; Badrinarayanan A.; Müller J.; Zawadzka K.; Wiktor J.; Gill A.; Arciszewska L.; Nicolas E.; Sherratt D. MatP Regulates the Coordinated Action of Topoisomerase IV and MukBEF in Chromosome Segregation. Nat. Commun. 2016, 7 (1), 10466 10.1038/ncomms10466. 26818444 PMC4738335

[ref363] Nicolas E.; Upton A. L.; Uphoff S.; Henry O.; Badrinarayanan A.; Sherratt D. The SMC Complex MukBEF Recruits Topoisomerase IV to the Origin of Replication Region in Live Escherichia Coli. mBio 2014, 5 (1), e01001–13. 10.1128/mBio.01001-13. 24520061 PMC3950513

[ref364] Tadesse S.; Graumann P. L. Differential and Dynamic Localization of Topoisomerases in *Bacillus Subtilis*. J. Bacteriol. 2006, 188 (8), 3002–3011. 10.1128/JB.188.8.3002-3011.2006. 16585761 PMC1446999

[ref365] Männik J.; Castillo D. E.; Yang D.; Siopsis G.; Männik J. The Role of MatP, ZapA and ZapB in Chromosomal Organization and Dynamics in *Escherichia Coli*. Nucleic Acids Res. 2016, 44 (3), 1216–1226. 10.1093/nar/gkv1484. 26762981 PMC4756834

[ref366] Ozaki S.; Jenal U.; Katayama T. Novel Divisome-Associated Protein Spatially Coupling the Z-Ring with the Chromosomal Replication Terminus in Caulobacter Crescentus. mBio 2020, 11 (2), e00487–20. 10.1128/mBio.00487-20. 32345642 PMC7188993

[ref367] Cho H.; McManus H. R.; Dove S. L.; Bernhardt T. G. Nucleoid Occlusion Factor SlmA Is a DNA-Activated FtsZ Polymerization Antagonist. Proc. Natl. Acad. Sci. U. S. A. 2011, 108 (9), 3773–3778. 10.1073/pnas.1018674108. 21321206 PMC3048121

[ref368] Haeusser D. P.; Margolin W. Splitsville: Structural and Functional Insights into the Dynamic Bacterial Z Ring. Nat. Rev. Microbiol 2016, 14 (5), 305–319. 10.1038/nrmicro.2016.26. 27040757 PMC5290750

[ref369] Du S.; Lutkenhaus J. At the Heart of Bacterial Cytokinesis: The Z Ring. Trends Microbiol 2019, 27 (9), 781–791. 10.1016/j.tim.2019.04.011. 31171437 PMC6831097

[ref370] Ortiz C.; Natale P.; Cueto L.; Vicente M. The Keepers of the Ring: Regulators of FtsZ Assembly. FEMS Microbiol Rev. 2016, 40 (1), 57–67. 10.1093/femsre/fuv040. 26377318

[ref371] Zorrilla S.; Monterroso B.; Robles-Ramos M.-Á.; Margolin W.; Rivas G. FtsZ. Interactions and Biomolecular Condensates as Potential Targets for New Antibiotics. Antibiotics 2021, 10 (3), 254 10.3390/antibiotics10030254. 33806332 PMC7999717

[ref372] Rivas G.; Alfonso C.; Jimenez M.; Monterroso B.; Zorrilla S. Macromolecular Interactions of the Bacterial Division FtsZ Protein: From Quantitative Biochemistry and Crowding to Reconstructing Minimal Divisomes in the Test Tube. Biophys Rev. 2013, 5 (2), 63–77. 10.1007/s12551-013-0115-1. 28510160 PMC5418439

[ref373] Rivas G.; Fernandez J. A.; Minton A. P. Direct Observation of the Enhancement of Noncooperative Protein Self-Assembly by Macromolecular Crowding: Indefinite Linear Self-Association of Bacterial Cell Division Protein FtsZ.. Proc. Natl. Acad. Sci. U A 2001, 98 (6), 3150–3155. 10.1073/pnas.051634398. PMC30622 11248047

[ref374] Rivas G.; López A.; Mingorance J.; Ferrándiz M. J.; Zorrilla S.; Minton A. P.; Vicente M.; Andreu J. M. Magnesium-Induced Linear Self-Association of the FtsZ Bacterial Cell Division Protein Monomer. The Primary Steps for FtsZ Assembly. J. Biol. Chem. 2000, 275 (16), 11740–11749. 10.1074/jbc.275.16.11740. 10766796

[ref375] Mingorance J.; Rivas G.; Vélez M.; Gómez-Puertas P.; Vicente M. Strong FtsZ Is with the Force: Mechanisms to Constrict Bacteria. Trends Microbiol 2010, 18 (8), 348–356. 10.1016/j.tim.2010.06.001. 20598544

[ref376] Naddaf L.; Sayyed-Ahmad A. Intracellular Crowding Effects on the Self-Association of the Bacterial Cell Division Protein FtsZ. Arch. Biochem. Biophys. 2014, 564, 12–19. 10.1016/j.abb.2014.08.016. 25218002

[ref377] Popp D.; Iwasa M.; Narita A.; Erickson H. P.; Maeda Y. FtsZ Condensates: An in Vitro Electron Microscopy Study. Biopolymers 2009, 91 (5), 340–350. 10.1002/bip.21136. 19137575 PMC2731876

[ref378] Monterroso B.; Reija B.; Jimenez M.; Zorrilla S.; Rivas G. Charged Molecules Modulate the Volume Exclusion Effects Exerted by Crowders on FtsZ Polymerization. PLoS One 2016, 11 (2), e0149060 10.1371/journal.pone.0149060. 26870947 PMC4752323

[ref379] Popp D.; Iwasa M.; Erickson H. P.; Narita A.; Maéda Y.; Robinson R. C. Suprastructures and Dynamic Properties of Mycobacterium Tuberculosis FtsZ. J. Biol. Chem. 2010, 285 (15), 11281–11289. 10.1074/jbc.M109.084079. 20139085 PMC2857006

[ref380] Erickson H. P.; Anderson D. E.; Osawa M. FtsZ in Bacterial Cytokinesis: Cytoskeleton and Force Generator All in One. Microbiol Mol. Biol. Rev. 2010, 74 (4), 504–528. 10.1128/MMBR.00021-10. 21119015 PMC3008173

[ref381] Whitley K. D.; Jukes C.; Tregidgo N.; Karinou E.; Almada P.; Cesbron Y.; Henriques R.; Dekker C.; Holden S. FtsZ. Treadmilling Is Essential for Z-Ring Condensation and Septal Constriction Initiation in Bacillus Subtilis Cell Division. Nat. Commun. 2021, 12 (1), 2448 10.1038/s41467-021-22526-0. 33907196 PMC8079713

[ref382] Squyres G. R.; Holmes M. J.; Barger S. R.; Pennycook B. R.; Ryan J.; Yan V. T.; Garner E. C. Single-Molecule Imaging Reveals That Z-Ring Condensation Is Essential for Cell Division in Bacillus Subtilis. Nat. Microbiol. 2021, 6 (5), 553–562. 10.1038/s41564-021-00878-z. 33737746 PMC8085161

[ref383] Raskin D. M.; de Boer P. A. MinDE-Dependent Pole-to-Pole Oscillation of Division Inhibitor MinC in Escherichia Coli. J. Bacteriol. 1999, 181 (20), 6419–6424. 10.1128/JB.181.20.6419-6424.1999. 10515933 PMC103778

[ref384] Raskin D. M.; de Boer P. A. Rapid Pole-to-Pole Oscillation of a Protein Required for Directing Division to the Middle of Escherichia Coli. Proc. Natl. Acad. Sci. U. S. A. 1999, 96 (9), 4971–4976. 10.1073/pnas.96.9.4971. 10220403 PMC21801

[ref385] Shiomi D.; Margolin W. The C-Terminal Domain of MinC Inhibits Assembly of the Z Ring in Escherichia Coli. J. Bacteriol. 2007, 189 (1), 236–243. 10.1128/JB.00666-06. 17085577 PMC1797224

[ref386] Dajkovic A.; Lan G.; Sun S. X.; Wirtz D.; Lutkenhaus J. MinC Spatially Controls Bacterial Cytokinesis by Antagonizing the Scaffolding Function of FtsZ. Curr. Biol. 2008, 18 (4), 235–244. 10.1016/j.cub.2008.01.042. 18291654

[ref387] Shen B.; Lutkenhaus J. Examination of the Interaction between FtsZ and MinC ^N^ in *E. Coli* Suggests How MinC Disrupts Z Rings. Mol. Microbiol. 2010, 75 (5), 1285–1298. 10.1111/j.1365-2958.2010.07055.x. 20132438

[ref388] Martos A.; Raso A.; Jimenez M.; Petrasek Z.; Rivas G.; Schwille P. FtsZ Polymers Tethered to the Membrane by ZipA Are Susceptible to Spatial Regulation by Min Waves. Biophys. J. 2015, 108 (9), 2371–2383. 10.1016/j.bpj.2015.03.031. 25954894 PMC4423045

[ref389] Tonthat N. K.; Milam S. L.; Chinnam N.; Whitfill T.; Margolin W.; Schumacher M. A. SlmA Forms a Higher-Order Structure on DNA That Inhibits Cytokinetic Z-Ring Formation over the Nucleoid. Proc. Natl. Acad. Sci. U. S. A. 2013, 110 (26), 10586–10591. 10.1073/pnas.1221036110. 23754405 PMC3696773

[ref390] Bernhardt T. G.; de Boer P. A. SlmA, a Nucleoid-Associated, FtsZ Binding Protein Required for Blocking Septal Ring Assembly over Chromosomes in E. Coli. Mol. Cell 2005, 18 (5), 555–564. 10.1016/j.molcel.2005.04.012. 15916962 PMC4428309

[ref391] Cabre E. J.; Monterroso B.; Alfonso C.; Sanchez-Gorostiaga A.; Reija B.; Jimenez M.; Vicente M.; Zorrilla S.; Rivas G. The Nucleoid Occlusion SlmA Protein Accelerates the Disassembly of the FtsZ Protein Polymers without Affecting Their GTPase Activity. PLoS One 2015, 10 (5), e0126434 10.1371/journal.pone.0126434. 25950808 PMC4423959

[ref392] Bailey M. W.; Bisicchia P.; Warren B. T.; Sherratt D. J.; Männik J. Evidence for Divisome Localization Mechanisms Independent of the Min System and SlmA in Escherichia Coli. PLoS Genet. 2014, 10 (8), e1004504 10.1371/journal.pgen.1004504. 25101671 PMC4125044

[ref393] Reija B.; Monterroso B.; Jimenez M.; Vicente M.; Rivas G.; Zorrilla S. Development of a Homogeneous Fluorescence Anisotropy Assay to Monitor and Measure FtsZ Assembly in Solution. Anal. Biochem. 2011, 418 (1), 89–96. 10.1016/j.ab.2011.07.001. 21802401

[ref394] Mellouli S.; Monterroso B.; Vutukuri H. R.; te Brinke E.; Chokkalingam V.; Rivas G.; Huck W. T. S. Self-Organization of the Bacterial Cell-Division Protein FtsZ in Confined Environments. Soft Matter 2013, 9, 10493–10500. 10.1039/c3sm51163d.

[ref395] Groen J.; Foschepoth D.; Te Brinke E.; Boersma A. J.; Imamura H.; Rivas G.; Heus H. A.; Huck W. T. S. Associative Interactions in Crowded Solutions of Biopolymers Counteract Depletion Effects. J. Am. Chem. Soc. 2015, 137 (40), 13041–13048. 10.1021/jacs.5b07898. 26383885

[ref396] Kohyama S.; Merino-Salomón A.; Schwille P. In Vitro Assembly, Positioning and Contraction of a Division Ring in Minimal Cells. Nat. Commun. 2022, 13 (1), 6098 10.1038/s41467-022-33679-x. 36243816 PMC9569390

[ref397] Arjunan S. N. V.; Tomita M. A New Multicompartmental Reaction-Diffusion Modeling Method Links Transient Membrane Attachment of E. Coli MinE to E-Ring Formation. Syst. Synth. Biol. 2010, 4 (1), 35–53. 10.1007/s11693-009-9047-2. 20012222 PMC2816228

[ref398] Loose M.; Fischer-Friedrich E.; Ries J.; Kruse K.; Schwille P. Spatial Regulators for Bacterial Cell Division Self-Organize into Surface Waves in Vitro. Science 2008, 320 (5877), 789–792. 10.1126/science.1154413. 18467587

[ref399] Chiang Y.-L.; Chang Y.-C.; Chiang I.-C.; Mak H.-M.; Hwang I.-S.; Shih Y.-L. Atomic Force Microscopy Characterization of Protein Fibrils Formed by the Amyloidogenic Region of the Bacterial Protein MinE on Mica and a Supported Lipid Bilayer. PLoS One 2015, 10 (11), e0142506 10.1371/journal.pone.0142506. 26562523 PMC4642933

[ref400] Monterroso B.; Robles-Ramos M. A.; Zorrilla S.; Rivas G. Reconstituting Bacterial Cell Division Assemblies in Crowded, Phase-Separated Media. Methods Enzym. 2021, 646, 19–49. 10.1016/bs.mie.2020.06.012. 33453926

[ref401] Monterroso B.; Zorrilla S.; Sobrinos-Sanguino M.; Keating C. D.; Rivas G. Microenvironments Created by Liquid-Liquid Phase Transition Control the Dynamic Distribution of Bacterial Division FtsZ Protein. Sci. Rep 2016, 6, 35140 10.1038/srep35140. 27725777 PMC5057132

[ref402] Keating C. D. Aqueous Phase Separation as a Possible Route to Compartmentalization of Biological Molecules. Acc. Chem. Res. 2012, 45 (12), 2114–2124. 10.1021/ar200294y. 22330132 PMC3525015

[ref403] Helfrich M. R.; El-Kouedi M.; Etherton M. R.; Keating C. D. Partitioning and Assembly of Metal Particles and Their Bioconjugates in Aqueous Two-Phase Systems. Langmuir 2005, 21 (18), 8478–8486. 10.1021/la051220z. 16114960

[ref404] Godino E.; Doerr A.; Danelon C. Min Waves without MinC Can Pattern FtsA-Anchored FtsZ Filaments on Model Membranes. Commun. Biol. 2022, 5 (1), 675 10.1038/s42003-022-03640-1. 35798943 PMC9262947

[ref405] Sobrinos-Sanguino M.; Zorrilla S.; Keating C. D.; Monterroso B.; Rivas G. Encapsulation of a Compartmentalized Cytoplasm Mimic within a Lipid Membrane by Microfluidics. Chem. Commun. 2017, 53, 4775–4778. 10.1039/C7CC01289F. 28361149

[ref406] Gardner K. A. J. A.; Moore D. A.; Erickson H. P. The C-Terminal Linker of *Escherichia Coli* FtsZ Functions as an Intrinsically Disordered Peptide: FtsZ Linker Is an Intrinsically Disordered Peptide. Mol. Microbiol. 2013, 89 (2), 264–275. 10.1111/mmi.12279. 23714328 PMC3725778

[ref407] Buske P. J.; Levin P. A. A Flexible C-Terminal Linker Is Required for Proper FtsZ Assembly in Vitro and Cytokinetic Ring Formation in Vivo. Mol. Microbiol. 2013, 89 (2), 249–263. 10.1111/mmi.12272. 23692518 PMC3708608

[ref408] Fare C. M.; Villani A.; Drake L. E.; Shorter J. Higher-Order Organization of Biomolecular Condensates. Open Biol. 2021, 11 (6), 210137 10.1098/rsob.210137. 34129784 PMC8205532

[ref409] Yu X. C.; Margolin W.; Gonzalez-Garay M. L.; Cabral F. Vinblastine Induces an Interaction between FtsZ and Tubulin in Mammalian Cells. J. Cell Sci. 1999, 112 (14), 2301–2311. 10.1242/jcs.112.14.2301. 10381386

[ref410] Yu J.; Liu Y.; Yin H.; Chang Z. Regrowth-Delay Body as a Bacterial Subcellular Structure Marking Multidrug-Tolerant Persisters. Cell Discov 2019, 5, 8 10.1038/s41421-019-0080-3. 30675381 PMC6341109

[ref411] Thanbichler M.; Shapiro L. MipZ, a Spatial Regulator Coordinating Chromosome Segregation with Cell Division in Caulobacter. Cell 2006, 126 (1), 147–162. 10.1016/j.cell.2006.05.038. 16839883

[ref412] Wu L. J.; Errington J. Coordination of Cell Division and Chromosome Segregation by a Nucleoid Occlusion Protein in Bacillus Subtilis. Cell 2004, 117 (7), 915–925. 10.1016/j.cell.2004.06.002. 15210112

[ref413] Yu Y.; Zhou J.; Gueiros-Filho F. J.; Kearns D. B.; Jacobson S. C. Noc Corrals Migration of FtsZ Protofilaments during Cytokinesis in Bacillus Subtilis. mBio 2021, 12 (1), e02964-20 10.1128/mBio.02964-20. 33531398 PMC7858058

[ref414] Jalal A. S. B.; Tran N. T.; Wu L. J.; Ramakrishnan K.; Rejzek M.; Gobbato G.; Stevenson C. E. M.; Lawson D. M.; Errington J.; Le T. B. K. CTP Regulates Membrane-Binding Activity of the Nucleoid Occlusion Protein Noc. Mol. Cell 2021, 81 (17), 3623–3636.e6. 10.1016/j.molcel.2021.06.025. 34270916 PMC8429893

[ref415] Riback J. A.; Katanski C. D.; Kear-Scott J. L.; Pilipenko E. V.; Rojek A. E.; Sosnick T. R.; Drummond D. A. Stress-Triggered Phase Separation Is an Adaptive, Evolutionarily Tuned Response. Cell 2017, 168 (6), 1028–1040 e19. 10.1016/j.cell.2017.02.027. 28283059 PMC5401687

[ref416] Basile W.; Salvatore M.; Bassot C.; Elofsson A. Why Do Eukaryotic Proteins Contain More Intrinsically Disordered Regions?. PLOS Comput. Biol. 2019, 15 (7), e1007186 10.1371/journal.pcbi.1007186. 31329574 PMC6675126

[ref417] Van Der Lee R.; Buljan M.; Lang B.; Weatheritt R. J.; Daughdrill G. W.; Dunker A. K.; Fuxreiter M.; Gough J.; Gsponer J.; Jones D. T.; et al. Classification of Intrinsically Disordered Regions and Proteins. Chem. Rev. 2014, 114 (13), 6589–6631. 10.1021/cr400525m. 24773235 PMC4095912

[ref418] Zhang C.; Zhao W.; Duvall S. W.; Kowallis K. A.; Childers W. S. Regulation of the Activity of the Bacterial Histidine Kinase PleC by the Scaffolding Protein PodJ. J. Biol. Chem. 2022, 298 (4), 101683 10.1016/j.jbc.2022.101683. 35124010 PMC8980812

[ref419] Fisher R. A.; Gollan B.; Helaine S. Persistent Bacterial Infections and Persister Cells. Nat. Rev. Microbiol 2017, 15 (8), 453–464. 10.1038/nrmicro.2017.42. 28529326

[ref420] Balaban N. Q.; Merrin J.; Chait R.; Kowalik L.; Leibler S. Bacterial Persistence as a Phenotypic Switch. Science 2004, 305 (5690), 1622–1625. 10.1126/science.1099390. 15308767

[ref421] Alberti S.; Dormann D. Liquid-Liquid Phase Separation in Disease. Annu. Rev. Genet 2019, 53, 171–194. 10.1146/annurev-genet-112618-043527. 31430179

[ref422] Pu Y.; Li Y.; Jin X.; Tian T.; Ma Q.; Zhao Z.; Lin S.-Y.; Chen Z.; Li B.; Yao G.; et al. ATP-Dependent Dynamic Protein Aggregation Regulates Bacterial Dormancy Depth Critical for Antibiotic Tolerance. Mol. Cell 2019, 73 (1), 143–156.e4. 10.1016/j.molcel.2018.10.022. 30472191

[ref423] Bobst E. V.; Bobst A. M.; Perrino F. W.; Meyer R. R.; Rein D. C. Variability in the Nucleic Acid Binding Site Size and the Amount of Single-Stranded DNA-Binding Protein in *Escherichia Coli*. FEBS Lett. 1985, 181 (1), 133–137. 10.1016/0014-5793(85)81128-5. 2982651

[ref424] Kozlov A. G.; Weiland E.; Mittal A.; Waldman V.; Antony E.; Fazio N.; Pappu R. V.; Lohman T. M. Intrinsically Disordered C-Terminal Tails of E. Coli Single-Stranded DNA Binding Protein Regulate Cooperative Binding to Single-Stranded DNA. J. Mol. Biol. 2015, 427 (4), 763–774. 10.1016/j.jmb.2014.12.020. 25562210 PMC4419694

[ref425] Janissen R.; Arens M. M. A.; Vtyurina N. N.; Rivai Z.; Sunday N. D.; Eslami-Mossallam B.; Gritsenko A. A.; Laan L.; de Ridder D.; Artsimovitch I.; et al. Global DNA Compaction in Stationary-Phase Bacteria Does Not Affect Transcription. Cell 2018, 174 (5), 1188–1199 e14. 10.1016/j.cell.2018.06.049. 30057118 PMC6108918

[ref426] Stephani K.; Weichart D.; Hengge R. Dynamic Control of Dps Protein Levels by ClpXP and ClpAP Proteases in Escherichia Coli: Regulated Proteolysis of Dps Protein. Mol. Microbiol. 2003, 49 (6), 1605–1614. 10.1046/j.1365-2958.2003.03644.x. 12950924

[ref427] Oberto J.; Nabti S.; Jooste V.; Mignot H.; Rouviere-Yaniv J. The HU Regulon Is Composed of Genes Responding to Anaerobiosis, Acid Stress, High Osmolarity and SOS Induction. PLoS One 2009, 4 (2), e4367 10.1371/journal.pone.0004367. 19194530 PMC2634741

[ref428] Krypotou E.; Townsend G. E.; Gao X.; Tachiyama S.; Liu J.; Pokorzynski N. D.; Goodman A. L.; Groisman E. A. Bacteria Require Phase Separation for Fitness in the Mammalian Gut. Science 2023, 379 (6637), 1149–1156. 10.1126/science.abn7229. 36927025 PMC10148683

[ref429] Pattanayak G. K.; Liao Y.; Wallace E. W. J.; Budnik B.; Drummond D. A.; Rust M. J. Daily Cycles of Reversible Protein Condensation in Cyanobacteria. Cell Rep. 2020, 32 (7), 108032 10.1016/j.celrep.2020.108032. 32814039 PMC10005845

[ref430] Racki L. R.; Tocheva E. I.; Dieterle M. G.; Sullivan M. C.; Jensen G. J.; Newman D. K. Polyphosphate Granule Biogenesis Is Temporally and Functionally Tied to Cell Cycle Exit during Starvation in Pseudomonas Aeruginosa. Proc. Natl. Acad. Sci. U A 2017, 114 (12), E2440–E2449. 10.1073/pnas.1615575114. PMC5373386 28265086

[ref431] Kriel A.; Bittner A. N.; Kim S. H.; Liu K.; Tehranchi A. K.; Zou W. Y.; Rendon S.; Chen R.; Tu B. P.; Wang J. D. Direct Regulation of GTP Homeostasis by (p)ppGpp: A Critical Component of Viability and Stress Resistance. Mol. Cell 2012, 48 (2), 231–241. 10.1016/j.molcel.2012.08.009. 22981860 PMC3483369

[ref432] Zhang Y.; Zbornikova E.; Rejman D.; Gerdes K. Novel (p)ppGpp Binding and Metabolizing Proteins of Escherichia Coli. mBio 2018, 9 ( (2), ), 10.1128/mBio.02188-17. PMC5845004 29511080

[ref433] Harms A.; Maisonneuve E.; Gerdes K. Mechanisms of Bacterial Persistence during Stress and Antibiotic Exposure. Science 2016, 354 ( (6318), ), 10.1126/science.aaf4268. 27980159

